# Supplementary material for: Saddles as rotational locks within shape-assisted self-assembled nanosheets
Source: Nat Commun. 2023 Aug 7;14:4725. doi: 10.1038/s41467-023-40475-8 (PMC10406840; doi:10.1038/s41467-023-40475-8)
Supplement: Supplementary file 1 — Supplementary Information [file 41467_2023_40475_MOESM1_ESM.pdf]

Supplementary Information for:

Saddles as rotational locks within shape-assisted self-assembled nanosheets

Joseph F. Woods[1], Lucía Gallego[1], Amira Maisch[1], Dominik Renggli[1], Corrado Cuocci[2], Olivier Blacque[1], Gunther Steinfeld[3], Andres Kaech[4], Bernhard Spingler[1], Andreas Vargas Jentzsch[5], Michel Rickhaus[1]

[1]Department of Chemistry, University of Zurich, 8057 Zurich, Switzerland.
[2]Institute of Crystallography, CNR, Via Amendola, 122/O, 70126 Bari, Italy.
[3]ELDICO Scientific AG, 5234 Villigen, Switzerland.
[4]Center for Microscopy and Image Analysis, University of Zurich, 8057 Zurich, Switzerland.
[5]SAMS Research Group, University of Strasbourg, Institut Charles Sadron, CNRS, 67200 Strasbourg, France.

*Corresponding author email: michel.rickhaus@chem.uzh.ch

Contents:

Supplementary Discussion

1. **Experimental Procedures**
2. **NMR Characterization**
3. **Variable Temperature ^{1}H NMR Spectra**
4. **Spectroscopic Data**
5. **Microscopy**
6. **Differential Scanning Calorimetry**
7. **X-ray Diffraction**
8. **Micro Electron Diffraction**

Supplementary References

Supplementary Discussion

Experimental Procedures

Overview Schemes

a) **2H-Car-H**

This compound was synthesized following a previously reported procedure[1].

b) **2H-Car-C1**

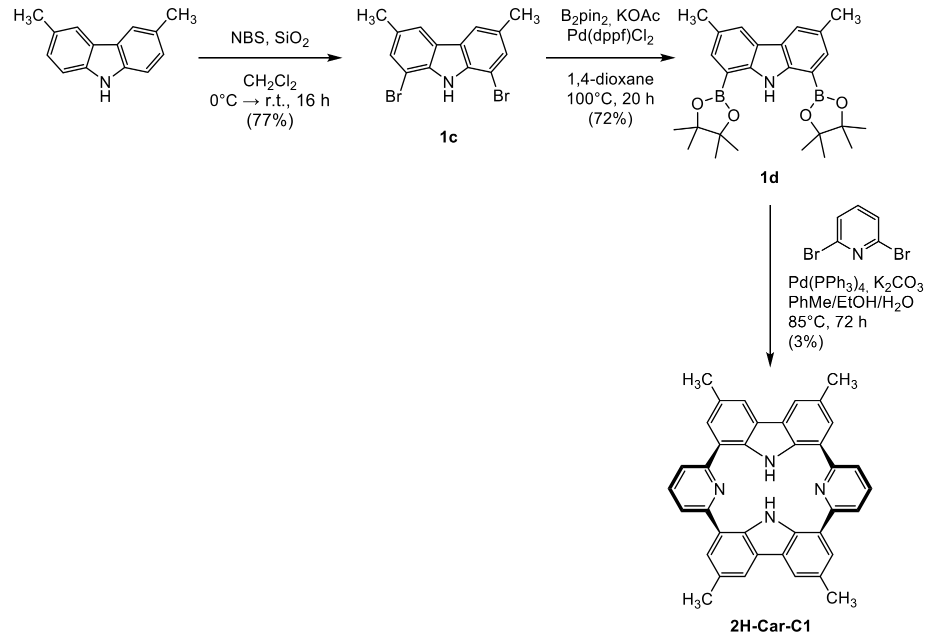

3,6-dimethyl-9*H*-carbazole was purchased commercially and used without further purification.

c) **2H-Car-C2 – 2H-Car-C10**

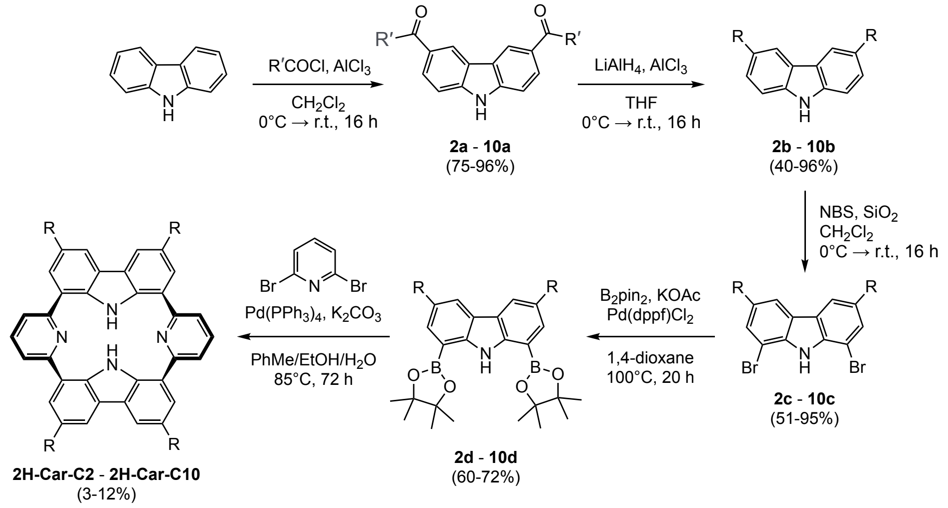

The synthesis of compounds **2a**[2], **4a**[3], **6a**[4], **8a**[1], **2b**[5], **4b**[3], **6b**[4], **8b**[1], **6c**[4], **8c**[1], **6d**[1], **8d**[1], **2H-Car-H**[1], **2H-Car-C6**[1] and **2H-Car-C8**[1] was performed following previously reported procedures. The analytical data were in agreement with those reported in literature.

General procedure A: acylation of carbazole

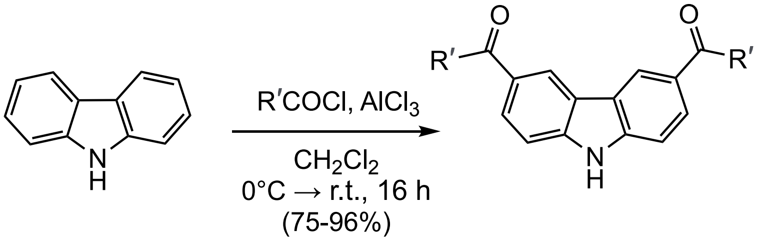

The corresponding commercially available acyl chloride (68.8 mmol, 2.3 eq.) was added to a stirring solution of $AlCl_3$ (3.76 g, 68.8 mmol, 2.3 eq.) in CH_2Cl_2 (150 mL) at 0 °C before adding carbazole (5.00 g, 29.9 mol, 1.0 eq.) in portions. The reaction mixture was warmed to room temperature and stirred for 16 h before being poured onto ice. The solid was filtered off and washed with water and MeOH which yielded the corresponding acylated carbazole as an off-white solid.

1,1'-(9*H*-carbazole-3,6-diyl)bis(ethan-1-one) (**2a**)

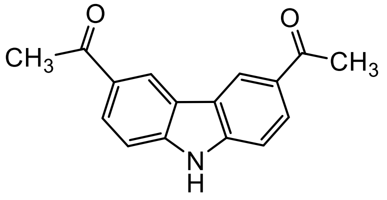

2a

Carbazole (5.00 g, 29.9 mmol, 1.0 eq.) was reacted with acetyl chloride (4.91 mL, 68.8 mmol, 2.3 eq.) following general procedure A to yield carbazole **2a** (5.64 g, 75%) as an off-white solid.

Analytical data for ^{1}H NMR were in agreement with those reported below that are found in literature[2].

^{1}**H NMR** (400 MHz, 298 K, DMSO-d_6): δ = 12.1 (s, 1H), 9.0 (d, J = 1.6 Hz, 2H), 8.1 (dd, J = 1.6, 8.8 Hz, 2H), 7.6 (d, J = 8.4 Hz, 2H), 2.7 (s, 6H) ppm.

1,1'-(9*H*-carbazole-3,6-diyl)bis(propan-1-one) (**3a**)

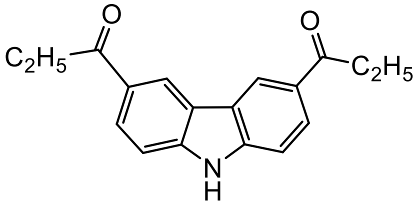

3a

Carbazole (5.00 g, 29.9 mmol, 1.0 eq.) was reacted with propionyl chloride (6.00 mL, 68.8 mmol, 2.3 eq.) following general procedure A to yield carbazole **3a** (8.03 g, 96%) as an off-white solid.

^{1}**H NMR** (400 MHz, 298 K, THF-d_8): δ = 10.97 (s, 1H, N-H), 8.90 (d, J = 1.6 Hz, 2H, H_{Ar}), 8.11 (dd, J_1 = 8.5 Hz, J_2 = 1.7 Hz, 2H, H_{Ar}), 7.48 (d, J = 8.5 Hz, 2H, H_{Ar}), 3.13 (q, J = 7.2 Hz, 4H, H_{C3}), 1.22 (t, J = 7.2 Hz, 6H, H_{C3}) ppm.

^{13}**C NMR** (101 MHz, 298 K, THF-d_8): δ = 199.06, 144.58, 130.80, 127.33, 124.35, 122.41, 111.87, 32.08, 8.99 ppm.
HR-ESI-MS: m/z = 278.11879 [M−H]$^-$ ($C_{18}H_{16}O_2N^-$ requires 278.11865).

1,1'-(9*H*-carbazole-3,6-diyl)bis(butan-1-one) (**4a**)

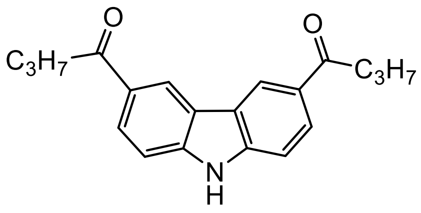

4a

Carbazole (5.00 g, 29.9 mmol, 1.0 eq.) was reacted with butyryl chloride (7.20 mL, 68.8 mmol, 2.3 eq.) following general procedure A to yield carbazole **4a** (7.58 g, 82%) as an off-white solid.

Analytical data for ^{1}H NMR were in agreement with those reported below that are found in literature[3].

^{1}**H NMR** (400 MHz, 298 K, $CDCl_3$): δ = 8.79 (d, J = 1.6 Hz, 2H), 8.68 (s, 1H), 8.15 (dd, J = 1.6 Hz, J = 8.4 Hz, 2H), 7.49 (d, J = 8.4 Hz, 2H), 3.11 (t, J = 7.4 Hz, 4H), 1.86 (m, J = 7.4 Hz, 4H), 1.07 (t, J = 7.4 Hz, 6H) ppm.

1,1'-(9*H*-carbazole-3,6-diyl)bis(pentan-1-one) (**5a**)

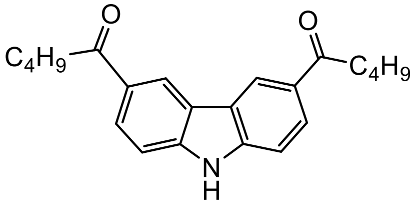

5a

Carbazole (5.00 g, 29.9 mmol, 1.0 eq.) was reacted with pentanoyl chloride (8.16 mL, 68.8 mmol, 2.3 eq.) following general procedure A to yield carbazole **5a** (8.37 g, 83%) as an off-white solid.

^{1}**H NMR** (400 MHz, 298 K, $CDCl_3$): δ = 8.79 (d, J = 1.5 Hz, 2H, H_{Ar}), 8.70 (s, 1H, N-H), 8.15 (dd, J_1 = 8.6 Hz, J_2 = 1.7 Hz, 2H, H_{Ar}), 7.50 (d, J = 8.5 Hz, 2H, H_{Ar}), 3.12 (t, J = 7.5 Hz, 4H, H_{C5}), 1.81 (p, J = 7.5 Hz, 4H, H_{C5}), 1.53–1.44 (m, 4H, H_{C5}), 1.00 (t, J = 7.4 Hz, 6H, H_{C5}) ppm.
^{13}**C NMR** (101 MHz, 298 K, $CDCl_3$): δ = 200.23, 142.95, 130.29, 127.21, 123.49, 121.80, 110.95, 38.51, 27.08, 22.77, 14.17 ppm.
HR-ESI-MS: m/z = 336.19582 [M+H]$^+$ ($C_{22}H_{26}O_2N^+$ requires 336.19581).

1,1'-(9*H*-carbazole-3,6-diyl)bis(hexan-1-one) (**6a**)

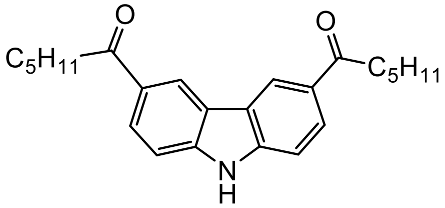

6a

Carbazole **6a** was synthesized following a previously reported procedure.

Analytical data for ^{1}H NMR were in agreement with those reported below that are found in literature[4].

^{1}H NMR (400 MHz, 298 K, DMSO-d_6,): δ = 12.05 (s, 1H), 9.05 (s, 2H), 8.07 (dd, J = 8.4, J = 1.2 Hz, 2H), 7.60 (d, J = 8.8 Hz, 2H), 3.15 (t, J = 7.2 Hz, 4H), 1.71–1.66 (m, 4H), 1.39–1.35 (m, 8H), 0.90 (t, J = 6.8 Hz, 6H) ppm.

1,1'-(9*H*-carbazole-3,6-diyl)bis(heptan-1-one) (**7a**)

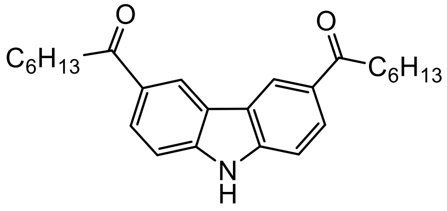

7a

Carbazole (5.00 g, 29.9 mmol, 1.0 eq.) was reacted with pentanoyl chloride (10.6 mL, 68.8 mmol, 2.3 eq.) following general procedure A to yield carbazole **7a** (11.0 g, 94%) as an off-white solid.

^{1}H NMR (400 MHz, 298 K, $CDCl_3$): δ = 8.79 (d, J = 1.6 Hz, 2H, H_{Ar}), 8.68 (s, 1H, N-H), 8.14 (dd, J_1 = 8.6 Hz, J_2 = 1.7 Hz, 2H, H_{Ar}), 7.50 (d, J = 8.5 Hz, 2H, H_{Ar}), 3.12 (t, J = 7.6 Hz, 4H, H_{C7}), 1.82 (p, J = 7.2 Hz, 4H, H_{C7}), 1.49–1.42 (m, 4H, H_{C7}), 1.39–1.31 (m, 8H, H_{C7}), 0.91 (t, J = 7.1 Hz, 6H, H_{C7}) ppm.
^{13}C NMR (101 MHz, 298 K, $CDCl_3$): δ = 200.25, 142.95, 130.29, 127.21, 123.49, 121.81, 110.95, 38.81, 31.88, 29.34, 24.94, 22.73, 14.23 ppm.
HR-ESI-MS: m/z = 392.25811 $[M+H]^+$ ($C_{26}H_{34}O_2N^+$ requires 392.25841).

1,1'-(9H-carbazole-3,6-diyl)bis(octan-1-one) (**8a**)

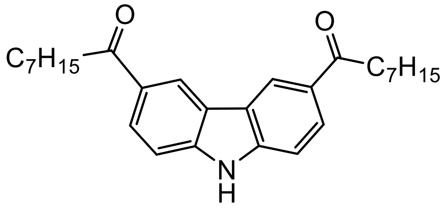

8a

Carbazole **8a** was synthesized following a previously reported procedure[1].

Analytical data were in agreement with those reported in literature.

1,1'-(9*H*-carbazole-3,6-diyl)bis(decan-1-one) (**10a**)

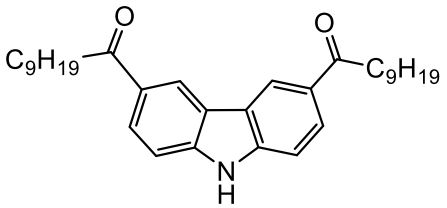

10a

Carbazole (5.00 g, 29.9 mmol, 1.0 eq.) was reacted with pentanoyl chloride (14.3 mL, 68.8 mmol, 2.3 eq.) following general procedure A to yield carbazole **10a** (12.9 g, 91%) as an off-white solid.

^{1}H NMR (400 MHz, 298 K, $CDCl_3$): δ = 8.79 (d, J = 1.7 Hz, 2H, H_{Ar}), 8.59 (s, 1H, N-H), 8.15 (dd, J_1 = 8.6 Hz, J_2 = 1.7 Hz, 2H, H_{Ar}), 7.50 (d, J = 8.5 Hz, 2H, H_{Ar}), 3.11 (t, J = 7.5 Hz, 4H, H_{C10}), 1.82 (p, J = 7.5 Hz, 4H, H_{C10}), 1.49–1.25 (m, 24H, H_{C10}), 0.88 (t, J = 7.0 Hz, 6H, H_{C10}) ppm.

^{13}C NMR (101 MHz, 298 K, $CDCl_3$): δ = 200.30, 142.99, 130.25, 127.20, 123.48, 121.80, 110.97, 38.81, 32.04, 29.71, 29.67, 29.46, 24.99, 22.82, 14.26 ppm (1 aliphatic C is not showing due to overlapping of signals).
HR-ESI-MS: *m/z* = 474.33807 $[M{-}H]^-$ ($C_{32}H_{44}O_2N^-$ requires 474.33826).

General procedure B: reduction of carbonyls

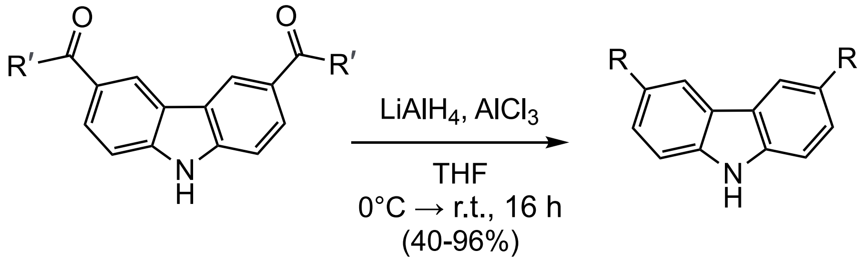

Lithium aluminium hydride solution (1 M in THF, 4.0 eq.) was slowly added to $AlCl_3$ (2.0 eq.) stirring in THF at 0 °C under N_2. In portions, the acylated carbazole (1.0 eq.) was added before the reaction mixture was warmed to room temperature and allowed to stir for 16 h. The reaction was quenched at 0 °C by adding 5% aqueous HCl (10 mL) followed by EtOAc (30 mL) and then passed over a celite plug to remove solids, eluting with EtOAc. The mixture was concentrated *in vacuo* before being extracted with CH_2Cl_2 (100 mL) and washed with brine (100 mL). The organic phase was dried over $MgSO_4$ and the solvent was removed *in vacuo* to afford the corresponding alkyl carbazole as a solid.

3,6-diethyl-9*H*-carbazole (**2b**)

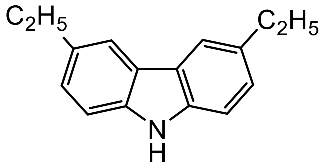

2b

Carbazole **2a** (2.02 g, 8.03 mmol, 1.0 eq.) was reacted with $AlCl_3$ (2.14 g, 16.1 mmol, 2.0 eq.) and $LiAlH_4$ (32.1 mL, 32.1 mmol, 1 M in THF, 4.0 eq.) in THF (160 mL) following general procedure B to yield carbazole **2b** (1.35 g, 75%) as a yellow solid.

Analytical data for ^{1}H NMR were in agreement with those reported below that are found in literature[5].

^{1}H NMR (500 MHz, 298 K, $CDCl_3$): δ = 7.89 (s, 2H), 7.31 (d, *J* = 8.2 Hz, 2H), 7.25 (dd, *J* = 7.9, 1.8 Hz, 2H), 2.83 (q, *J* = 7.6 Hz, 4H), 1.34 (t, *J* = 7.6 Hz, 6H) ppm.

3,6-dipropyl-9*H*-carbazole (**3b**)

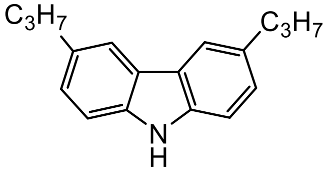

3b

Carbazole **3a** (2.99 g, 10.7 mmol, 1.0 eq.) was reacted with $AlCl_3$ (2.85 g, 21.4 mmol, 2.0 eq.) and $LiAlH_4$ (38.5 mL, 42.8 mmol, 1 M in THF, 4.0 eq.) in THF (220 mL) following general procedure B to yield carbazole **3b** (2.21 g, 82%) as a yellow solid.

Analytical data were in agreement with those reported in literature[6].

3,6-dibutyl-9*H*-carbazole (**4b**)

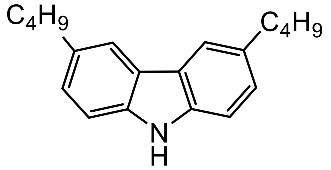

4b

Carbazole **4a** (3.35 g, 10.9 mmol, 1.0 eq.) was reacted with $AlCl_3$ (2.91 g, 21.8 mmol, 2.0 eq.) and $LiAlH_4$ (39.2 mL, 43.6 mmol, 1 M in THF, 4.0 eq.) in THF (220 mL) following general procedure B to yield carbazole **4b** (1.90 g, 62%) as a white solid.

Analytical data for ^{1}H NMR were in agreement with those reported below that are found in literature[3].

^{1}H NMR (400 MHz, 298 K, $CDCl_3$): δ = 7.89 (s, 2H), 7.77 (s, 1H), 7.28 (dd, J = 8 Hz and 0.4 Hz, 2H), 7.24 (dd, J = 8 Hz and 1.2 Hz), 2.81 (t, J = 7.8 Hz, 4H), 1.74 (m, J = 7.6 Hz, 4H), 1.44 (m, J = 7.5 Hz, 4H), 1.00 (t, J = 7.4 Hz, 6H) ppm.

3,6-dipentyl-9*H*-carbazole (**5b**)

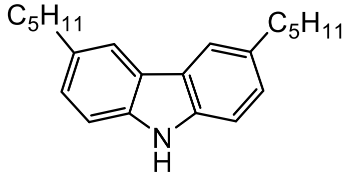

5b

Carbazole **5a** (2.99 g, 8.94 mmol, 1.0 eq.) was reacted with $AlCl_3$ (2.38 g, 17.9 mmol, 2.0 eq.) and $LiAlH_4$ (35.8 mL, 35.8 mmol, 1 M in THF, 4.0 eq.) in THF (180 mL) following general procedure B to yield carbazole **5b** (2.30 g, 83%) as an off-white solid.

^{1}H NMR (400 MHz, 298 K, $CDCl_3$): δ = 7.85 (s (br), 3H, N-H + H_{Ar}), 7.31 (d, J = 8.2 Hz, 2H, H_{Ar}), 7.22 (dd, J_1 = 8.3 Hz, J_2 = 1.7 Hz, 2H, H_{Ar}), 2.77 (t, J = 7.9 Hz, 4H, H_{C5}), 1.71 (p, J = 7.7 Hz, 4H, H_{C5}), 1.39–1.34 (m, 8H, H_{C5}), 0.91 (t, J = 7.1 Hz, 6H, H_{C5}) ppm.
^{13}C NMR (101 MHz, 298 K, $CDCl_3$): δ = 138.39, 133.99, 126.56, 123.57, 119.69, 110.33, 36.18, 32.18, 31.73, 22.79, 14.24.
HR-ESI-MS: m/z = 308.23718 $[M+H]^+$ ($C_{22}H_{30}N^+$ requires 308.23728).

3,6-dihexyl-9*H*-carbazole (**6b**)

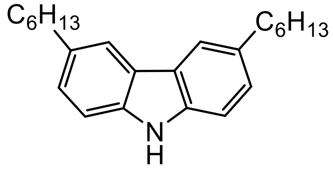

6b

Carbazole **6b** was synthesized following a previously reported procedure.

Analytical data for ^{1}H NMR were in agreement with those reported below that are found in literature[4].

^{1}H NMR (400 MHz, 298 K $CDCl_3$,): δ = 7.84 (s, 2H), 7.71 (s, 1H), 7.26–7.17 (m, 4H), 2.76 (t, J = 7.6 Hz, 4H), 1.71–1.65 (m, 4H), 1.37–1.32 (m, 12H), 0.88 (t, J = 4.8 Hz, 6H) ppm.

3,6-diheptyl-9*H*-carbazole (**7b**)

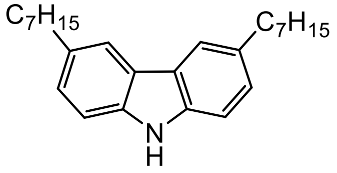

7b

Carbazole **7a** (2.99 g, 7.66 mmol, 1.0 eq.) was reacted with $AlCl_3$ (2.04 g, 15.3 mmol, 2.0 eq.) and $LiAlH_4$ (30.6 mL, 30.6 mmol, 1 M in THF, 4.0 eq.) in THF (150 mL) following general procedure B to yield carbazole **7b** (2.17 g, 78%) as an off-white solid.

^{1}H NMR (400 MHz, 298 K, $CDCl_3$): δ = 7.86 (d, J = 1.5 Hz, 2H, H_{Ar}), 7.83 (s, 1H, N-H), 7.30 (d, J = 8.2 Hz, 2H, H_{Ar}), 7.22 (dd, J_1 = 8.2 Hz, J_2 = 1.7 Hz, 2H, H_{Ar}), 2.77 (t, J = 7.9 Hz, 4H, H_{C7}), 1.71 (p, J = 7.8 Hz, 4H, H_{C7}), 1.39–1.28 (m, 16H, H_{C7}), 0.90 (t, J = 7.0 Hz, 6H, H_{C7}) ppm.
^{13}C NMR (101 MHz, 298 K, $CDCl_3$): δ = 138.38, 133.98, 126.55, 123.56, 119.67, 110.32, 36.21, 32.51, 32.01, 29.50, 29.42, 22.84, 14.26.
HR-ESI-MS: m/z = 364.29976 $[M+H]^+$ ($C_{26}H_{38}N^+$ requires 364.29988).

3,6-dioctyl-9*H*-carbazole (**8b**)

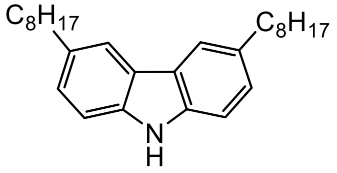

8b

Carbazole **8b** was synthesized following a previously reported procedure[1].

Analytical data were in agreement with those reported in literature.

3,6-didecyl-9*H*-carbazole (**10b**)

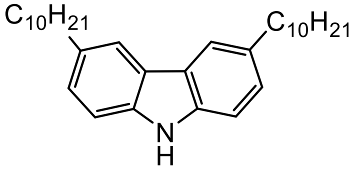

10b

Carbazole **10a** (3.00 g, 6.31 mmol, 1.0 eq.) was reacted with $AlCl_3$ (1.68 g, 12.6 mmol, 2.0 eq.) and $LiAlH_4$ (22.7 mL, 25.2 mmol, 1 M in THF, 4.0 eq.) in THF (120 mL) following general procedure B to yield carbazole **10b** (2.47 g, 88%) as a white solid.

^{1}H NMR (400 MHz, 298 K, $CDCl_3$): δ = 7.85 (d, J = 1.6 Hz, 2H, H_{Ar}), 7.84 (s, 1H, N-H), 7.30 (d, J = 8.2 Hz, 2H, H_{Ar}), 7.22 (dd, J_1 = 8.3 Hz, J_2 = 1.7 Hz, 2H, H_{Ar}), 2.77 (t, J = 7.9 Hz, 4H, H_{C10}), 1.70 (p, J = 7.7 Hz, 4H, H_{C10}), 1.37–1.25 (m, 28H, H_{C10}), 0.88 (t, J = 7.1 Hz, 6H, H_{C10}) ppm.
^{13}C NMR (101 MHz, 298 K, $CDCl_3$): δ = 138.38, 133.99, 126.55, 123.57, 119.68, 110.32, 36.22, 32.51, 32.07, 29.80, 29.76, 29.54, 29.50, 22.84, 14.27 (1 aliphatic C is not showing due to overlapping of signals).
HR-ESI-MS: m/z = 446.37944 $[M-H]^-$ ($C_{32}H_{48}N^-$ requires 446.37922).

General procedure C: bromination of carbazole

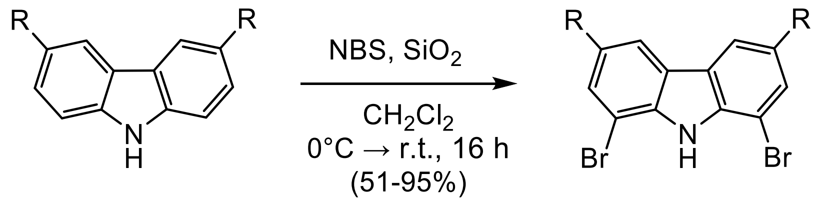

N-bromosuccinimide (2.1 eq.) was slowly added to a stirring solution of silica gel and the corresponding alkyl carbazole (1.0 eq.) in CH_2Cl_2 at 0 °C in the dark. After addition, the reaction mixture was warmed to room temperature and stirred for 16 h. The mixture was then filtered and washed with aqueous NaOH (100 mL, 1 M) and with brine (100 mL). The organic layer was dried over $MgSO_4$ and the solvent was removed *in vacuo*. Purification *via* column chromatography on silica gel (petroleum ether/CH_2Cl_2) afforded the corresponding brominated carbazole.

1,8-dibromo-3,6-dimethyl-9*H*-carbazole (**1c**)

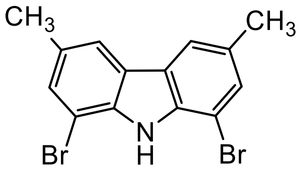

1c

Commercially available 3,6-dimethyl-9*H*-carbazole (1.08 g, 5.52 mmol, 1.0 eq.) was reacted with *N*-bromosuccinimide (2.06 g, 11.6 mmol, 2.1 eq.) and SiO_2 (5 g) in CH_2Cl_2 (200 mL) following general procedure C. The crude mixture was purified *via* column chromatography on silica gel (petroleum ether/CH_2Cl_2, 9:1) to yield carbazole **1c** (1.50 g, 77%) as a white solid.

^{1}H NMR (400 MHz, 298 K, $CDCl_3$): δ = 8.11 (s, 1H, N-H), 7.72 (d, J = 2.0 Hz, 2H, H_{Ar}), 7.42 (d, J = 1.5 Hz, 2H, H_{Ar}), 2.50 (s, 6H, H_{C1}).
^{13}C NMR (101 MHz, 298 K, $CDCl_3$): δ = 136.47, 130.94, 129.85, 124.90, 119.90, 104.07, 21.32.
HR-ESI-MS: m/z = 349.91868 $[M{-}H]^-$ ($C_{14}H_{10}NBr_2^-$ requires 349.91855).

1,8-dibromo-3,6-diethyl-9*H*-carbazole (**2c**)

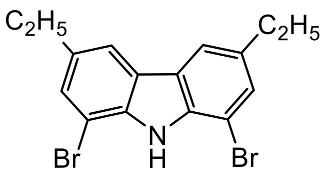

2c

Carbazole **2b** (1.35 g, 6.06 mmol, 1.0 eq.) was reacted with *N*-bromosuccinimide (2.27 g, 12.7 mmol, 2.1 eq.) and SiO_2 (6 g) in CH_2Cl_2 (200 mL) following general procedure C. The crude mixture was purified *via* column chromatography on silica gel (petroleum ether/CH_2Cl_2, 9:1) to yield carbazole **2c** (2.21 g, 95%) as a colorless oil.

^{1}H NMR (400 MHz, 298 K, $CDCl_3$): δ = 8.14 (s, 1H, N-H), 7.77 (d, J = 0.8 Hz, 2H, H_{Ar}), 7.45 (d, J = 1.2 Hz, 2H, H_{Ar}), 2.80 (q, J = 7.6 Hz, 4H, H_{C2}), 1.33 (t, J = 7.6 Hz, 6H, H_{C2}) ppm.
^{13}C NMR (101 MHz, 298 K, $CDCl_3$): δ = 137.67, 136.64, 128.89, 125.05, 118.72, 104.18, 28.87, 16.42.
HR-ESI-MS: m/z = 379.94679 $[M{+}H]^+$ ($C_{16}H_{16}NBr_2^+$ requires 379.96440).

1,8-dibromo-3,6-dipropyl-9*H*-carbazole (**3c**)

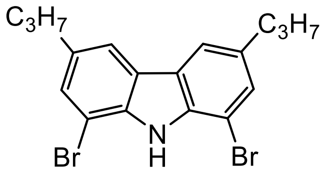

3c

Carbazole **3b** (2.21 g, 8.80 mmol, 1.0 eq.) was reacted with *N*-bromosuccinimide (3.29 g, 18.5 mmol, 2.1 eq.) and SiO_2 (9 g) in CH_2Cl_2 (250 mL) following general procedure C. The crude mixture was purified *via* column chromatography on silica gel (petroleum ether/CH_2Cl_2, 9:1) to yield carbazole **3c** (2.48 g, 69%) as a white solid.

^{1}H NMR (400 MHz, 298 K, $CDCl_3$): δ = 8.13 (s, 1H, N-H), 7.74 (d, J = 1.5 Hz, 2H, H_{Ar}), 7.42 (d, J = 1.4 Hz, 2H, H_{Ar}), 2.73 (t, J = 7.6 Hz, 4H, H_{C3}), 1.78–1.68 (m, 4H, H_{C3}), 0.98 (t, J = 7.3 Hz, 6H, H_{C3}) ppm.
^{13}C NMR (101 MHz, 298 K, $CDCl_3$): δ = 137.64, 136.02, 129.32, 124.96, 119.39, 104.10, 37.95, 25.26, 13.90.
HR-ESI-MS: m/z = 407.99582 $[M+H]^+$ ($C_{18}H_{20}NBr_2^+$ requires 407.99570).

1,8-dibromo-3,6-dibutyl-9*H*-carbazole (**4c**)

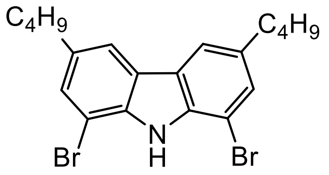

4c

Carbazole **4b** (1.90 g, 6.80 mmol, 1.0 eq.) was reacted with *N*-bromosuccinimide (2.66 g, 15.0 mmol, 2.1 eq.) and SiO_2 (7 g) in CH_2Cl_2 (240 mL) following general procedure C. The crude mixture was purified *via* column chromatography on silica gel (petroleum ether/CH_2Cl_2, 6:1) to yield carbazole **4c** (2.60 g, 88%) as a yellowish oil.

^{1}H NMR (400 MHz, 298 K, $CDCl_3$): δ = 8.12 (s, 1H, N-H), 7.74 (d, J = 1.5 Hz, 2H, H_{Ar}), 7.42 (d, J = 1.4 Hz, 2H, H_{Ar}), 2.75 (t, J = 7.8 Hz, 4H, H_{C4}), 1.72–1.65 (m, 4H, H_{C4}), 1.45–1.36 (m, 4H, H_{C4}), 0.97 (t, J = 7.4 Hz, 6H, H_{C4}) ppm.
^{13}C NMR (101 MHz, 298 K, $CDCl_3$): δ = 136.59, 136.23, 129.27, 124.96, 119.29, 104.09, 35.58, 34.38, 22.45, 14.13.
HR-ESI-MS: m/z = 458.00821 $[M+Na]^+$ ($C_{20}H_{23}NBr_2Na^+$ requires 458.00895).

1,8-dibromo-3,6-dipentyl-9*H*-carbazole (**5c**)

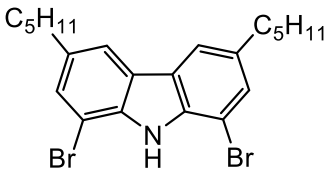

5c

Carbazole **5b** (2.30 g, 7.47 mmol, 1.0 eq.) was reacted with *N*-bromosuccinimide (2.79 g, 15.7 mmol, 2.1 eq.) and SiO_2 (7 g) in CH_2Cl_2 (250 mL) following general procedure C. The crude mixture was purified *via* column chromatography on silica gel (petroleum ether/CH_2Cl_2, 9:1) to yield carbazole **5c** (2.84 g, 82%) as a yellowish oil.

^{1}H NMR (400 MHz, 298 K, $CDCl_3$): δ = 8.13 (s, 1H, N-H), 7.75 (d, J = 1.5 Hz, 2H, H_{Ar}), 7.42 (d, J = 1.4 Hz, 2H, H_{Ar}), 2.74 (t, J = 7.9 Hz, 4H, H_{C5}), 1.70 (p, J = 7.6 Hz, 4H, H_{C5}), 1.39–1.34 (m, 8H, H_{C5}), 0.91 (t, J = 7.2 Hz, 6H, H_{C5}) ppm.
^{13}C NMR (101 MHz, 298 K, $CDCl_3$): δ = 136.62, 136.31, 129.28, 124.98, 119.32, 104.11, 35.89, 31.94, 31.60, 22.72, 14.20.
HR-ESI-MS: m/z = 462.04381 $[M-H]^-$ ($C_{22}H_{26}NBr_2^-$ requires 462.04375).

1,8-dibromo-3,6-dihexyl-9*H*-carbazole (**6c**)

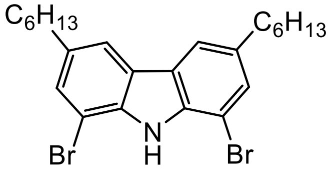

6c

Carbazole **6c** was synthesized following a previously reported procedure.

Analytical data for ^{1}H NMR were in agreement with those reported below that are found in literature[4].

^{1}H NMR (400 MHz, 298 K, $CDCl_3$): δ = 8.07 (s, 1H), 7.69 (s, 2H), 7.38 (s, 2H), 2.70 (t, J = 7.6 Hz, 4H), 1.68–1.64 (m, 4H), 1.35–1.30 (m, 12H), 0.88 (t, J = 6.8 Hz, 6H) ppm.

1,8-dibromo-3,6-diheptyl-9*H*-carbazole (**7c**)

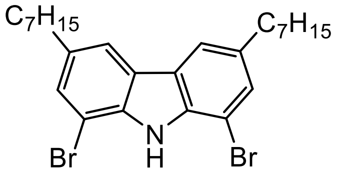

7c

Carbazole **7b** (2.17 g, 5.96 mmol, 1.0 eq.) was reacted with *N*-bromosuccinimide (2.23 g, 12.5 mmol, 2.1 eq.) and SiO_2 (6 g) in CH_2Cl_2 (200 mL) following general procedure C. The crude mixture was purified *via* column chromatography on silica gel (petroleum ether/CH_2Cl_2, 9:1) to yield carbazole **7c** (2.76 g, 89%) as an off-white solid.

^{1}H NMR (400 MHz, 298 K, $CDCl_3$): δ = 8.13 (s, 1H, N-H), 7.75 (d, J = 1.4 Hz, 2H, H_{Ar}), 7.42 (d, J = 1.4 Hz, 2H, H_{Ar}), 2.74 (t, J = 7.9 Hz, 4H, H_{C7}), 1.69 (p, J = 8.1 Hz, 4H, H_{C7}), 1.38–1.26 (m, 16H, H_{C7}), 0.89 (t, J = 7.0 Hz, 6H, H_{C7}) ppm.
^{13}C NMR (101 MHz, 298 K, $CDCl_3$): δ = 136.63, 136.34, 129.29, 125.00, 119.32, 104.11, 35.93, 32.27, 31.96, 29.36, 29.35, 22.82, 14.25.
HR-ESI-MS: m/z = 518.10653 $[M-H]^-$ ($C_{26}H_{34}NBr_2^-$ requires 518.10635).

1,8-dibromo-3,6-dioctyl-9*H*-carbazole (**8c**)

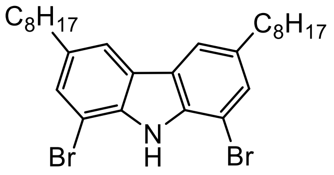

8c

Carbazole **8c** was synthesized following a previously reported procedure[1].

Analytical data were in agreement with those reported in literature.

1,8-dibromo-3,6-didecyl-9*H*-carbazole (**10c**)

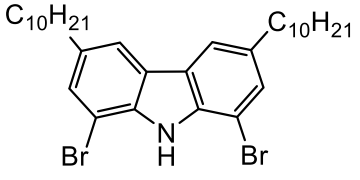

10c

Carbazole **10b** (2.47 g, 5.52 mmol, 1.0 eq.) was reacted with *N*-bromosuccinimide (2.06 g, 11.6 mmol, 2.1 eq.) and SiO_2 (5 g) in CH_2Cl_2 (200 mL) following general procedure C. The crude mixture was purified *via* column chromatography on silica gel (petroleum ether/CH_2Cl_2, 20:1) to yield carbazole **10c** (2.64 g, 79%) as a colorless oil.

^{1}H NMR (400 MHz, 298 K, $CDCl_3$): δ = 8.12 (s, 1H, N-H), 7.74 (d, J = 1.4 Hz, 2H, H_{Ar}), 7.42 (d, J = 1.4 Hz, 2H, H_{Ar}), 2.74 (t, J = 7.9 Hz, 4H, H_{C10}), 1.70 (q, J = 7.7 Hz, 4H, H_{C10}), 1.36–1.26 (m, 28H, H_{C10}), 0.90 (t, J = 6.8 Hz, 6H, H_{C10}) ppm.
^{13}C NMR (101 MHz, 298 K, $CDCl_3$): δ = 136.58, 136.26, 129.24, 124.96, 119.27, 104.09, 35.92, 32.25, 32.07, 29.79, 29.75, 29.69, 29.50, 29.43, 22.85, 14.28.
HR-ESI-MS: m/z = 602.20019 [M−H]$^-$ ($C_{32}H_{46}NBr_2^-$ requires 602.20025).

General procedure D: borylation of carbazole

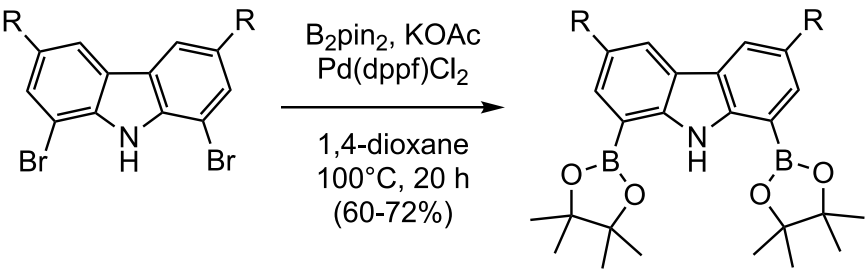

A stirring solution of the corresponding brominated carbazole (1.0 eq.), B_2pin_2 (2.4 eq.) and KOAc (8.0 eq.) in 1,4-dioxane was degassed in a heat-gun dried Schlenk flask by bubbling N_2 through over 15 minutes. $Pd(dppf)Cl_2$ (20 mol%) was added and the reaction mixture was heated to 100 °C for 20 h. The crude product was passed over a silica plug eluted with EtOAc and the solvent was removed *in vacuo*. Purification by column chromatography on silica gel (petroleum ether/CH_2Cl_2, 2:1) yielded the corresponding borylated carbazole.

3,6-dimethyl-1,8-bis(4,4,5,5-tetramethyl-1,3,2-dioxaborolan-2-yl)-9H-carbazole (**1d**)

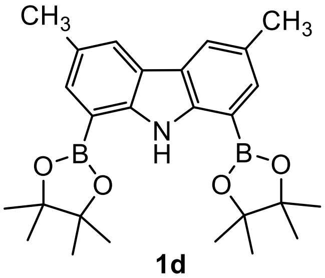

1d

Carbazole **1c** (1.50 g, 4.25 mmol, 1.0 eq.) was reacted with B_2pin_2 (2.59 g, 10.2 mmol, 2.4 eq.), KOAc (3.34 g, 34.0 mmol, 8.0 eq.) and $Pd(dppf)Cl_2$ (622 mg, 0.85 mmol, 20 mol%) in 1,4-dioxane (60 mL) following general procedure D to yield carbazole **1d** (1.37 g, 72%) as a white solid.

^{1}**H NMR** (400 MHz, 298 K, $CDCl_3$): δ = 9.97 (s, 1H, N-H), 7.95 (d, J = 1.8 Hz, 2H, H_{Ar}), 7.66 (d, J = 1.7 Hz, 2H, H_{Ar}), 2.52 (s, 3H, H_{C1}), 1.46 (s, 24H, H_{Bpin}) ppm.
^{13}**C NMR** (101 MHz, 298 K, $CDCl_3$): δ = 143.77, 133.61, 127.33, 123.88, 122.13, 83.78, 25.25, 21.38 (the aromatic C next to the B is not observed due to the relaxation time of B).
HR-ESI-MS: m/z = 448.28213 $[M+H]^+$ ($C_{26}H_{36}NO_4B_2^+$ requires 448.28250).

3,6-diethyl-1,8-bis(4,4,5,5-tetramethyl-1,3,2-dioxaborolan-2-yl)-9H-carbazole (**2d**)

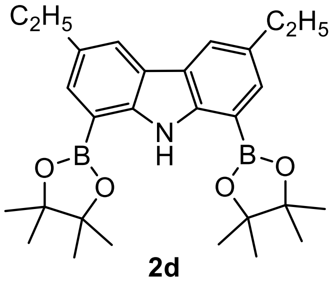

2d

Carbazole **2c** (2.21 g, 5.79 mmol, 1.0 eq.) was reacted with B_2pin_2 (3.53 g, 13.9 mmol, 2.4 eq.), KOAc (4.55 g, 46.3 mmol, 8.0 eq.) and Pd(dppf)Cl_2 (847 mg, 1.16 mmol, 20 mol%) in 1,4-dioxane (90 mL) following general procedure D to yield carbazole **2d** (1.66 g, 60%) as a white solid.

^{1}**H NMR** (400 MHz, 298 K, $CDCl_3$): δ = 10.02 (s, 1H, N-H), 8.01 (d, J = 1.7 Hz, 2H, H_{Ar}), 7.70 (d, J = 1.8 Hz, 2H, H_{Ar}), 2.84 (q, J = 7.6 Hz, 4H, H_{C2}), 1.47 (s, 24H, H_{Bpin}), 1.35 (t, J = 7.6 Hz, 6H, H_{C2}) ppm.
^{13}**C NMR** (101 MHz, 298 K, $CDCl_3$): δ = 144.05, 134.16, 132.70, 122.71, 122.23, 83.80, 29.06, 25.31, 16.77 (the aromatic C next to the B is not observed due to the relaxation time of B).
HR-ESI-MS: m/z = 476.31353 $[M+H]^+$ ($C_{28}H_{40}NO_4B_2^+$ requires 476.31380).

3,6-dipropyl-1,8-bis(4,4,5,5-tetramethyl-1,3,2-dioxaborolan-2-yl)-9H-carbazole (**3d**)

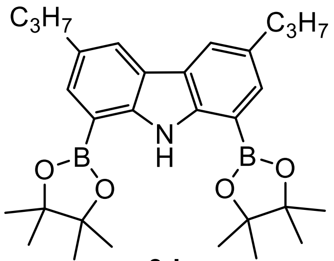

3d

Carbazole **3c** (2.41 g, 5.90 mmol, 1.0 eq.) was reacted with B_2pin_2 (3.60 g, 14.2 mmol, 2.4 eq.), KOAc (4.63 g, 47.2 mmol, 8.0 eq.) and Pd(dppf)Cl_2 (863 mg, 1.18 mmol, 20 mol%) in 1,4-dioxane (90 mL) following general procedure D to yield carbazole **3d** (1.91 g, 64%) as a yellow solid.

^{1}**H NMR** (400 MHz, 298 K, $CDCl_3$): δ = 10.00 (s, 1H, N-H), 7.97 (d, J = 1.8 Hz, 2H, H_{Ar}), 7.65 (d, J = 1.7 Hz, 2H, H_{Ar}), 2.75 (t, J = 7.7 Hz, 4H, H_{C3}), 1.79–1.69 (m, 4H, H_{C3}), 1.46 (s, 24H, H_{Bpin}), 0.97 (t, J = 7.3 Hz, 6H, H_{C3}) ppm.
^{13}**C NMR** (101 MHz, 298 K, $CDCl_3$): δ = 144.05, 133.22, 132.50, 123.38, 122.14, 83.77, 38.25, 25.61, 25.29, 14.07 (the aromatic C next to the B is not observed due to the relaxation time of B).
HR-ESI-MS: m/z = 504.34466 $[M+H]^+$ ($C_{30}H_{44}NO_4B_2^+$ requires 504.34510).

3,6-dibutyl-1,8-bis(4,4,5,5-tetramethyl-1,3,2-dioxaborolan-2-yl)-9H-carbazole (**4d**)

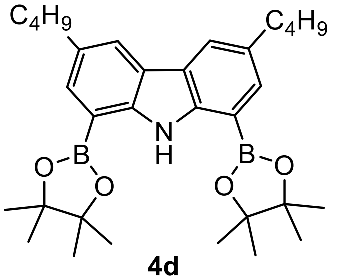

4d

Carbazole **4c** (1.98 g, 4.53 mmol, 1.0 eq.) was reacted with B_2pin_2 (2.76 g, 10.9 mmol, 2.4 eq.), KOAc (3.56 g, 36.2 mmol, 8.0 eq.) and Pd(dppf)Cl_2 (663 mg, 0.91 mmol, 20 mol%) in 1,4-dioxane (70 mL) following general procedure D to yield carbazole **4d** (1.53 g, 64%) as a white solid.

^{1}H NMR (400 MHz, 298 K, $CDCl_3$): δ = 10.06 (s, 1H, N-H), 8.02 (d, J = 1.1 Hz, 2H, H_{Ar}), 7.71 (d, J = 1.7 Hz, 2H, H_{Ar}), 2.83 (t, J = 7.8 Hz, 4H, H_{C4}), 1.75 (p, J = 8.0 Hz, 4H, H_{C4}), 1.49 (s, 24H, H_{Bpin}), 1.49–1.42 (m, 4H, H_{C4}), 1.00 (t, J = 7.4 Hz, 6H, H_{C4}) ppm.
^{13}C NMR (101 MHz, 298 K, $CDCl_3$): δ = 144.01, 133.14, 132.71, 123.28, 122.13, 83.75, 35.86, 34.81, 25.27, 22.65, 14.20 (the aromatic C next to the B is not observed due to the relaxation time of B).
HR-ESI-MS: m/z = 532.37702 $[M+H]^+$ ($C_{32}H_{48}NO_4B_2^+$ requires 532.37640).

3,6-dipentyl-1,8-bis(4,4,5,5-tetramethyl-1,3,2-dioxaborolan-2-yl)-9H-carbazole (**5d**)

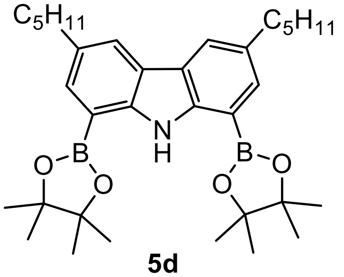

5d

Carbazole **5c** (2.47 g, 5.30 mmol, 1.0 eq.) was reacted with B_2pin_2 (3.23 g, 12.7 mmol, 2.4 eq.), KOAc (4.16 g, 42.4 mmol, 8.0 eq.) and Pd(dppf)Cl_2 (776 mg, 1.06 mmol, 20 mol%) in 1,4-dioxane (75 mL) following general procedure D to yield carbazole **5d** (1.86 g, 63%) as an off-white solid.

^{1}H NMR (400 MHz, 298 K, $CDCl_3$): δ = 10.00 (s, 1H, N-H), 7.97 (d, J = 1.7 Hz, 2H, H_{Ar}), 7.65 (d, J = 1.7 Hz, 2H, H_{Ar}), 2.74 (t, J = 7.9 Hz, 4H, H_{C5}), 1.71 (p, J = 7.8 Hz, 4H, H_{C5}), 1.46 (s, 24H, H_{Bpin}), 1.38–1.33 (m, 8H, H_{C5}), 0.90 (t, J = 7.2 Hz, 6H, H_{C5}) ppm.
^{13}C NMR (101 MHz, 298 K, $CDCl_3$): δ = 144.02, 133.13, 132.80, 123.30, 122.15, 83.79, 36.18, 32.36, 31.88, 25.32, 22.79, 14.26 (the aromatic C next to the B is not observed due to the relaxation time of B).
HR-ESI-MS: m/z = 560.40806 $[M+H]^+$ ($C_{34}H_{52}NO_4B_2^+$ requires 560.40770).

3,6-dihexyl-1,8-bis(4,4,5,5-tetramethyl-1,3,2-dioxaborolan-2-yl)-9H-carbazole (**6d**)

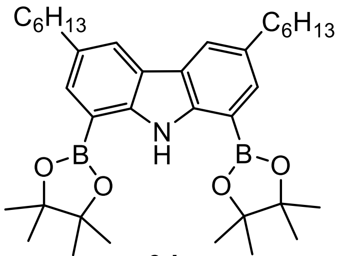

6d

Carbazole **6d** was synthesized following a previously reported procedure[1].

Analytical data were in agreement with those reported in literature.

3,6-diheptyl-1,8-bis(4,4,5,5-tetramethyl-1,3,2-dioxaborolan-2-yl)-9H-carbazole (**7d**)

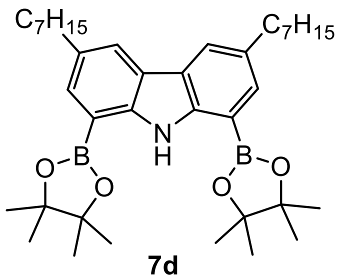

7d

Carbazole **7c** (2.11 g, 4.05 mmol, 1.0 eq.) was reacted with B_2pin_2 (2.47 g, 9.72 mmol, 2.4 eq.), KOAc (3.18 g, 32.4 mmol, 8.0 eq.) and Pd(dppf)Cl_2 (593 mg, 0.81 mmol, 20 mol%) in 1,4-dioxane (60 mL) following general procedure D to yield carbazole **7d** (1.59 g, 64%) as a yellow solid.

^{1}H NMR (400 MHz, 298 K, $CDCl_3$): δ = 10.04 (s, 1H, N-H), 8.01 (d, J = 1.7 Hz, 2H, H_{Ar}), 7.70 (d, J = 1.8 Hz, 2H, H_{Ar}), 2.81 (t, J = 8.5 Hz, 4H, H_{C7}), 1.75 (p, J = 7.0 Hz, 4H, H_{C7}), 1.49 (s, 24H, H_{Bpin}), 1.42–1.31 (m, 16H, H_{C7}), 0.92 (t, J = 6.8 Hz, 6H, H_{C7}) ppm.
^{13}C NMR (101 MHz, 298 K, $CDCl_3$): δ = 144.02, 133.14, 132.78, 123.29, 122.16, 83.76, 36.21, 32.68, 32.01, 29.42, 25.29, 22.83, 14.26 (the aromatic C next to the B is not observed due to the relaxation time of B).
HR-ESI-MS: m/z = 616.47007 $[M+H]^+$ ($C_{38}H_{60}NO_4B_2^+$ requires 616.47030).

3,6-dioctyl-1,8-bis(4,4,5,5-tetramethyl-1,3,2-dioxaborolan-2-yl)-9H-carbazole (**8d**)

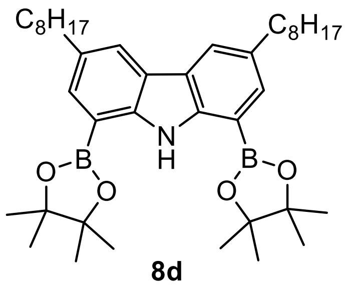

8d

Carbazole **8d** was synthesized following a previously reported procedure[1].

Analytical data were in agreement with those reported in literature.

3,6-didecyl-1,8-bis(4,4,5,5-tetramethyl-1,3,2-dioxaborolan-2-yl)-9H-carbazole (**10d**)

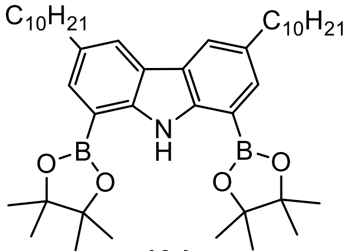

10d

Carbazole **10c** (2.54 g, 4.19 mmol, 1.0 eq.) was reacted with B_2pin_2 (2.55 g, 10.1 mmol, 2.4 eq.), KOAc (3.29 g, 33.5 mmol, 8.0 eq.) and $Pd(dppf)Cl_2$ (613 mg, 0.84 mmol, 20 mol%) in 1,4-dioxane (60 mL) following general procedure D to yield carbazole **10d** (1.88 g, 64%) as a yellow solid.

^{1}H NMR (400 MHz, 298 K, $CDCl_3$): δ = 10.01 (s, 1H, N-H), 7.98 (d, J = 1.7 Hz, 2H, H_{Ar}), 7.66 (d, J = 1.7 Hz, 2H, H_{Ar}), 2.78 (t, J = 7.8 Hz, 4H, H_{C10}), 1.72 (q, J = 7.6 Hz, 4H, H_{C10}), 1.47 (s, 24H, H_{Bpin}), 1.37–1.25 (m, 28H, H_{C10}), 0.89 (t, J = 7.1 Hz, 6H, H_{C10}) ppm.
^{13}C NMR (101 MHz, 298 K, $CDCl_3$): δ = 144.02, 133.13, 132.80, 123.30, 122.15, 83.78, 36.22, 32.68, 32.07, 29.80, 29.76, 29.66, 29.50, 25.31, 22.84, 14.27 (the aromatic C next to the B is not observed due to the relaxation time of B).
HR-ESI-MS: *m/z* = 700.56377 $[M+H]^+$ ($C_{44}H_{72}NO_4B_2^+$ requires 700.56420).

General procedure E: macrocyclization

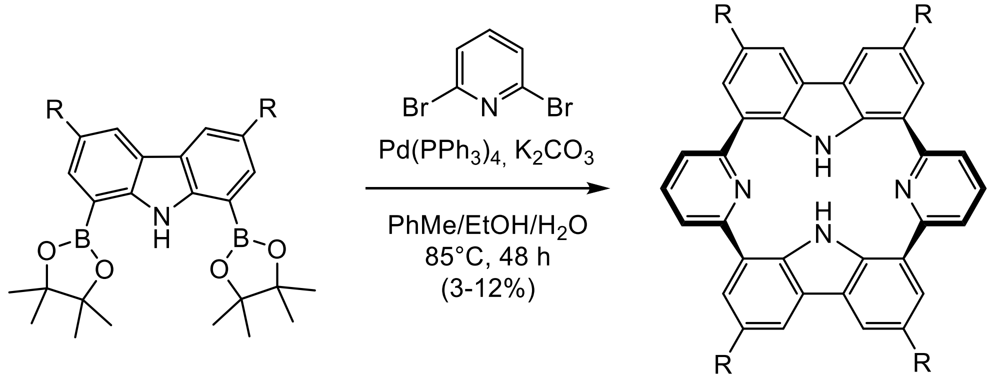

A heat gun-dried 1 L round bottom flask was charged with the corresponding borylated carbazole (0.50 mmol, 1.0 eq.), 2,6-dibromopyridine (118 mg, 0.50 mmol, 1.0 eq.) and K_2CO_3 (553 mg, 4.00 mmol, 8.0 eq.) in toluene (500 mL), ethanol (200 mL) and water (75 mL). N_2 was bubbled through the biphasic mixture for 15 minutes and $Pd(PPh_3)_4$ (17.3 mg, 15.0 µmol, 2 mol%) was added. The flask was placed in a pre-heated oil bath and stirred at 85 °C for 48 h. The solvent was removed *in vacuo* and the crude product was dissolved in CH_2Cl_2 (100 mL) and washed with brine (3×50 mL). The organic layer was dried over Na_2SO_4 and the solvent was removed under pressure. The crude mixture was subjected to column chromatography on silica (petroleum ether/CH_2Cl_2, 2:1 to 1:1) before performing recycling gel permeation chromatography ($CHCl_3$). This yielded the corresponding carpyridine.

<u>**2H-Car-C1**</u>

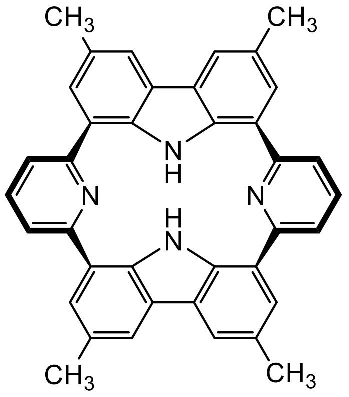

2H-Car-C1

Carbazole **1d** (224 mg, 0.50 mmol, 1.0 eq.) was reacted with 2,6-dibromopyridine following general procedure E to yield **2H-Car-C1** (4.5 mg, 3%) as a white solid.

^{1}H NMR (500 MHz, 298 K, $CDCl_3$): δ = 9.68 (s, 1H, N-H), 7.98–7.92 (m, 6H, H_{Ar}), 7.63 (d, J = 7.6 Hz, 4H, H_{Ar}), 7.35 (s, 4H, H_{Ar}), 2.59 (s, 12H, H_{C1}) ppm.
HR-ESI-MS: m/z = 541.23861 $[M+H]^+$ ($C_{38}H_{29}N_4^+$ requires 541.23867).

<u>**2H-Car-C2**</u>

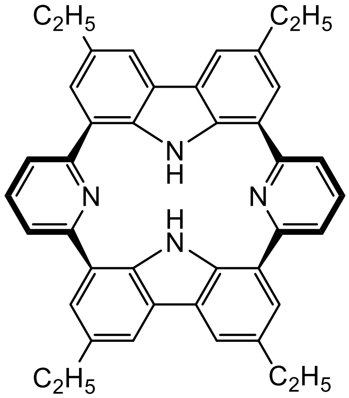

2H-Car-C2

Carbazole **2d** (238 mg, 0.50 mmol, 1.0 eq.) was reacted with 2,6-dibromopyridine following general procedure E to yield **2H-Car-C2** (10.4 mg, 7%) as a yellow solid.

^{1}H NMR (400 MHz, 298 K, $CDCl_3$): δ = 9.69 (s, 2H, N-H), 7.99 (d, J = 0.9 Hz, 4H, H_{Ar}), 7.96 (t, J = 7.8 Hz, 2H, H_{Ar}), 7.65 (d, J = 7.8 Hz, 4H, H_{Ar}), 7.37 (d, J = 1.7 Hz, 4H, H_{Ar}), 2.89 (q, J = 7.6 Hz, 8H, H_{C2}), 1.38 (t, J = 7.6 Hz, 12H, H_{C2}) ppm.
^{13}C NMR (101 MHz, 298 K, $CDCl_3$): δ = 159.50, 138.34, 136.33, 135.89, 128.02, 124.86, 124.22, 122.30, 119.59, 29.09, 16.65.
HR-ESI-MS: m/z = 597.30173 $[M+H]^+$ ($C_{42}H_{37}N_4^+$ requires 597.30127).

__2H-Car-C3__

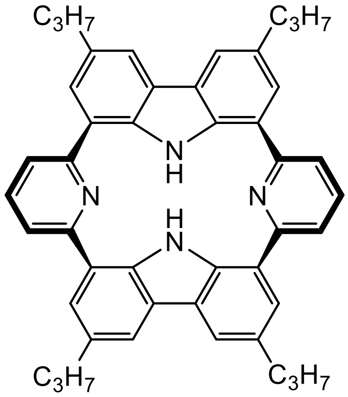

2H-Car-C3

Carbazole **3d** (252 mg, 0.50 mmol, 1.0 eq.) was reacted with 2,6-dibromopyridine following general procedure E to yield **2H-Car-C3** (20.0 mg, 12%) as a yellow solid.

^{1}H NMR (400 MHz, 298 K, $CDCl_3$): δ = 9.68 (s, 2H, N-H), 7.97 (d, J = 1.7 Hz, 4H, H_{Ar}), 7.94 (t, J = 7.8 Hz, 2H, H_{Ar}), 7.62 (d, J = 7.9 Hz, 4H, H_{Ar}), 7.34 (d, J = 1.6 Hz, 4H, H_{Ar}), 2.83 (t, J = 7.5 Hz, 8H, H_{C3}), 1.83–1.74 (m, 8H, H_{C3}), 1.00 (t, J = 7.3 Hz, 12H, H_{C3}) ppm.
^{13}C NMR (101 MHz, 298 K, $CDCl_3$): δ = 159.51, 138.33, 136.32, 134.22, 128.56, 124.81, 124.08, 122.29, 120.31, 38.22, 25.44, 14.00.
HR-ESI-MS: m/z = 653.36421 $[M+H]^+$ ($C_{46}H_{45}N_4^+$ requires 653.36387).

__2H-Car-C4__

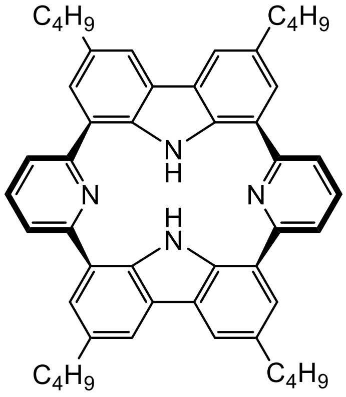

2H-Car-C4

Carbazole **4d** (266 mg, 0.50 mmol, 1.0 eq.) was reacted with 2,6-dibromopyridine following general procedure E to yield **2H-Car-C4** (18.0 mg, 10%) as a yellow solid.

^{1}H NMR (400 MHz, 298 K, $CDCl_3$): δ = 9.70 (s, 2H, N-H), 7.96 (s (br), 4H, H_{Ar}), 7.94 (t, J = 7.8 Hz, 2H, H_{Ar}), 7.63 (d, J = 7.8 Hz, 4H, H_{Ar}), 7.34 (s (br), 4H, H_{Ar}), 2.85 (t, J = 7.7 Hz, 8H, H_{C4}), 1.73 (p, J = 7.6 Hz, 8H, H_{C4}), 1.47–1.37 (m, 8H, H_{C4}), 0.96 (t, J = 7.3 Hz, 12H, H_{C4}) ppm.
^{13}C NMR (101 MHz, 298 K, $CDCl_3$): δ = 159.50, 138.31, 136.31, 134.43, 128.50, 124.80, 124.12, 122.27, 120.23, 35.83, 34.59, 22.50, 14.20.
HR-ESI-MS: m/z = 709.42612 $[M+H]^+$ ($C_{50}H_{53}N_4^+$ requires 709.42647).

__2H-Car-C5__

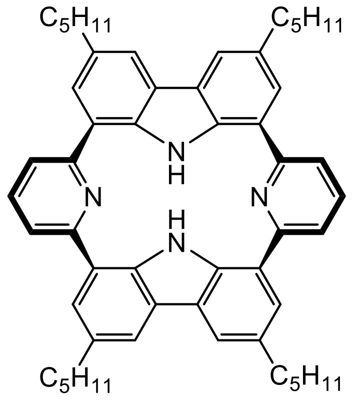

2H-Car-C5

Carbazole **5d** (280 mg, 0.50 mmol, 1.0 eq.) was reacted with 2,6-dibromopyridine following general procedure E to yield **2H-Car-C5** (14.8 mg, 8%) as a white solid.

^{1}H NMR (400 MHz, 298 K, $CDCl_3$): δ = 9.66 (s, 2H, N-H), 7.97 (d, J = 1.6 Hz, 4H, H_{Ar}), 7.93 (t, J = 7.8 Hz, 2H, H_{Ar}), 7.61 (d, J = 7.8 Hz, 4H, H_{Ar}), 7.34 (d, J = 1.6 Hz, 4H, H_{Ar}), 2.85 (t, J = 7.6 Hz, 8H, H_{C5}), 1.77 (p, J = 7.7 Hz, 8H, H_{C5}), 1.44–1.35 (m, 16H, H_{C5}), 0.93 (t, J = 7.1 Hz, 12H, H_{C5}) ppm.
^{13}C NMR (101 MHz, 298 K, $CDCl_3$): δ = 159.49, 138.30, 136.28, 134.47, 128.50, 124.80, 124.08, 122.26, 120.22, 36.12, 32.11, 31.67, 22.79, 14.24.
HR-ESI-MS: m/z = 765.48882 $[M+H]^+$ ($C_{54}H_{61}N_4^+$ requires 765.48907).

__2H-Car-C6__

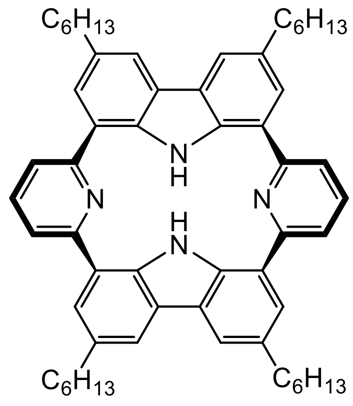

2H-Car-C6

2H-Car-C6 was synthesized following a previously reported procedure[1].

Analytical data were in agreement with those reported in literature.

^{1}H NMR (400 MHz, 298 K, $CDCl_3$): δ = 9.67 (s, 2H, N-H), 7.98 (d, J = 1.6 Hz, 4H, H_{Ar}), 7.93 (t, J = 7.8 Hz, 2H, H_{Ar}), 7.61 (d, J = 7.8 Hz, 4H, H_{Ar}), 7.34 (d, J = 1.6 Hz, 4H, H_{Ar}), 2.85 (t, J = 7.7 Hz, 8H, H_{C6}), 1.80–1.72 (m, 8H, H_{C6}), 1.44–1.33 (m, 24H, H_{C6}), 0.92 (t, J = 7.1 Hz, 12H, H_{C6}) ppm.
^{13}C NMR (101 MHz, 298 K, $CDCl_3$): δ = 159.48, 138.29, 136.27, 134.46, 128.48, 124.80, 124.07, 122.25, 120.21, 36.16, 32.40, 31.99, 29.17, 22.80, 14.29 ppm.
HR-ESI-MS: m/z = 821.55022 $[M+H]^+$ ($C_{58}H_{69}N_4^+$ requires 821.55167).

2H-Car-C7

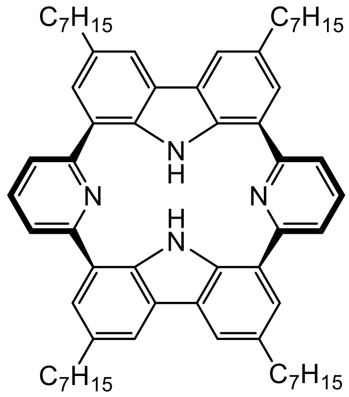

2H-Car-C7

Carbazole **7d** (308 mg, 0.50 mmol, 1.0 eq.) was reacted with 2,6-dibromopyridine following general procedure E to yield **2H-Car-C7** (14.0 mg, 6%) as a white solid.

^{1}H NMR (400 MHz, 298 K, $CDCl_3$): δ = 9.67 (s, 2H, N-H), 7.96 (d, J = 1.7 Hz, 4H, H_{Ar}), 7.93 (t, J = 7.8 Hz, 2H, H_{Ar}), 7.62 (d, J = 7.8 Hz, 4H, H_{Ar}), 7.34 (d, J = 1.6 Hz, 4H, H_{Ar}), 2.84 (t, J = 7.7 Hz, 8H, H_{C7}), 1.76 (p, J = 7.6 Hz, 8H, H_{C7}), 1.42–1.26 (m, 32H, H_{C7}), 0.89 (t, J = 6.9 Hz, 12H, H_{C7}) ppm.
^{13}C NMR (101 MHz, 298 K, $CDCl_3$): δ = 159.51, 138.32, 136.29, 134.50, 128.49, 124.82, 124.09, 122.28, 120.23, 36.17, 32.45, 32.01, 29.46, 29.43, 22.85, 14.26.
HR-ESI-MS: m/z = 877.61441 $[M+H]^+$ ($C_{62}H_{77}N_4^+$ requires 877.61427).

2H-Car-C8

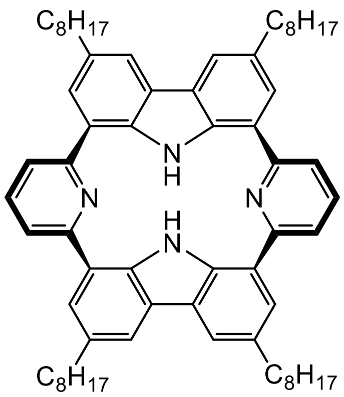

2H-Car-C8

2H-Car-C8 was synthesized following a previously reported procedure[1].

Analytical data were in agreement with those reported in literature.

^{1}H NMR (400 MHz, 298 K, $CDCl_3$): δ = 9.67 (s, 2H, N-H), 7.97 (s, 4H, H_{Ar}), 7.92 (t, J = 7.8 Hz, 2H, H_{Ar}), 7.60 (d, J = 7.8 Hz, 4H, H_{Ar}), 7.34 (s, 4H, H_{Ar}), 2.85 (t, J = 7.6 Hz, 8H, H_{C8}), 1.77 (p, J = 7.3 Hz, 8H, H_{C8}), 1.46–1.26 (m, 40H, H_{C8}), 0.90 (t, J = 6.5 Hz, 12H, H_{C8}) ppm.
^{13}C NMR (101 MHz, 298 K, $CDCl_3$): δ = 159.50, 138.27, 136.28, 134.47, 128.49, 124.81, 124.08, 122.25, 120.22, 36.16, 32.44, 32.08, 29.73, 29.50, 29.46, 22.83, 14.27 ppm.
HR-ESI-MS: m/z = 933.67593 $[M+H]^+$ ($C_{66}H_{85}N_4^+$ requires 933.67688).

__2H-Car-C10__

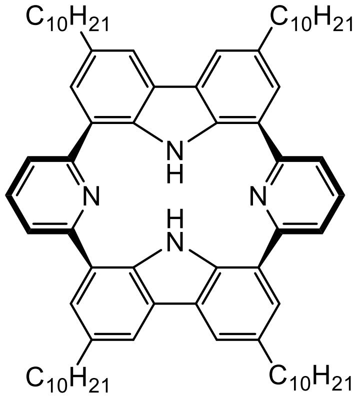

2H-Car-C10

Carbazole **10d** (350 mg, 0.50 mmol, 1.0 eq.) was reacted with 2,6-dibromopyridine following general procedure E to yield **2H-Car-C10** (7.50 mg, 3%) as a white solid.

^{1}H NMR (400 MHz, 298 K, $CDCl_3$): δ = 9.68 (s, 2H, N-H), 7.96 (d, J = 1.6 Hz, 4H, H_{Ar}), 7.95 (t, J = 7.8 Hz, 2H, H_{Ar}), 7.63 (d, J = 7.8 Hz, 4H, H_{Ar}), 7.34 (d, J = 1.6 Hz, 4H, H_{Ar}), 2.84 (t, J = 7.6 Hz, 8H, H_{C10}), 1.75 (p, J = 7.8 Hz, 8H, H_{C10}), 1.40–1.23 (m, 8H, H_{C10}), 0.88 (t, J = 7.1 Hz, 12H, H_{C10}) ppm.
^{13}C NMR (101 MHz, 298 K, $CDCl_3$): δ = 159.52, 138.34, 136.30, 134.52, 128.49, 124.83, 124.10, 122.30, 120.24, 36.17, 32.45, 32.06, 29.81, 29.79, 29.77, 29.49, 22.84, 14.27 (1 aliphatic C is not showing due to overlapping of signals).
HR-ESI-MS: m/z = 1045.80207 $[M+H]^+$ ($C_{74}H_{101}N_4^+$ requires 1045.80208).

NMR Characterization

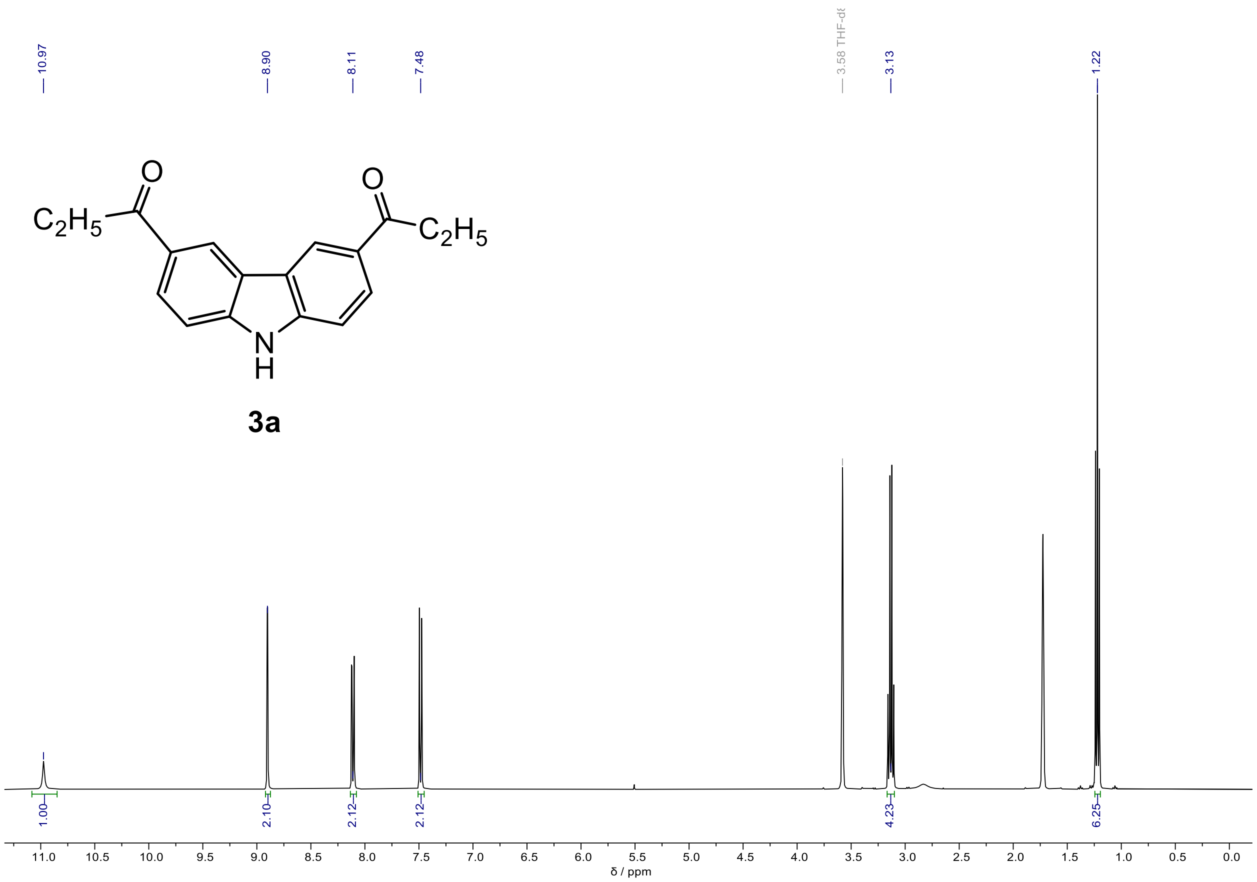

Supplementary Figure 1. ^{1}H NMR of **3a** (400 MHz, 298 K, THF-d_8).

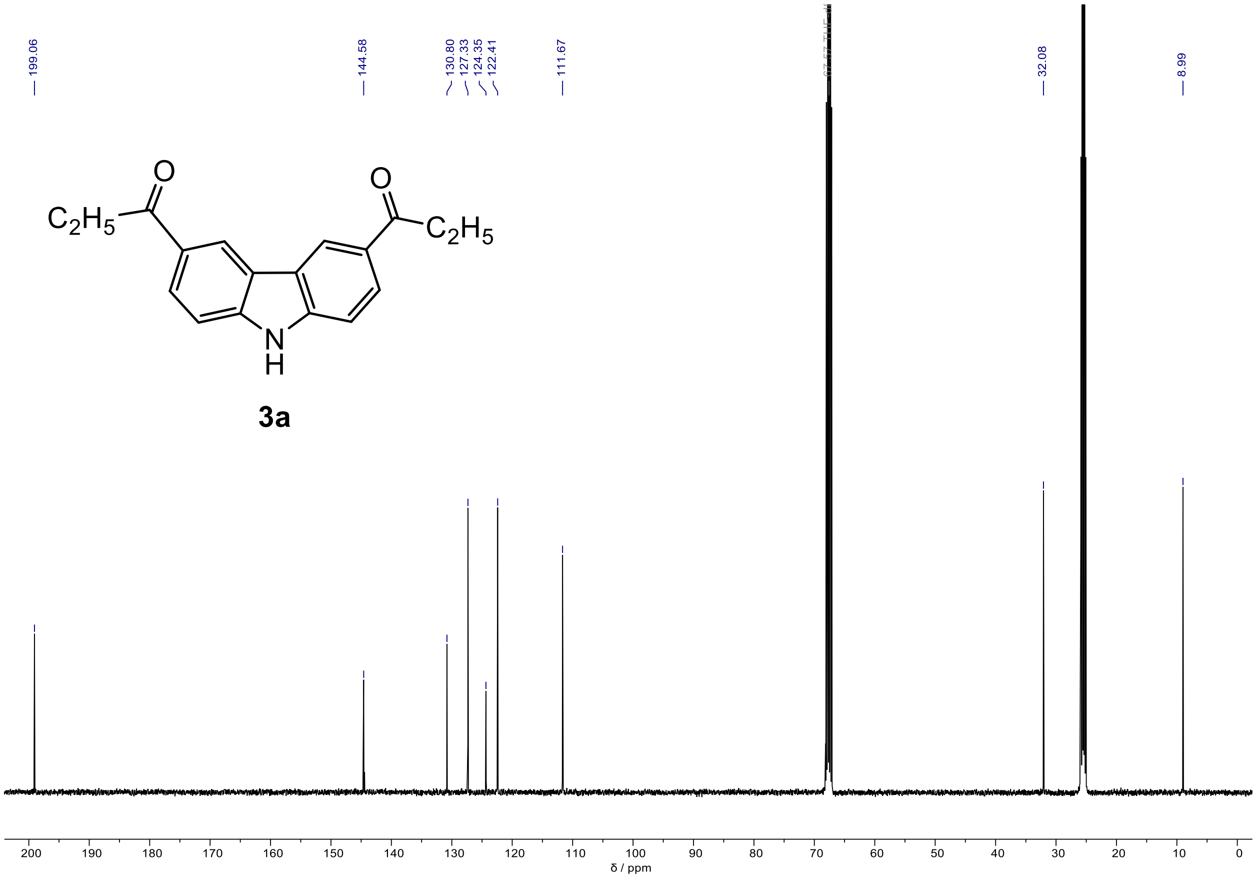

Supplementary Figure 2. ^{13}C NMR of **3a** (101 MHz, 298 K, THF-d_8).

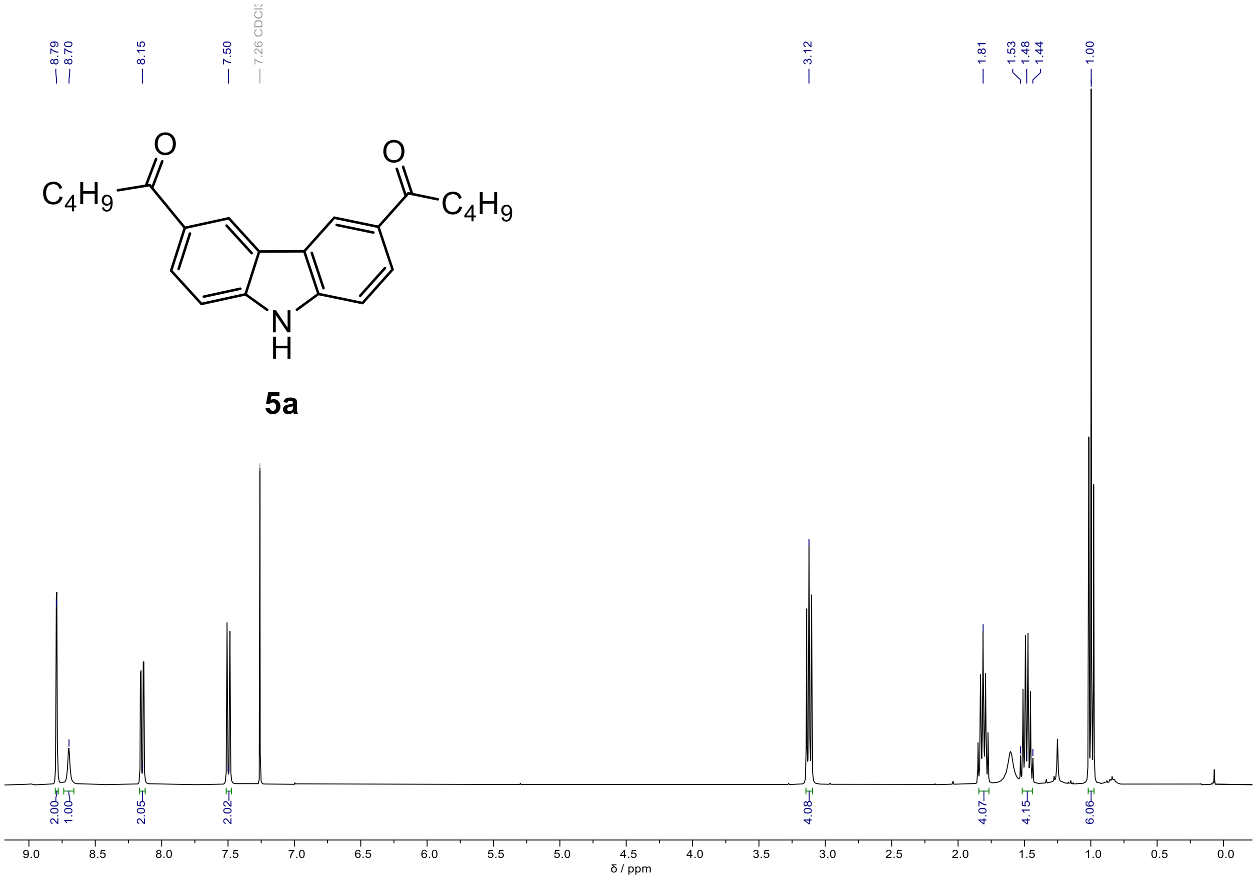

Supplementary Figure 3. ^{1}H NMR of **5a** (400 MHz, 298 K, $CDCl_3$).

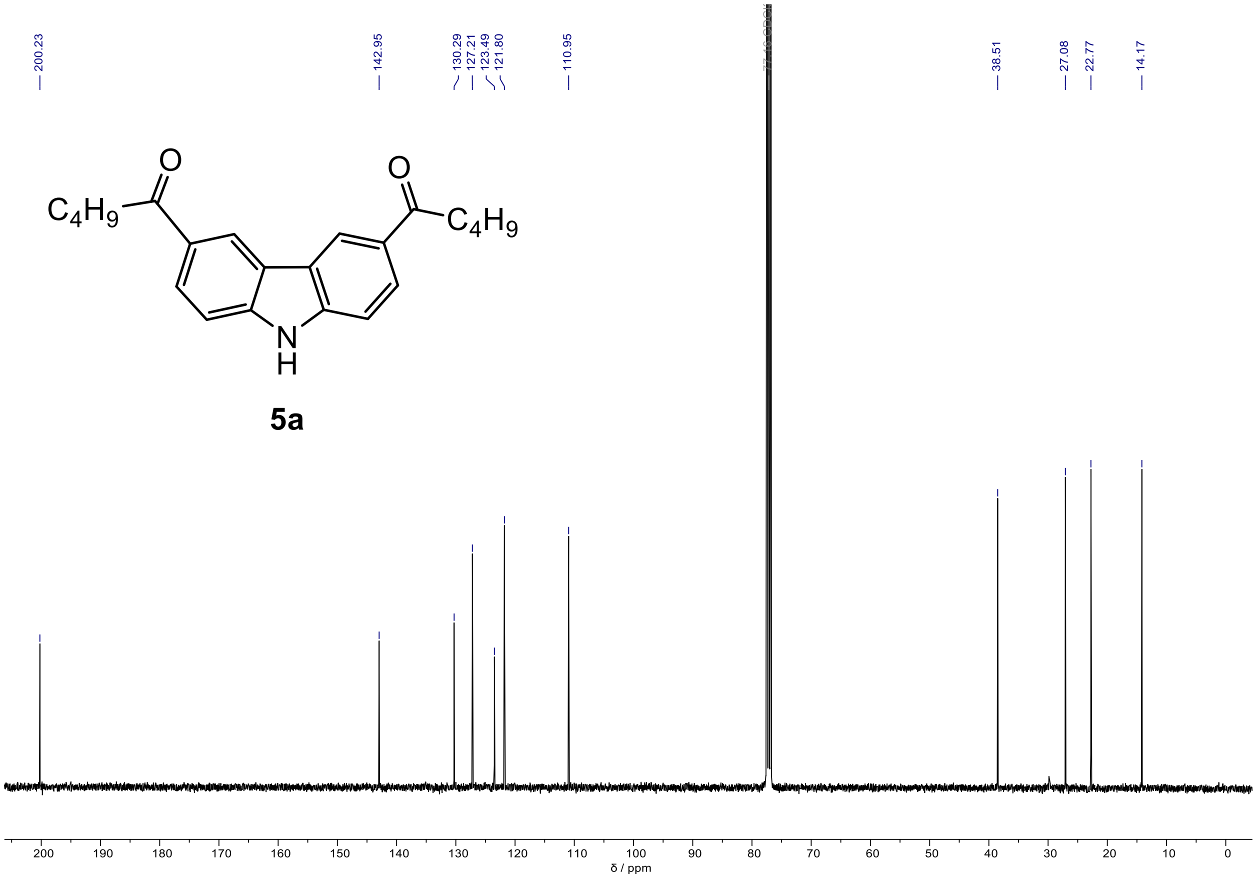

Supplementary Figure 4. ^{13}C NMR of **5a** (101 MHz, 298 K, $CDCl_3$).

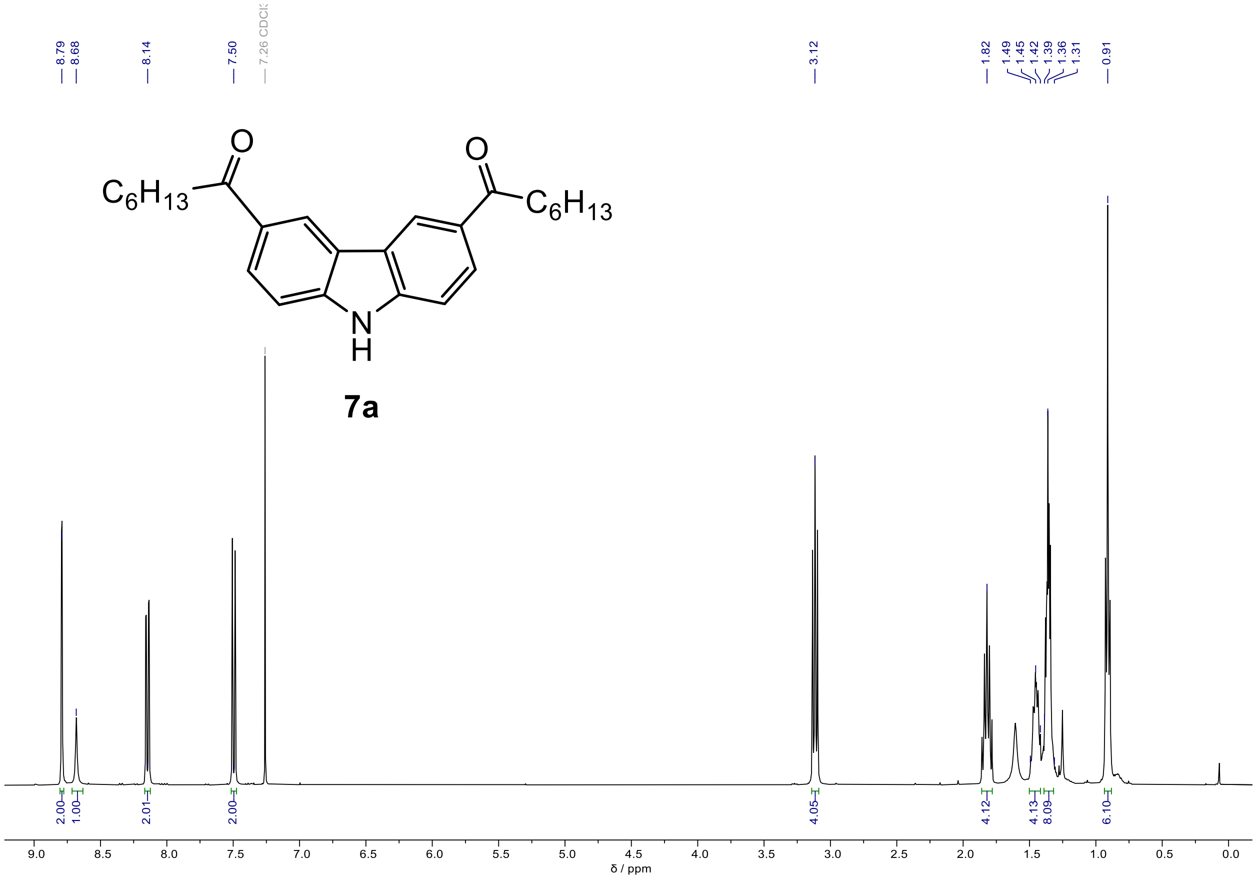

Supplementary Figure 5. ^{1}H NMR of **7a** (400 MHz, 298 K, $CDCl_3$).

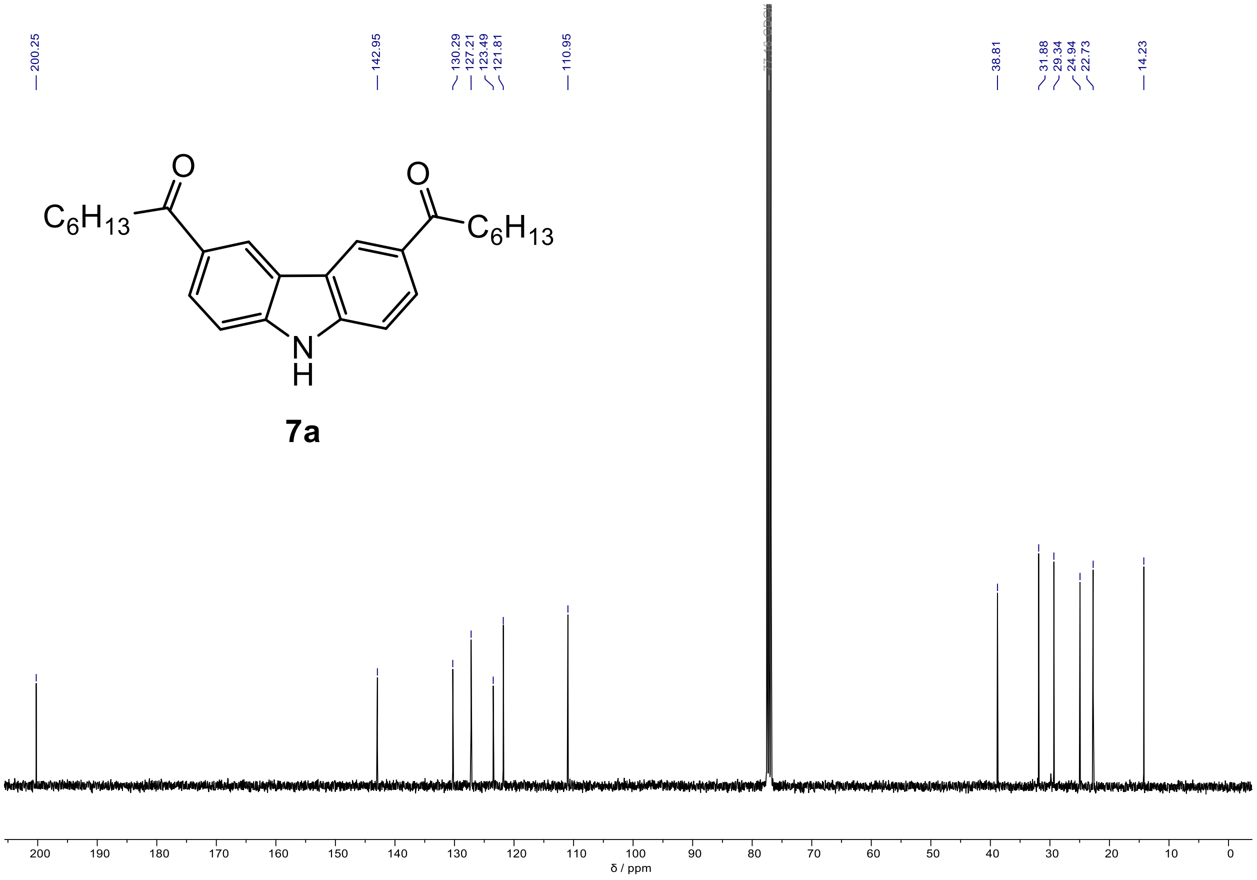

Supplementary Figure 6. ^{13}C NMR of **7a** (101 MHz, 298 K, $CDCl_3$).

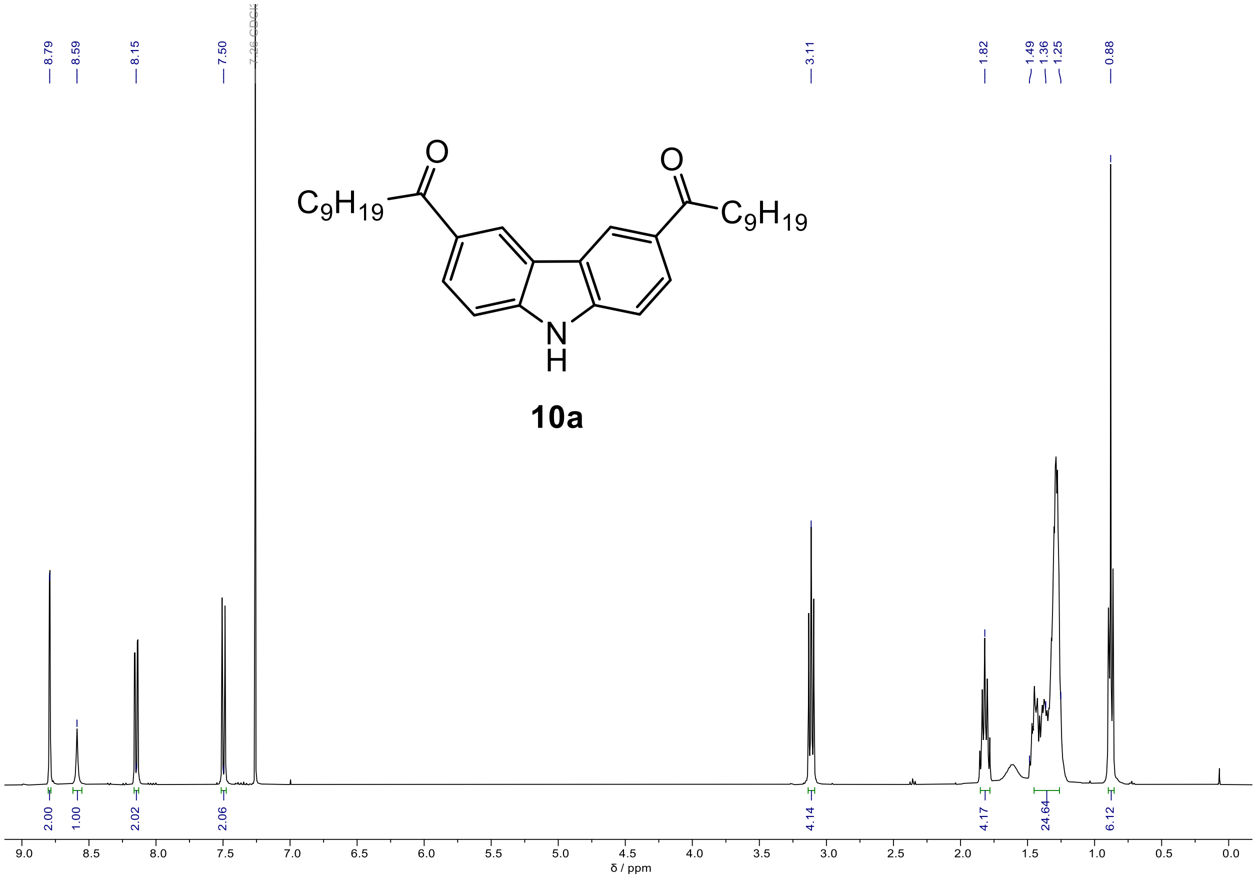

Supplementary Figure 7. ^{1}H NMR of **10a** (400 MHz, 298 K, $CDCl_3$).

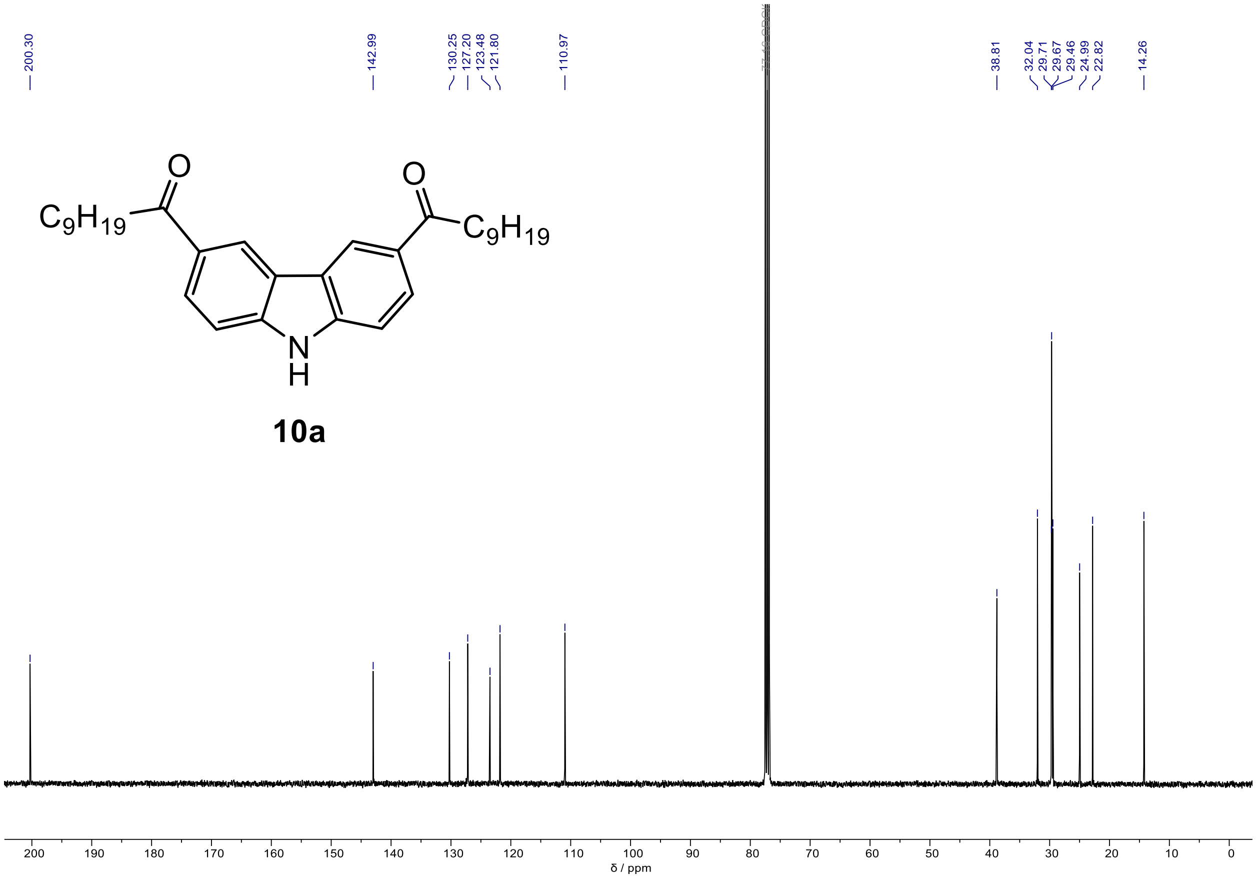

Supplementary Figure 8. ^{13}C NMR of **10a** (101 MHz, 298 K, $CDCl_3$).

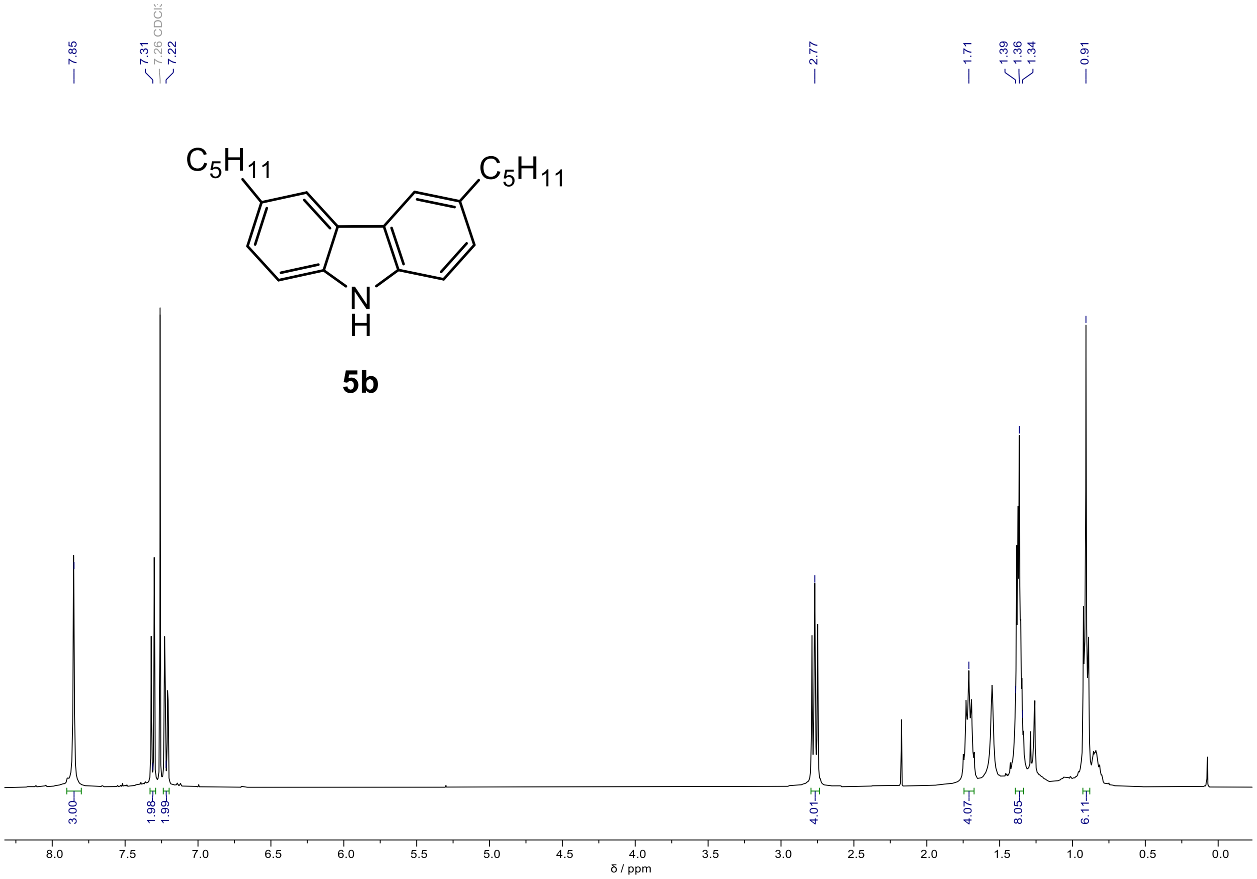

Supplementary Figure 9. ^{1}H NMR of **5b** (400 MHz, 298 K, $CDCl_3$).

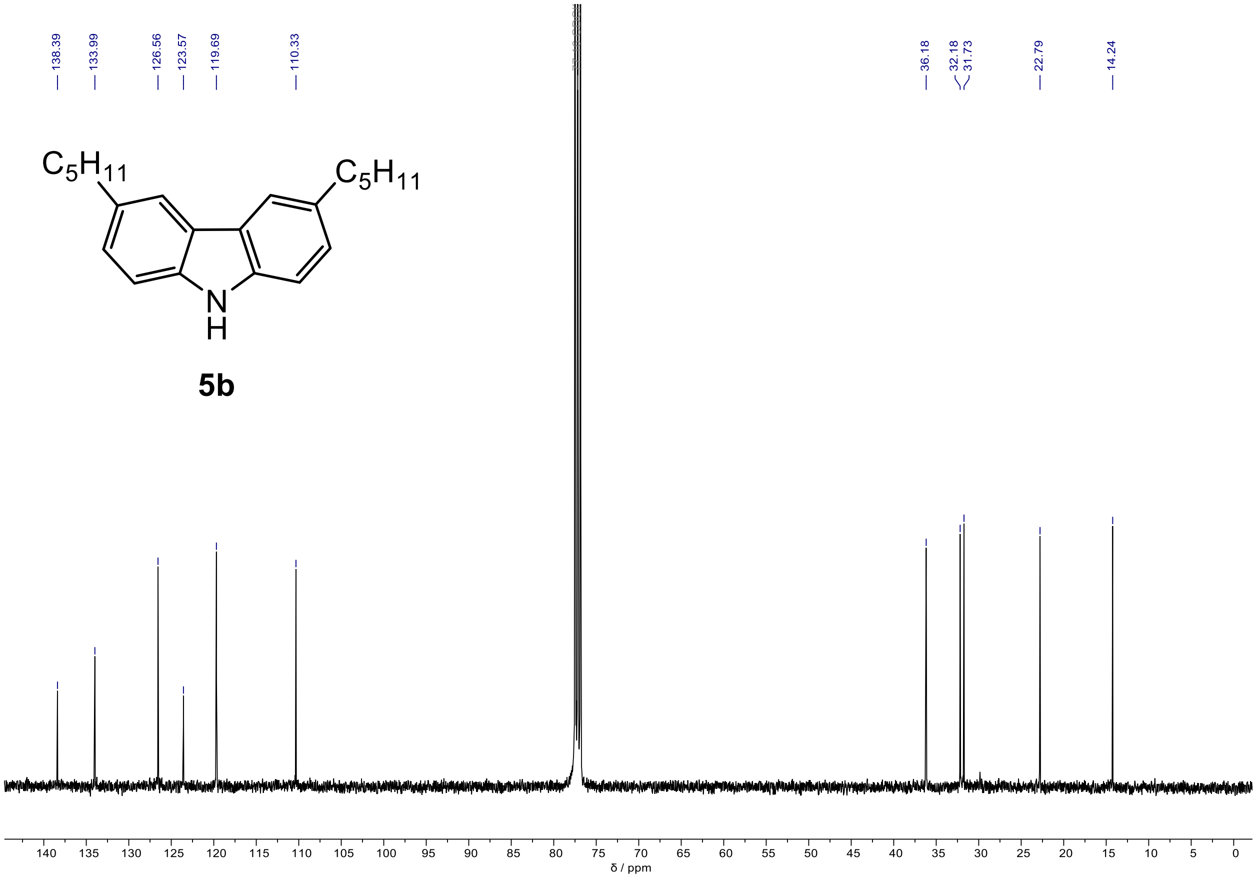

Supplementary Figure 10. ^{13}C NMR of **5b** (101 MHz, 298 K, $CDCl_3$).

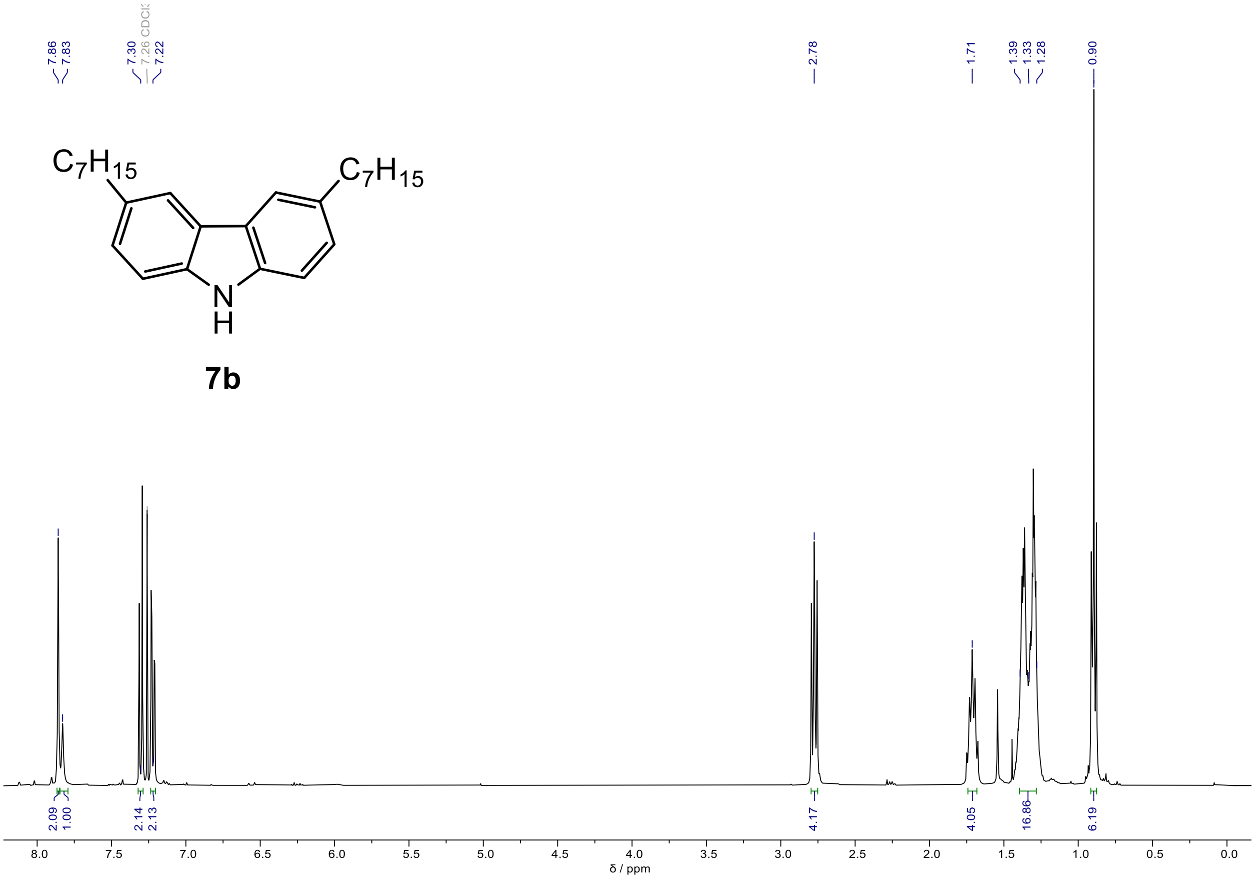

Supplementary Figure 11. ^{1}H NMR of **7b** (400 MHz, 298 K, $CDCl_3$).

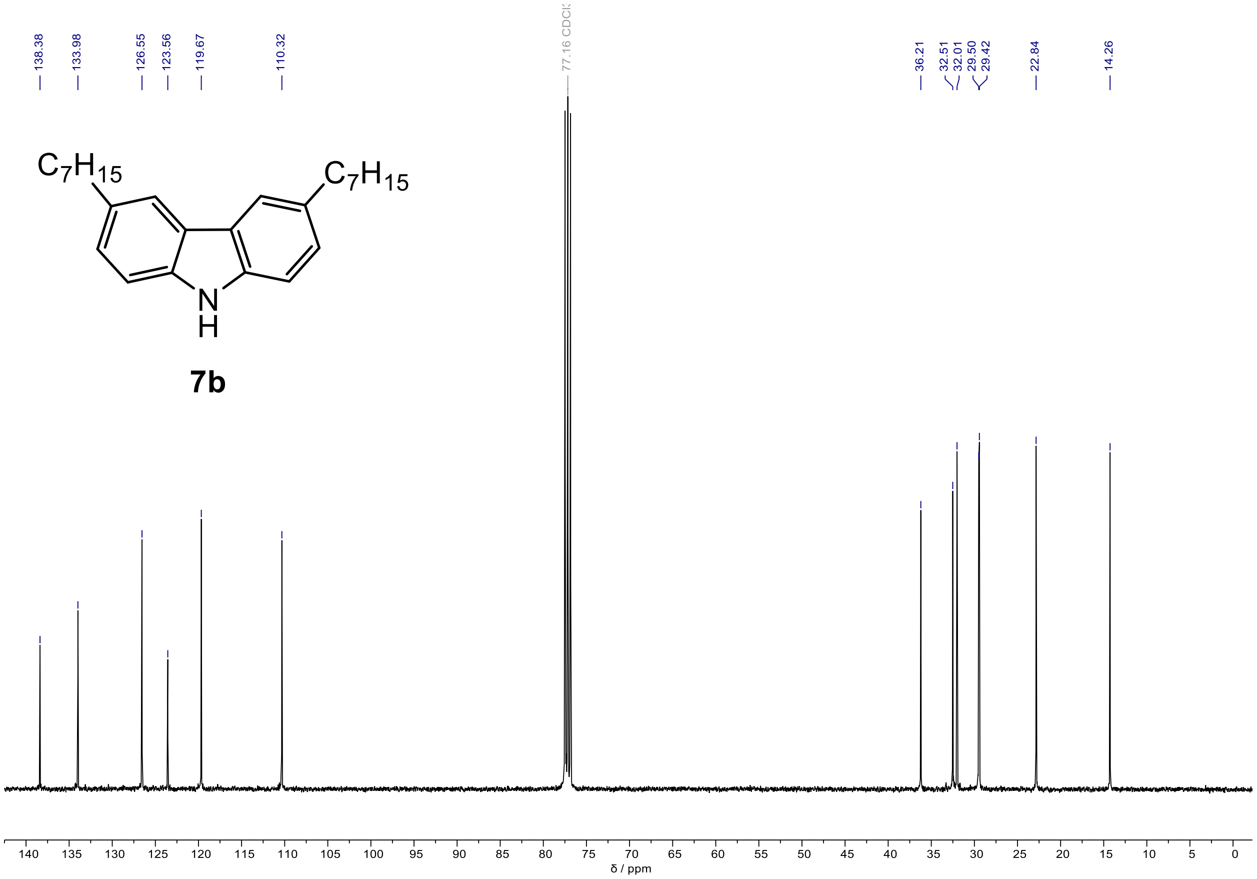

Supplementary Figure 12. ^{13}C NMR of **7b** (101 MHz, 298 K, $CDCl_3$).

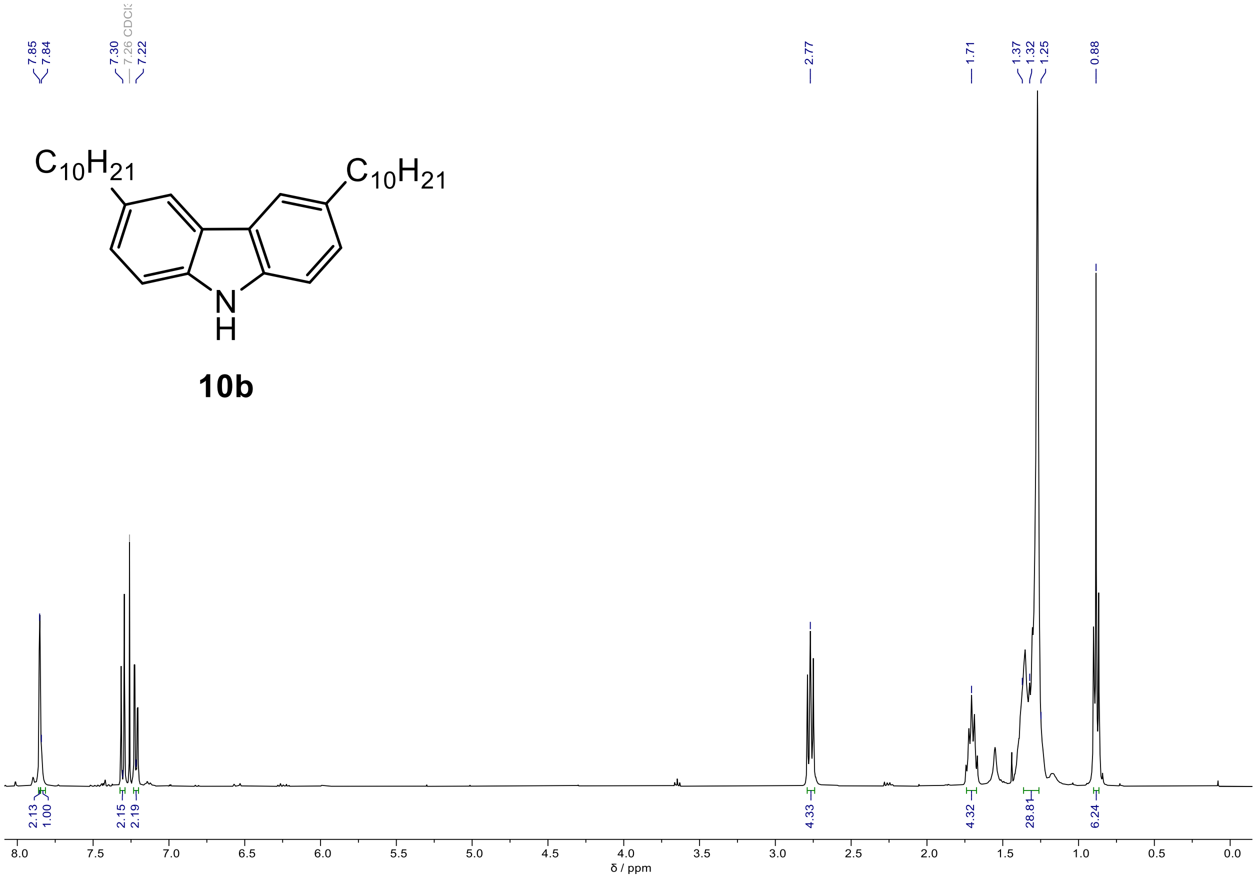

Supplementary Figure 13. ^{1}H NMR of **10b** (400 MHz, 298 K, $CDCl_3$).

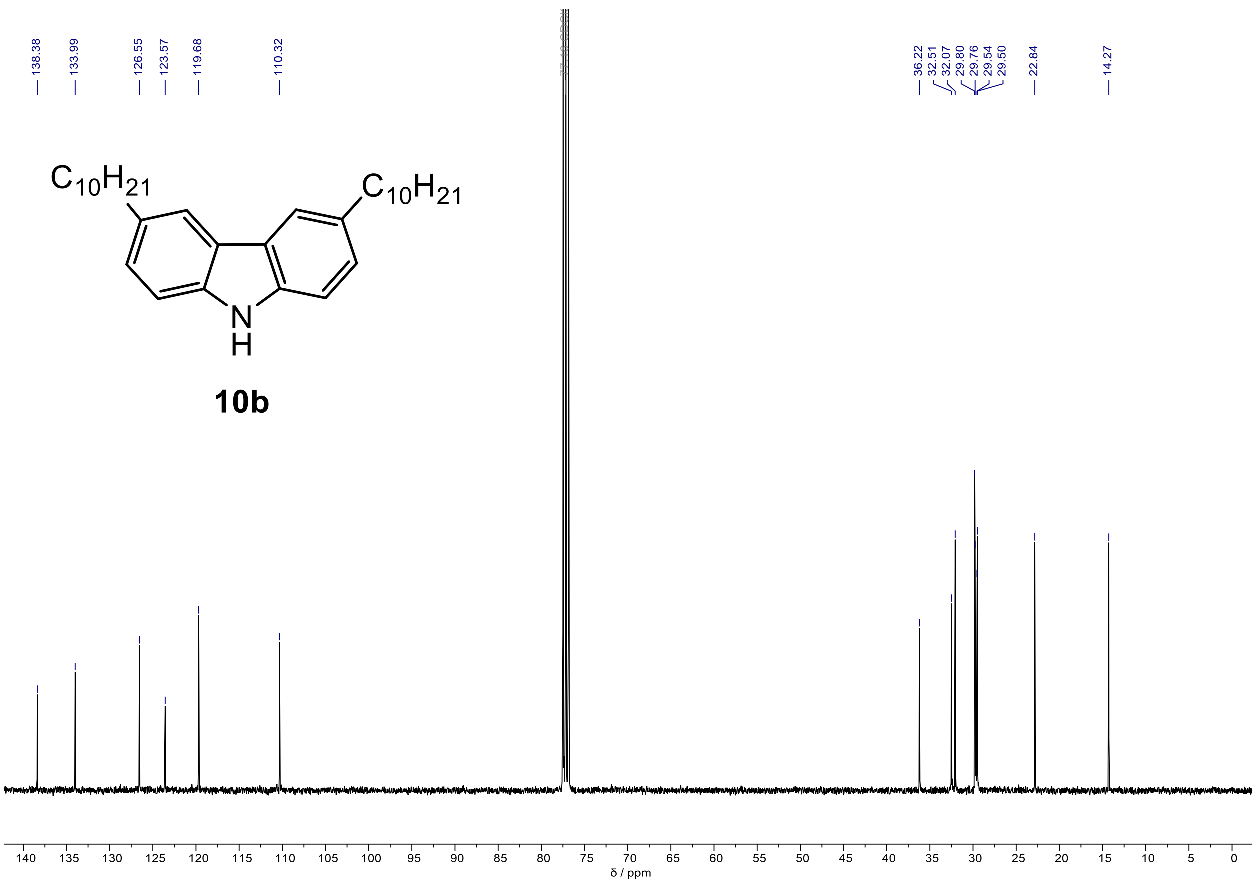

Supplementary Figure 14. ^{13}C NMR of **10b** (101 MHz, 298 K, $CDCl_3$).

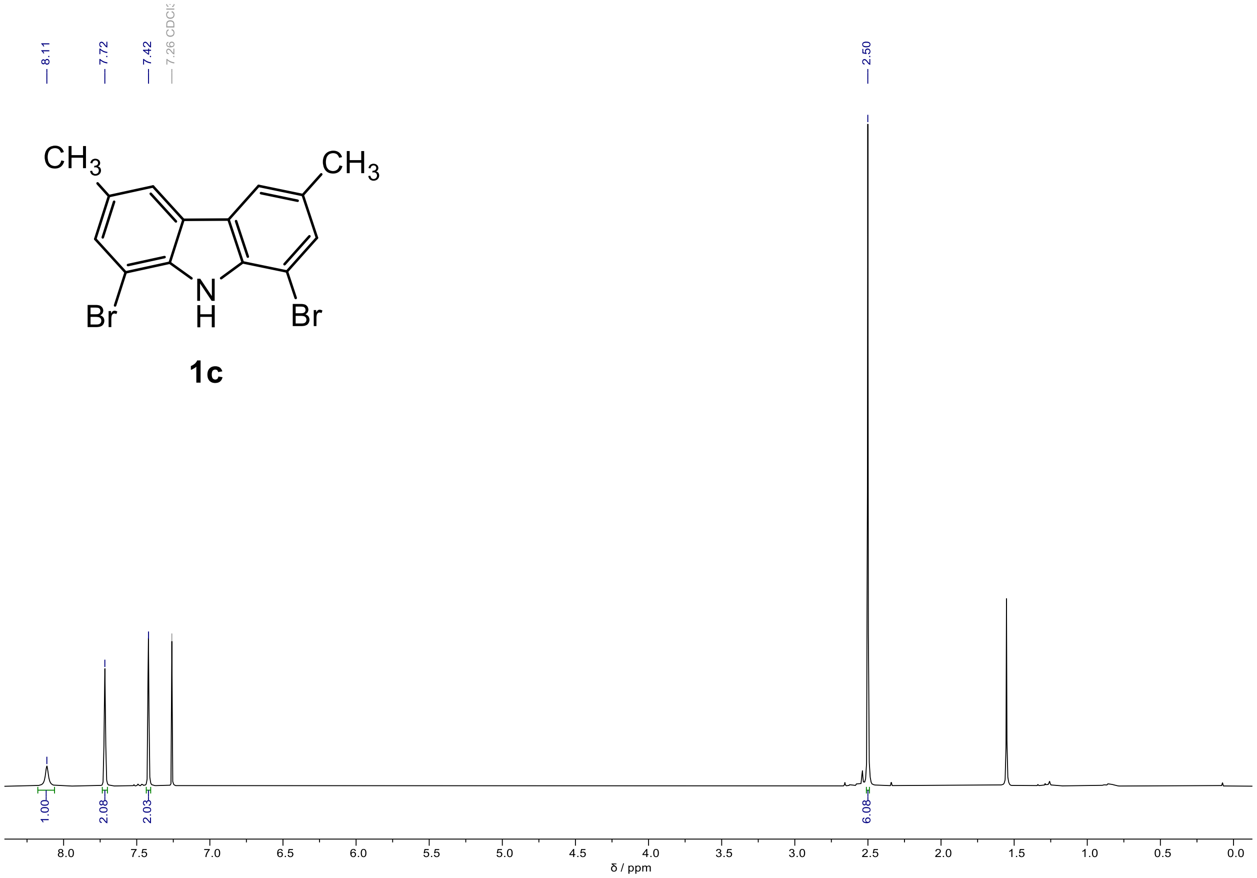

Supplementary Figure 15. ^{1}H NMR of **1c** (400 MHz, 298 K, $CDCl_3$).

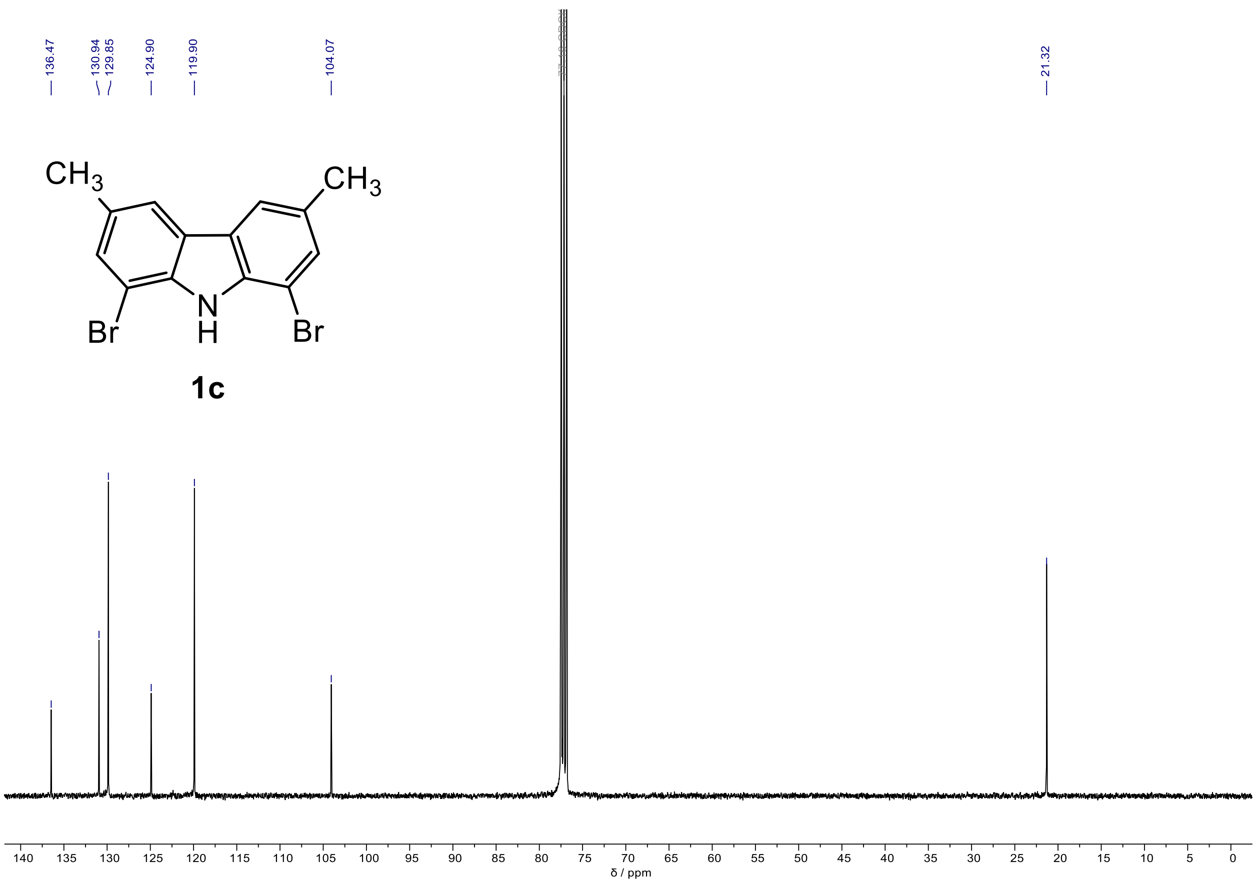

Supplementary Figure 16. ^{13}C NMR of **1c** (101 MHz, 298 K, $CDCl_3$).

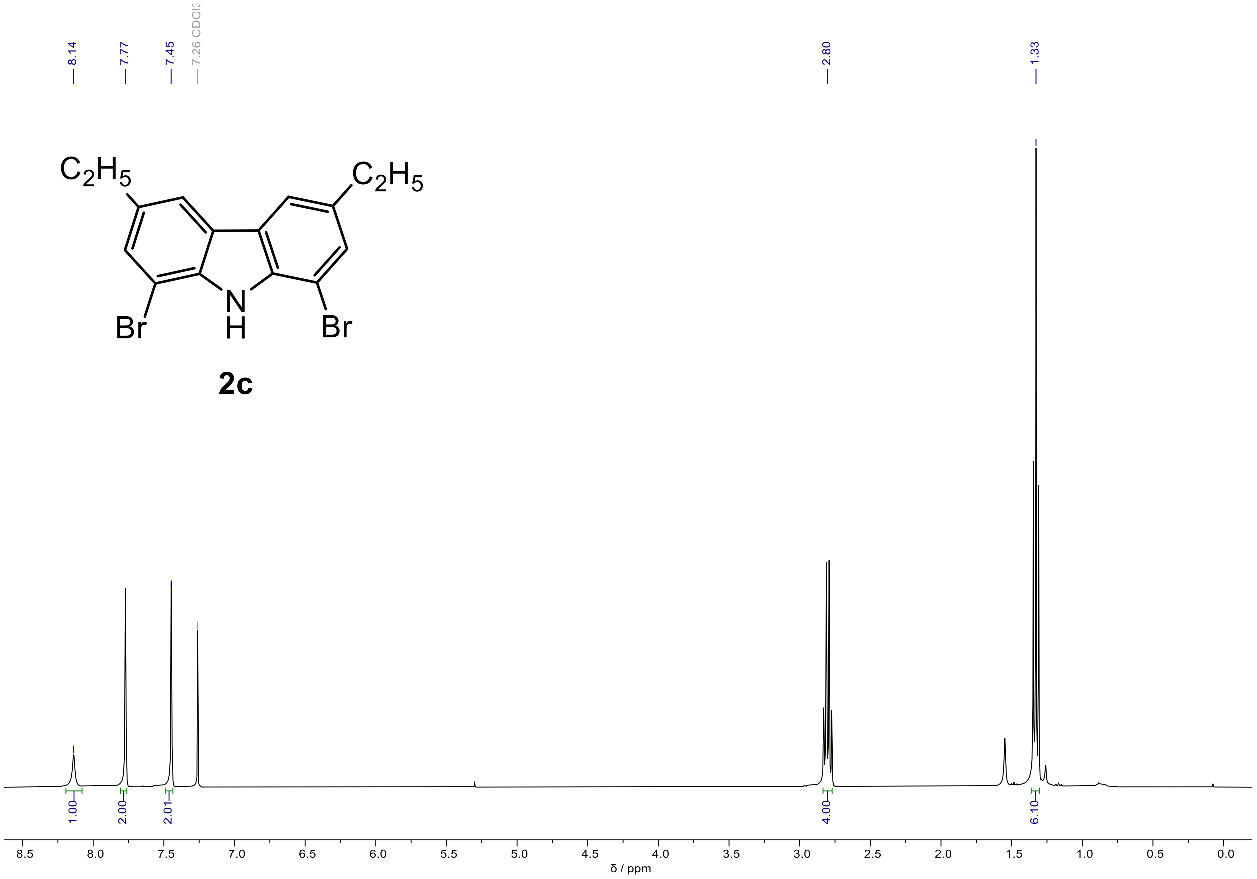

Supplementary Figure 17. ^{1}H NMR of **2c** (400 MHz, 298 K, $CDCl_3$).

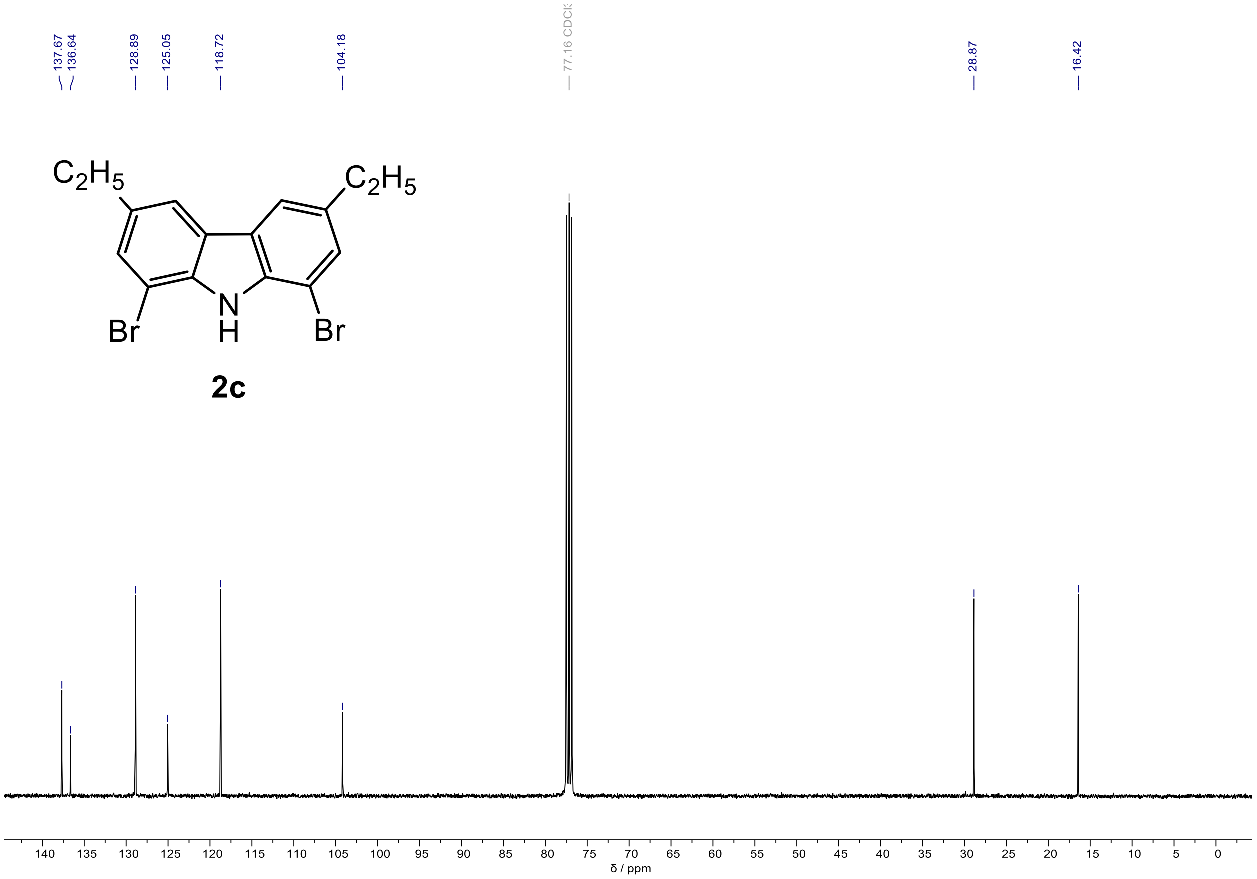

Supplementary Figure 18. ^{13}C NMR of **2c** (101 MHz, 298 K, $CDCl_3$).

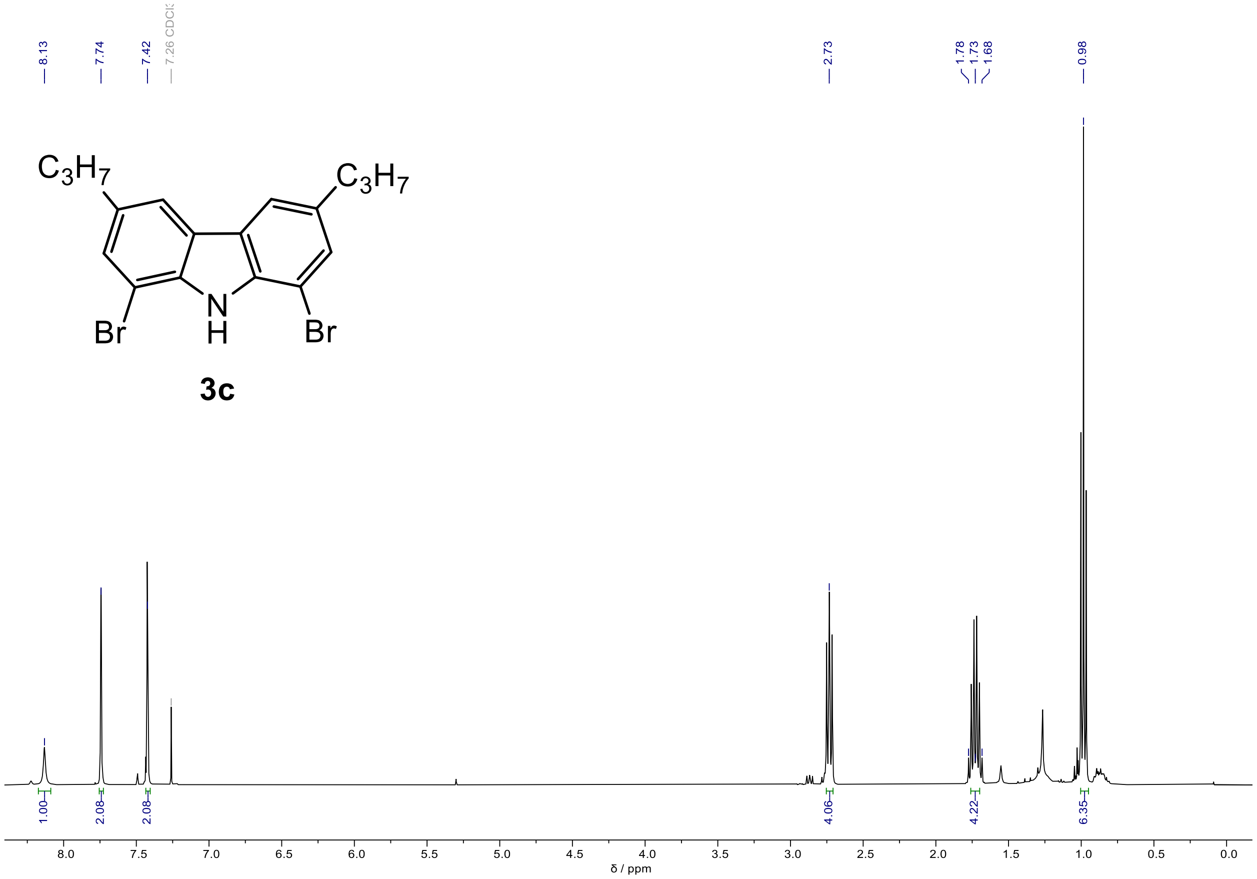

Supplementary Figure 19. ^{1}H NMR of **3c** (400 MHz, 298 K, $CDCl_3$).

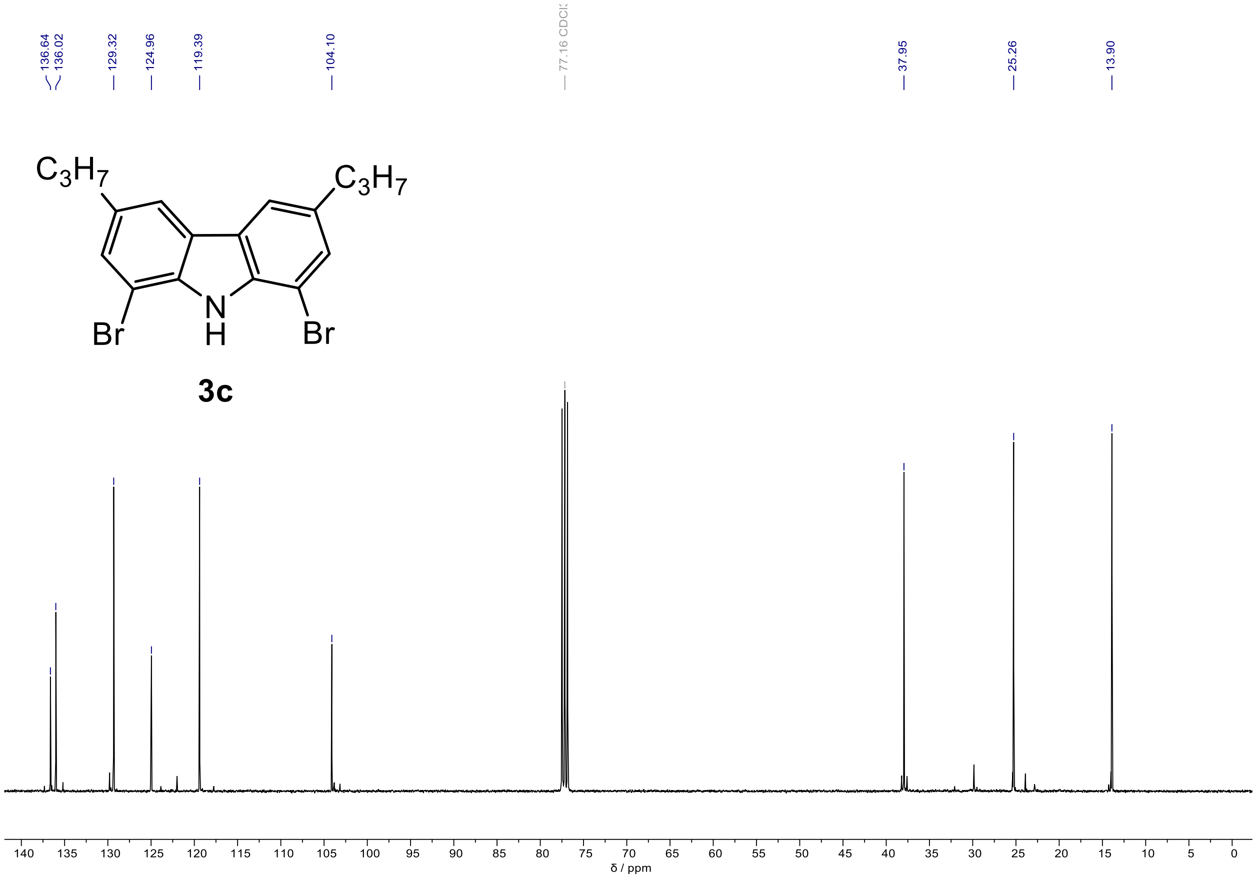

Supplementary Figure 20. ^{13}C NMR of **3c** (101 MHz, 298 K, $CDCl_3$).

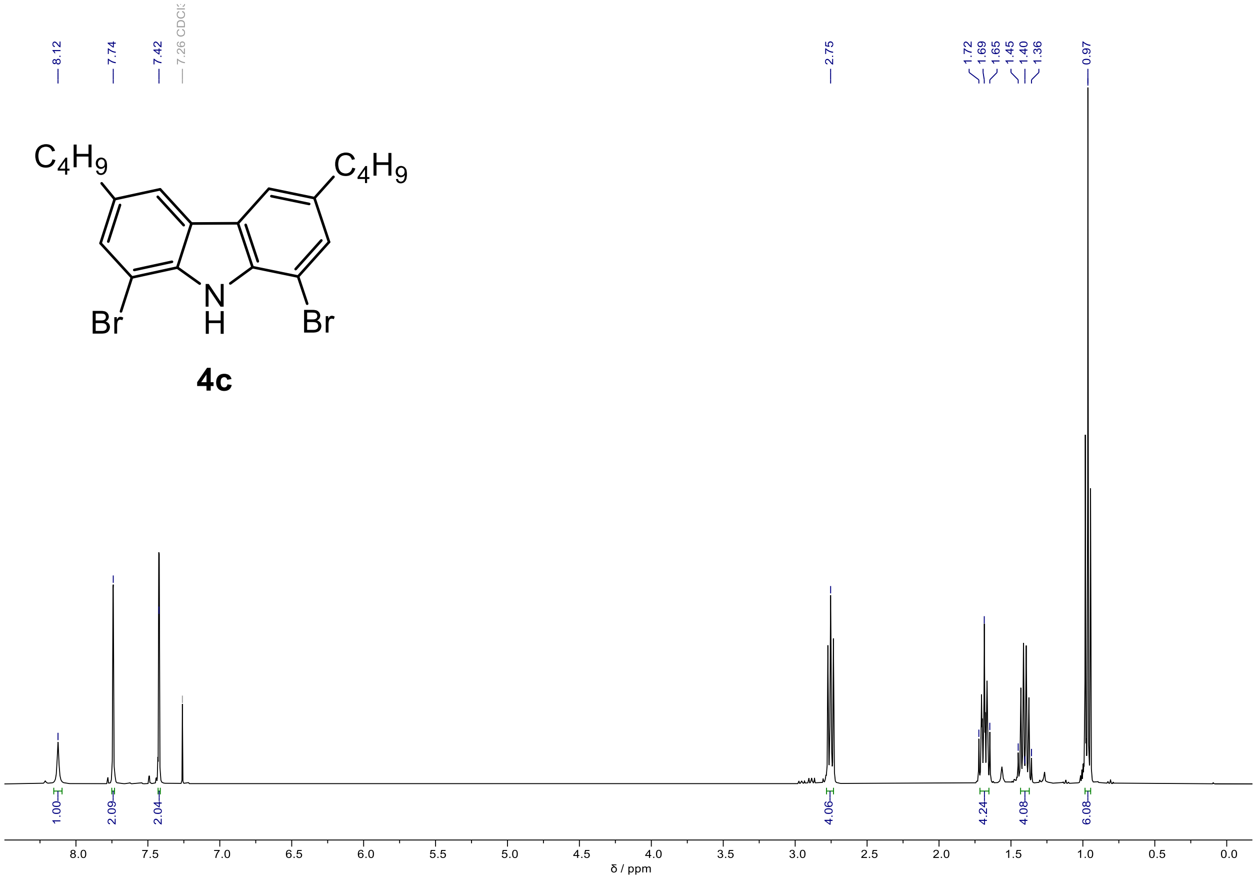

Supplementary Figure 21. ^{1}H NMR of **4c** (400 MHz, 298 K, $CDCl_3$).

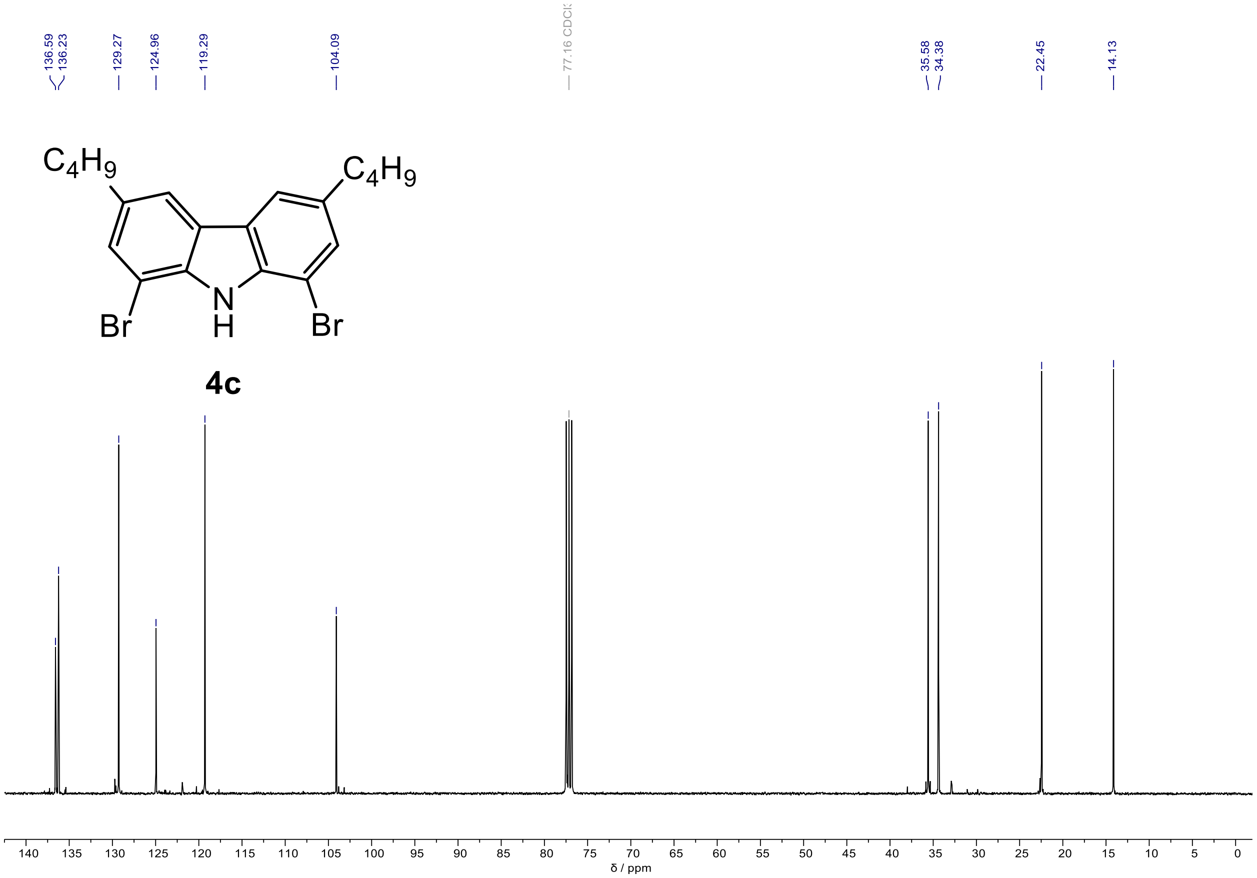

Supplementary Figure 22. ^{13}C NMR of **4c** (101 MHz, 298 K, $CDCl_3$).

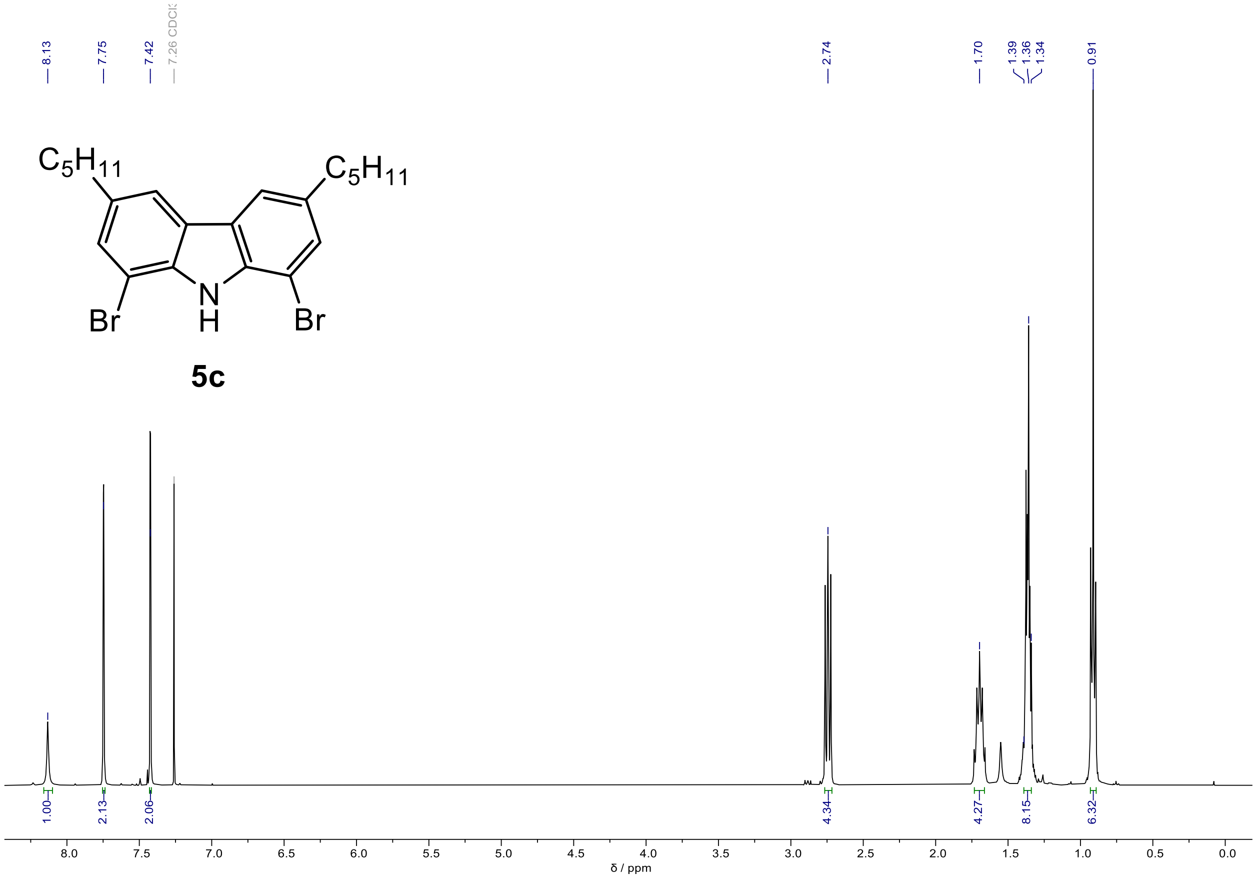

Supplementary Figure 23. ^{1}H NMR of **5c** (400 MHz, 298 K, $CDCl_3$).

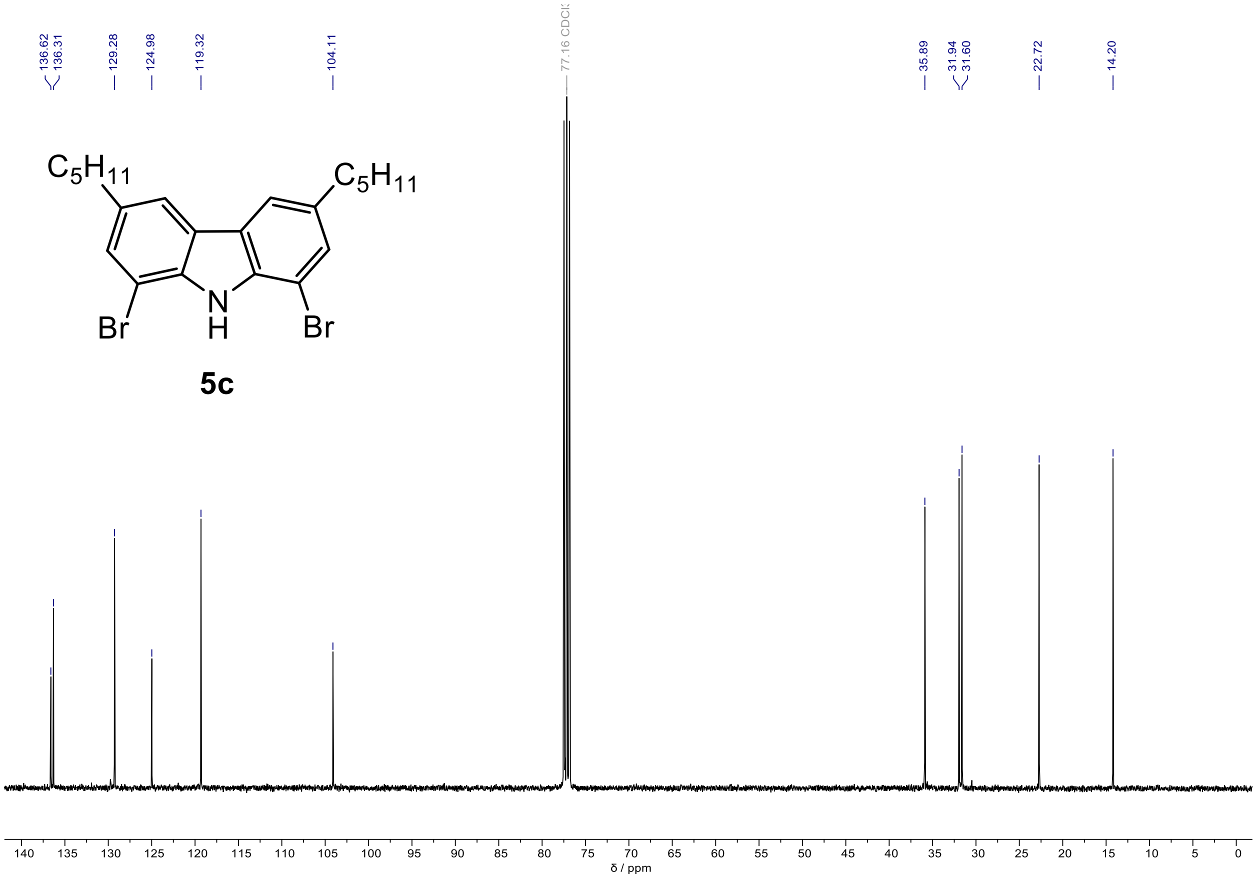

Supplementary Figure 24. ^{13}C NMR of **5c** (101 MHz, 298 K, $CDCl_3$).

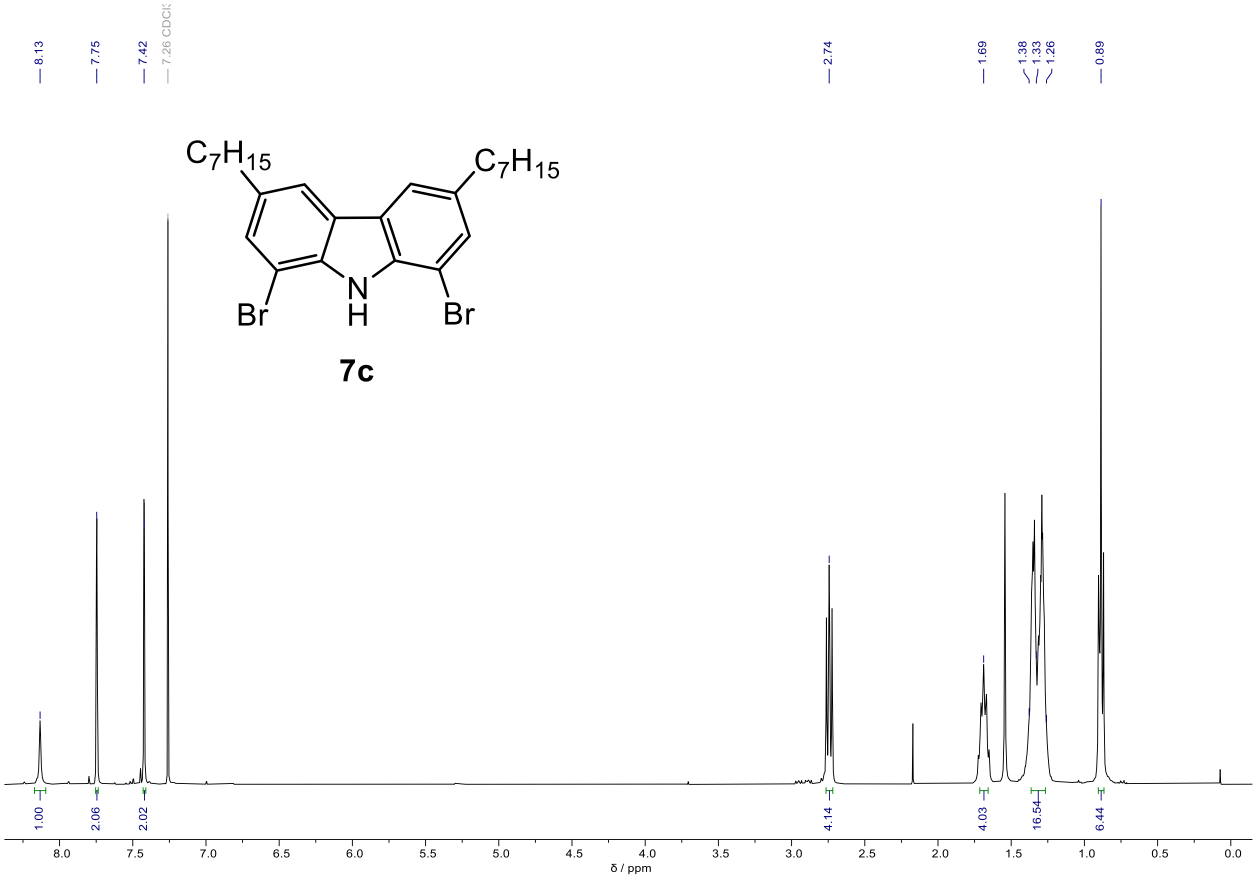

Supplementary Figure 25. ^{1}H NMR of **7c** (400 MHz, 298 K, $CDCl_3$).

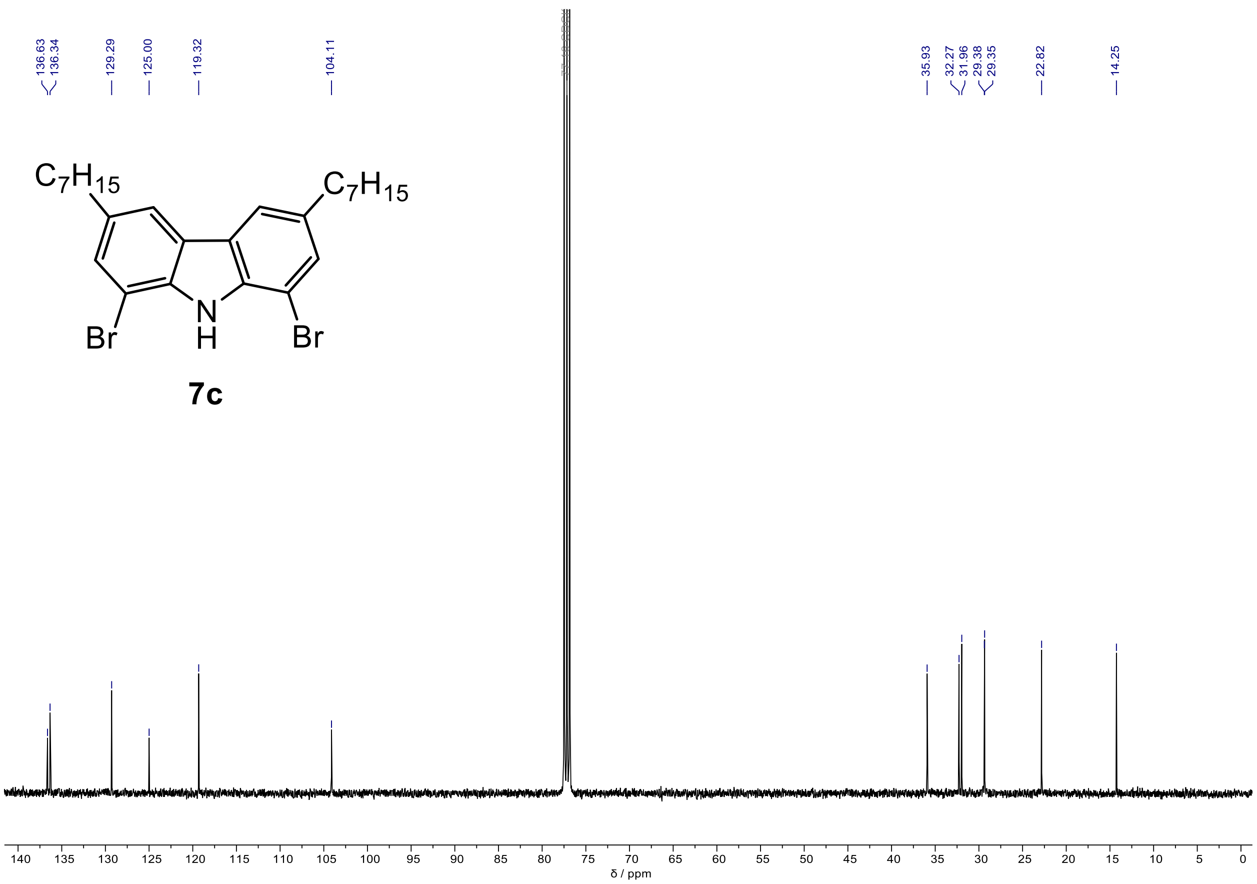

Supplementary Figure 26. ^{13}C NMR of **7c** (101 MHz, 298 K, $CDCl_3$).

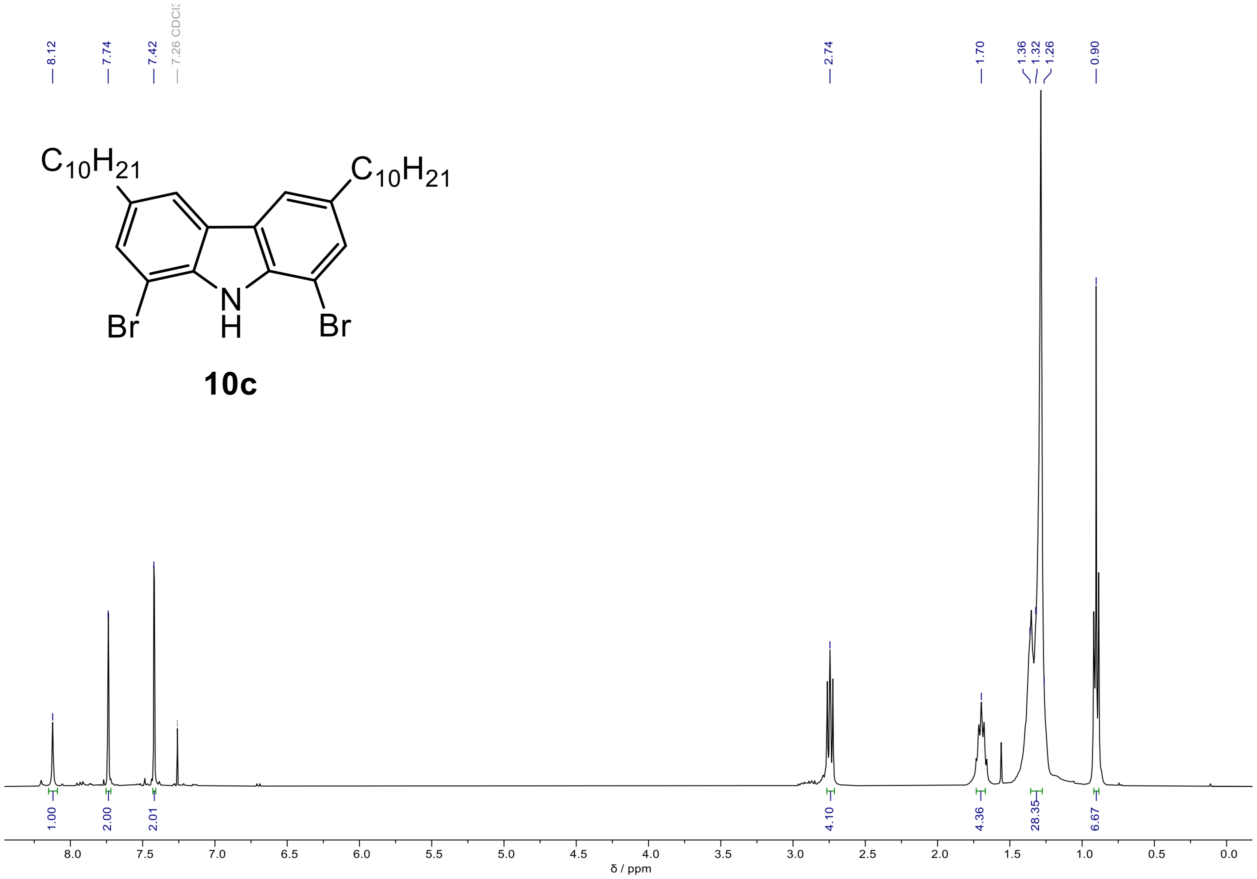

Supplementary Figure 27. ^{1}H NMR of **10c** (400 MHz, 298 K, $CDCl_3$).

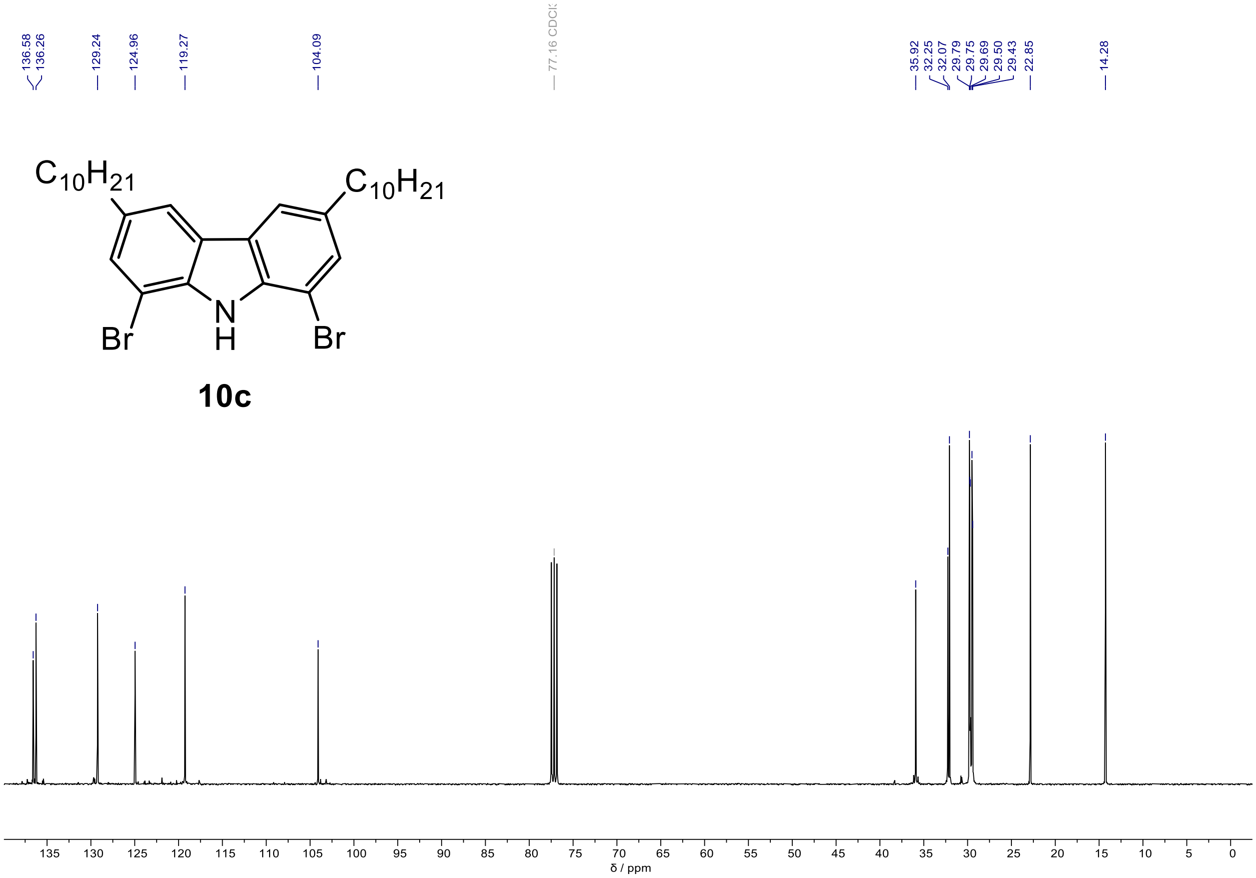

Supplementary Figure 28. ^{13}C NMR of **10c** (101 MHz, 298 K, $CDCl_3$).

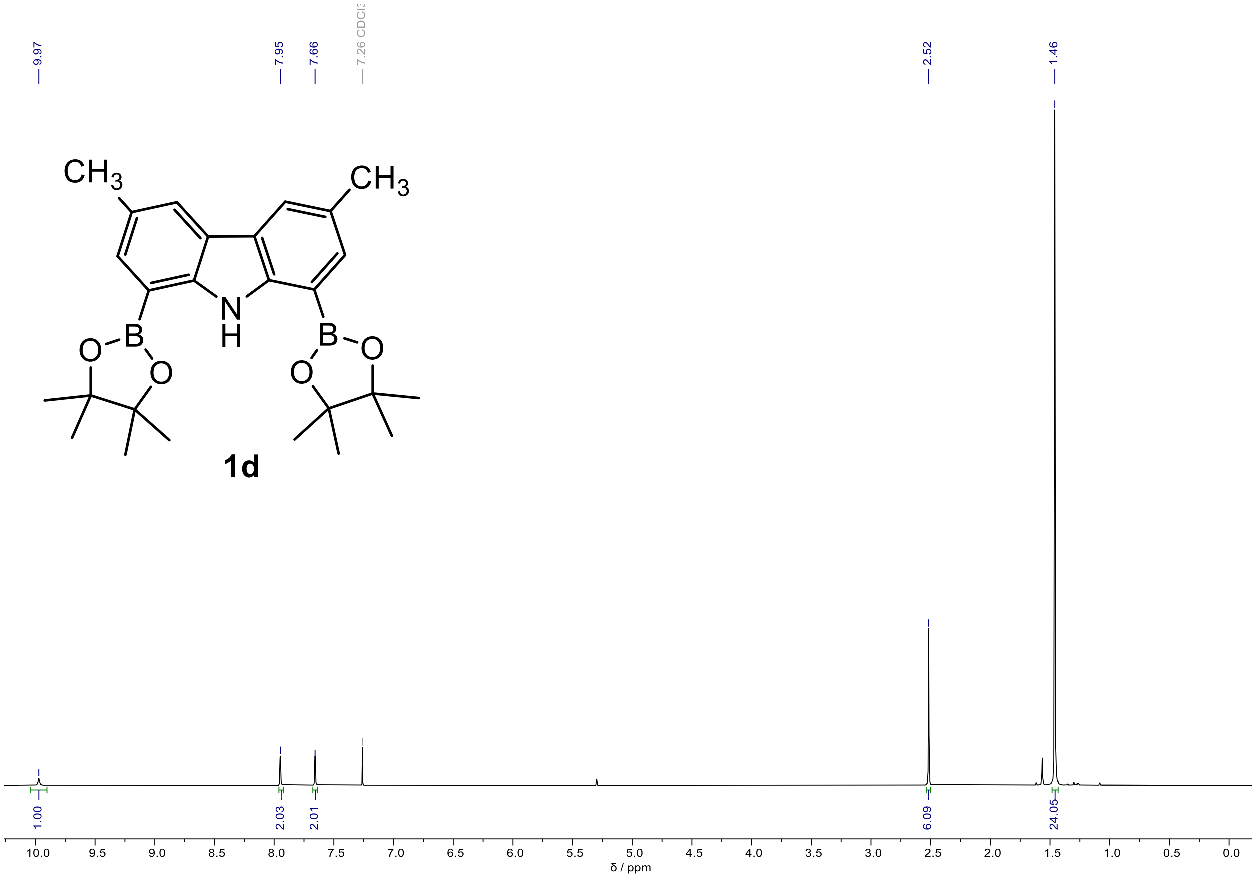

Supplementary Figure 29. ^{1}H NMR of **1d** (400 MHz, 298 K, $CDCl_3$).

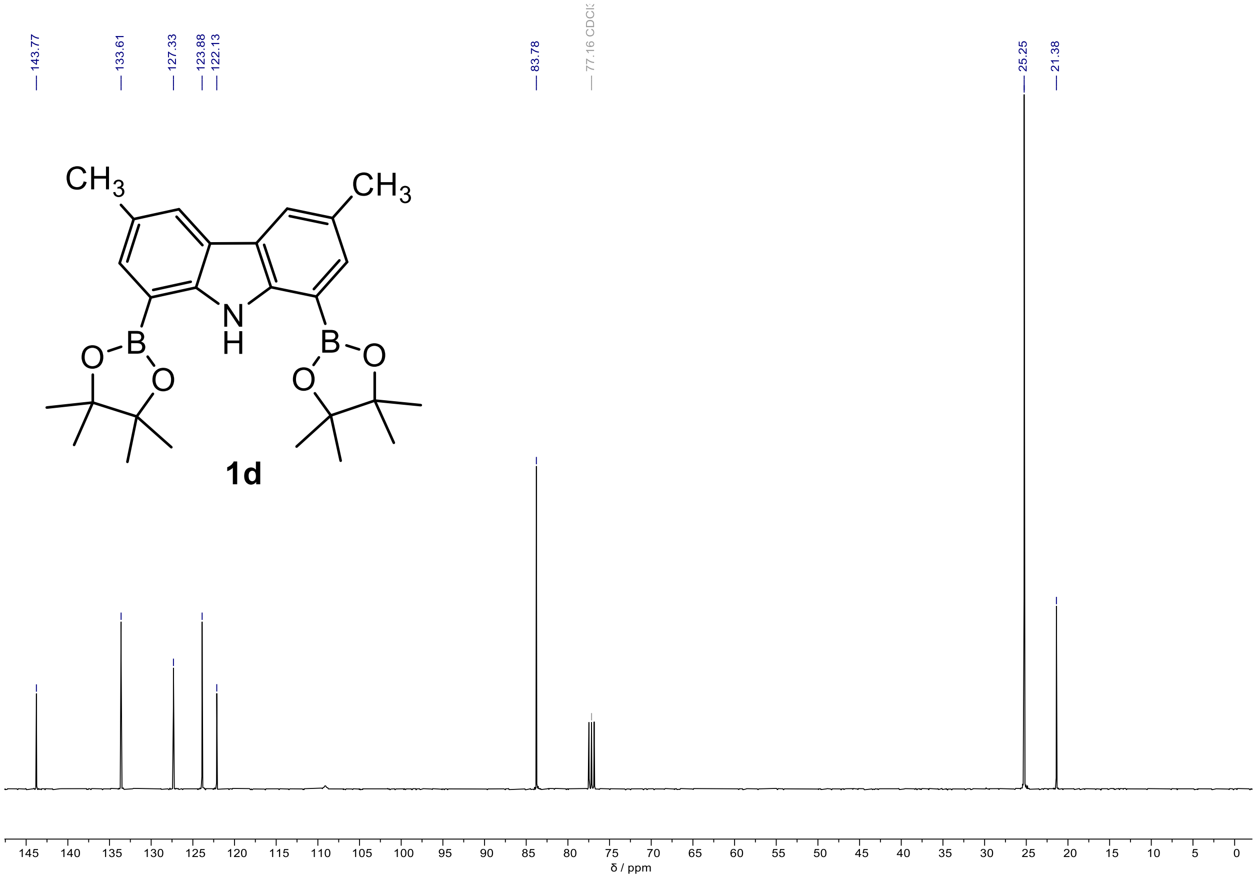

Supplementary Figure 30. ^{13}C NMR of **1d** (101 MHz, 298 K, $CDCl_3$).

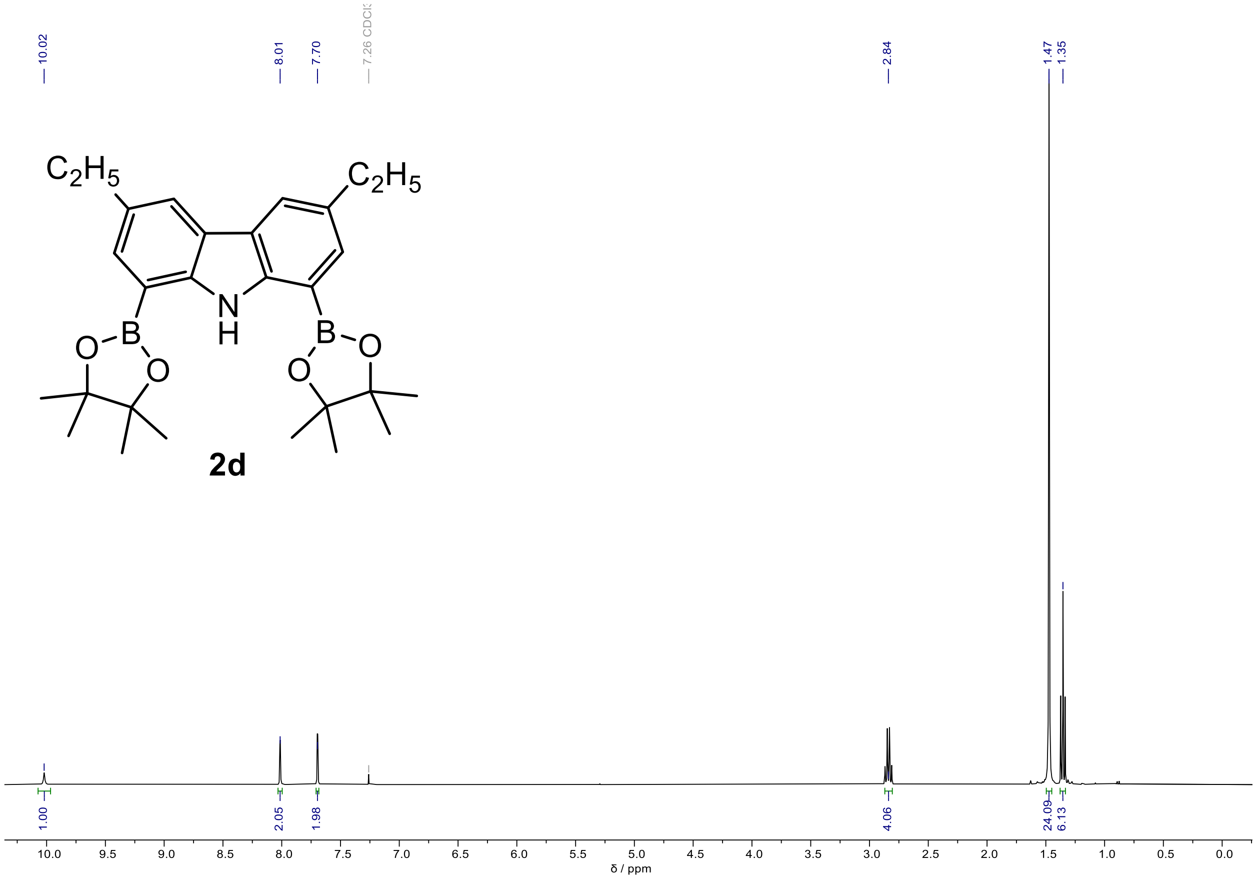

Supplementary Figure 31. ^{1}H NMR of **2d** (400 MHz, 298 K, $CDCl_3$).

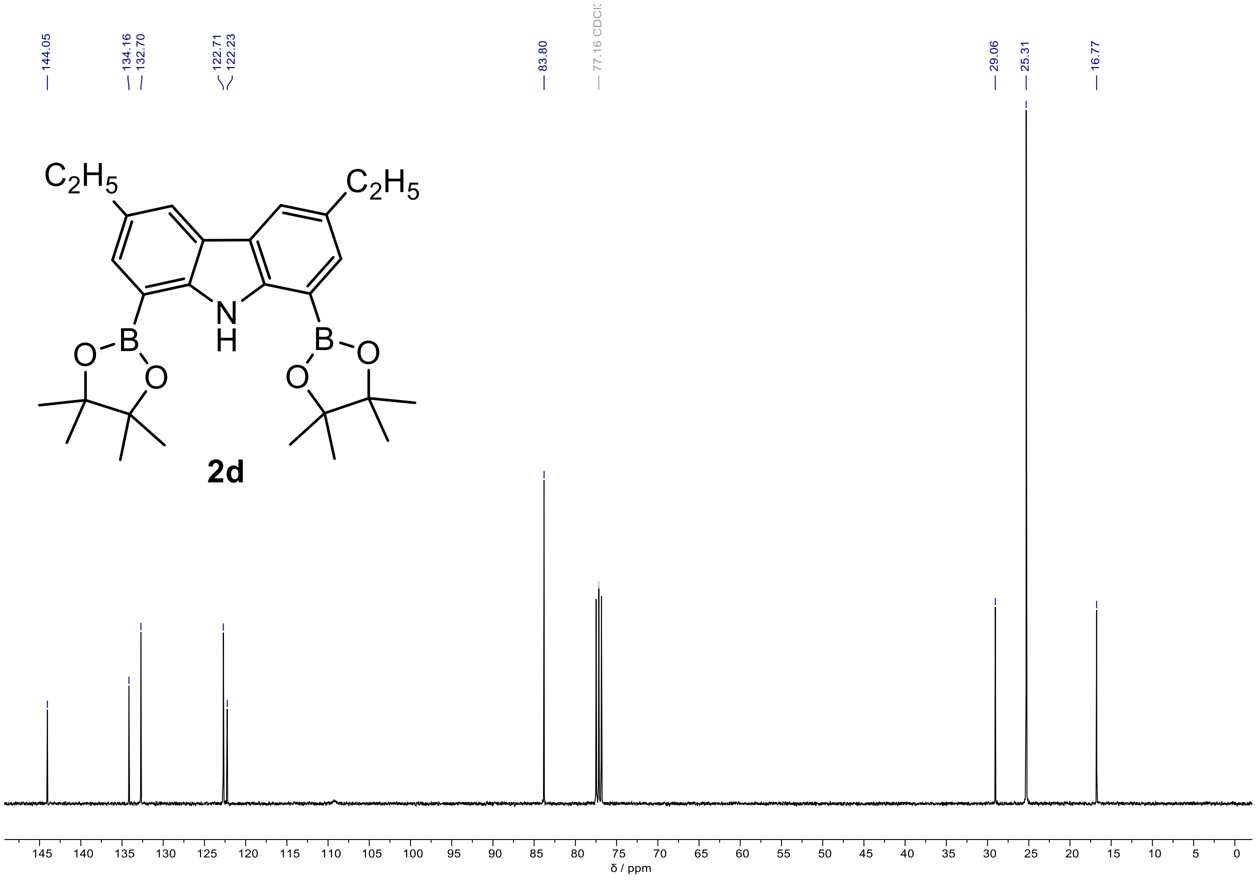

Supplementary Figure 32. ^{13}C NMR of **2d** (101 MHz, 298 K, $CDCl_3$).

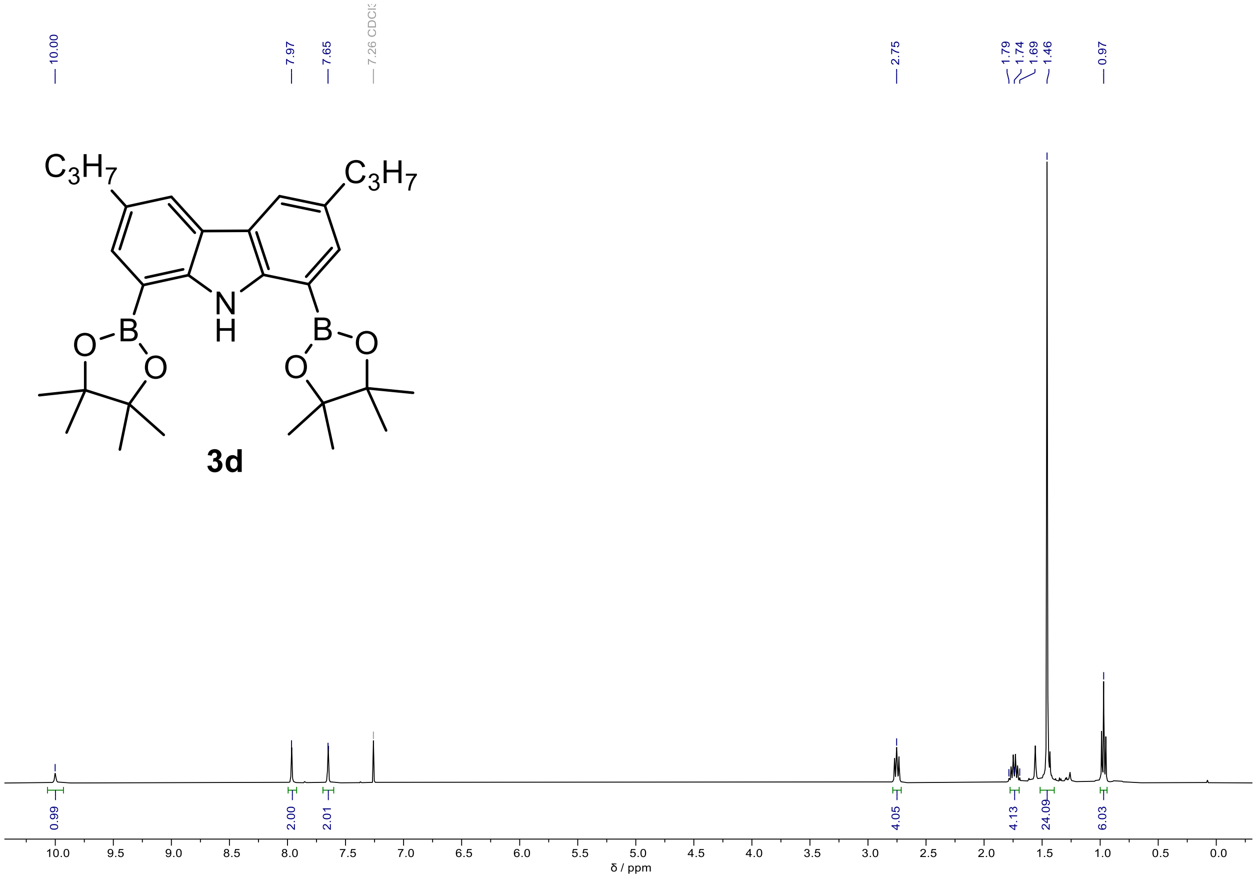

Supplementary Figure 33. ^{1}H NMR of **3d** (400 MHz, 298 K, $CDCl_3$).

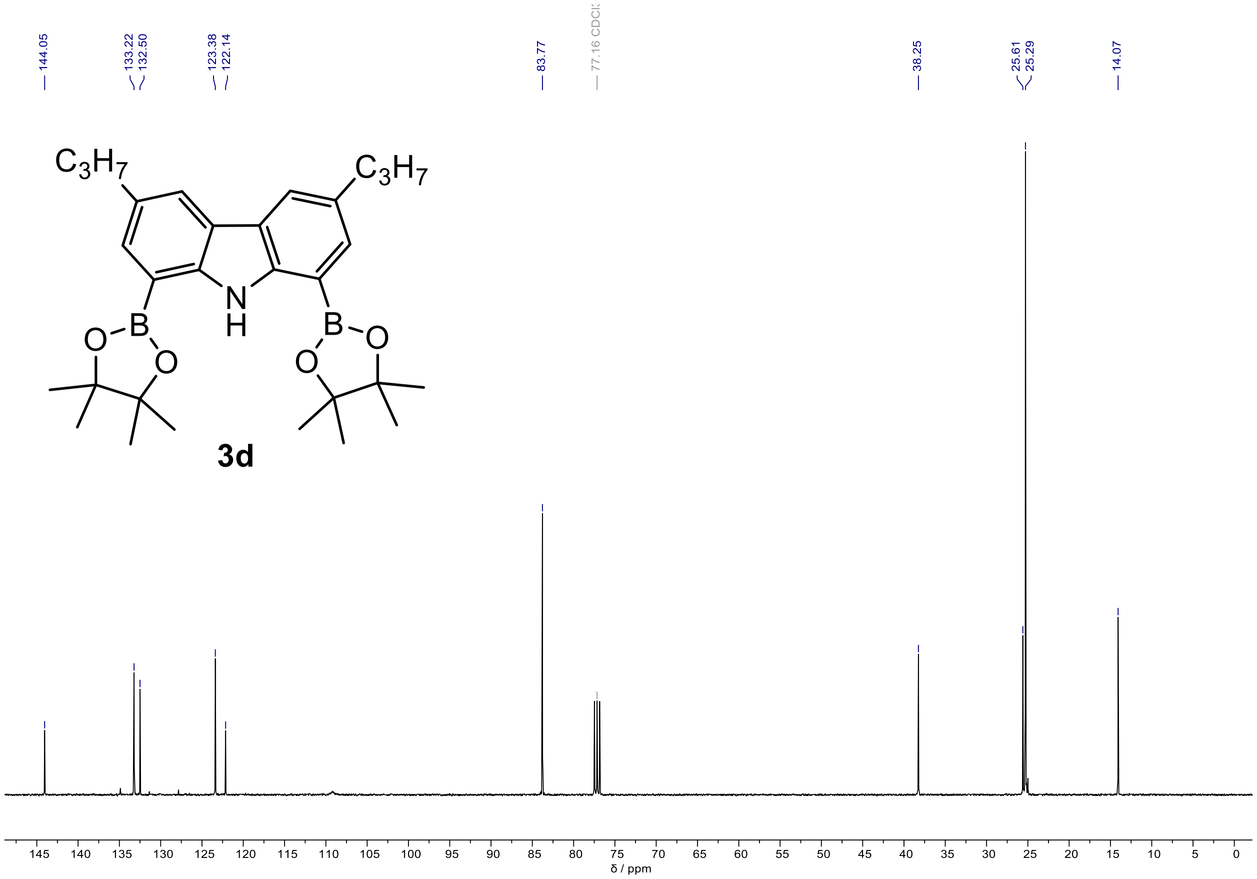

Supplementary Figure 34. ^{13}C NMR of **3d** (101 MHz, 298 K, $CDCl_3$).

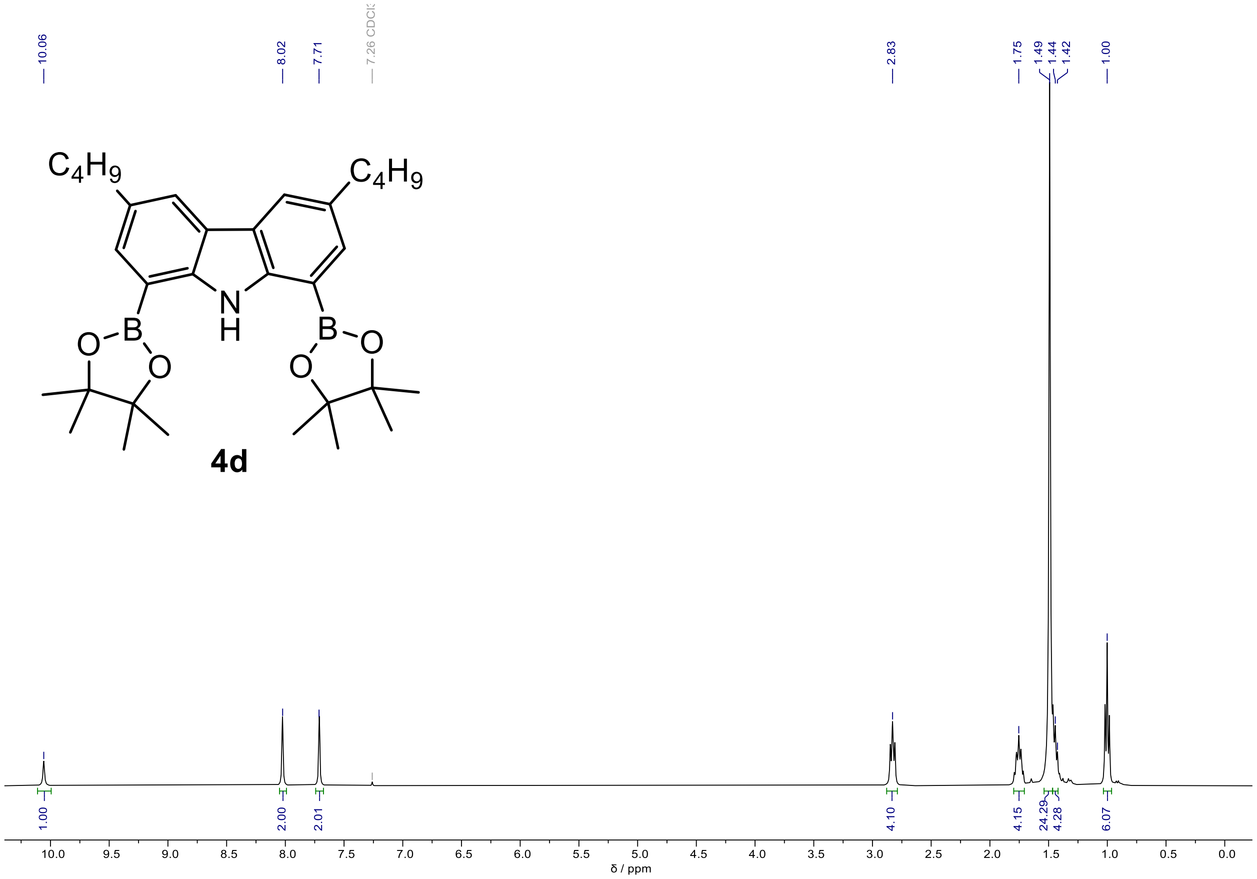

Supplementary Figure 35. ^{1}H NMR of **4d** (400 MHz, 298 K, $CDCl_3$).

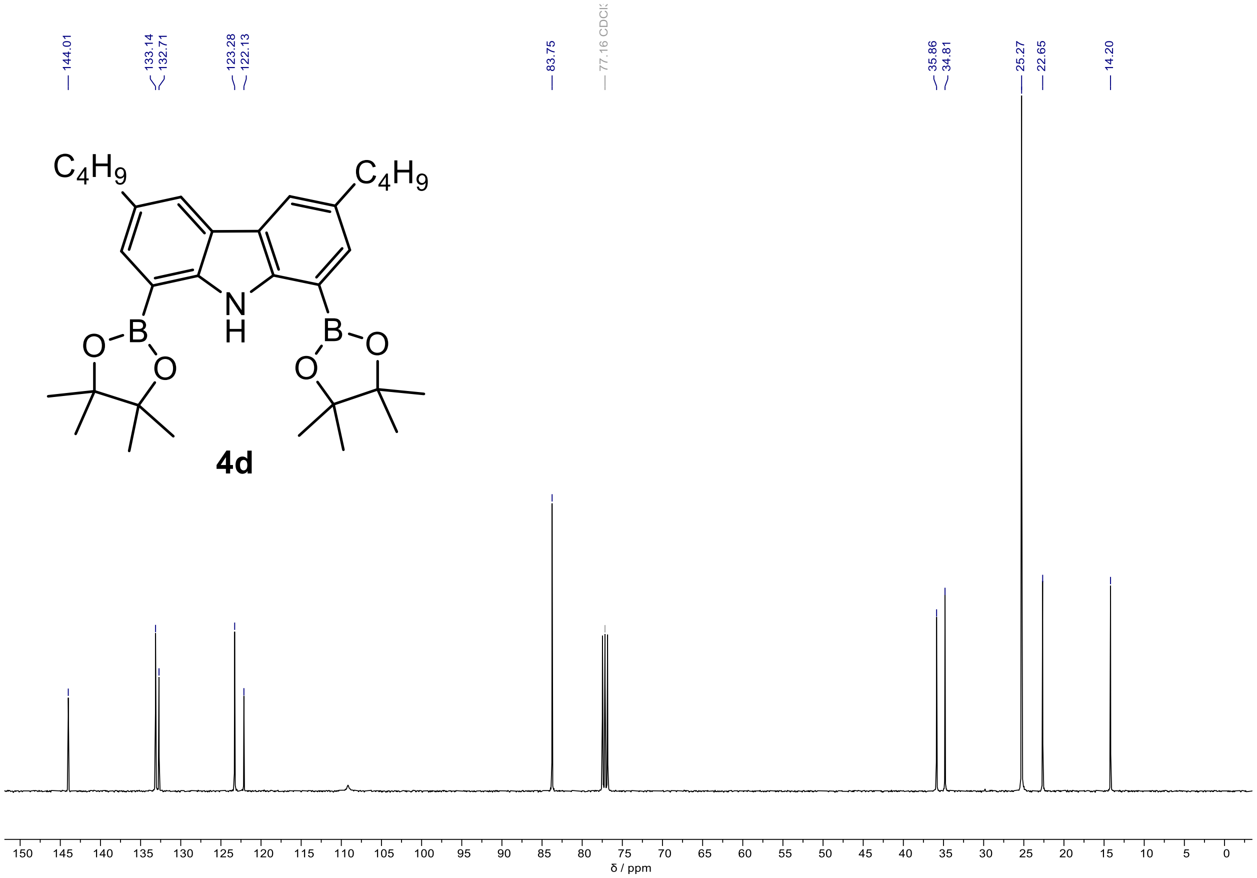

Supplementary Figure 36. ^{13}C NMR of **4d** (101 MHz, 298 K, $CDCl_3$).

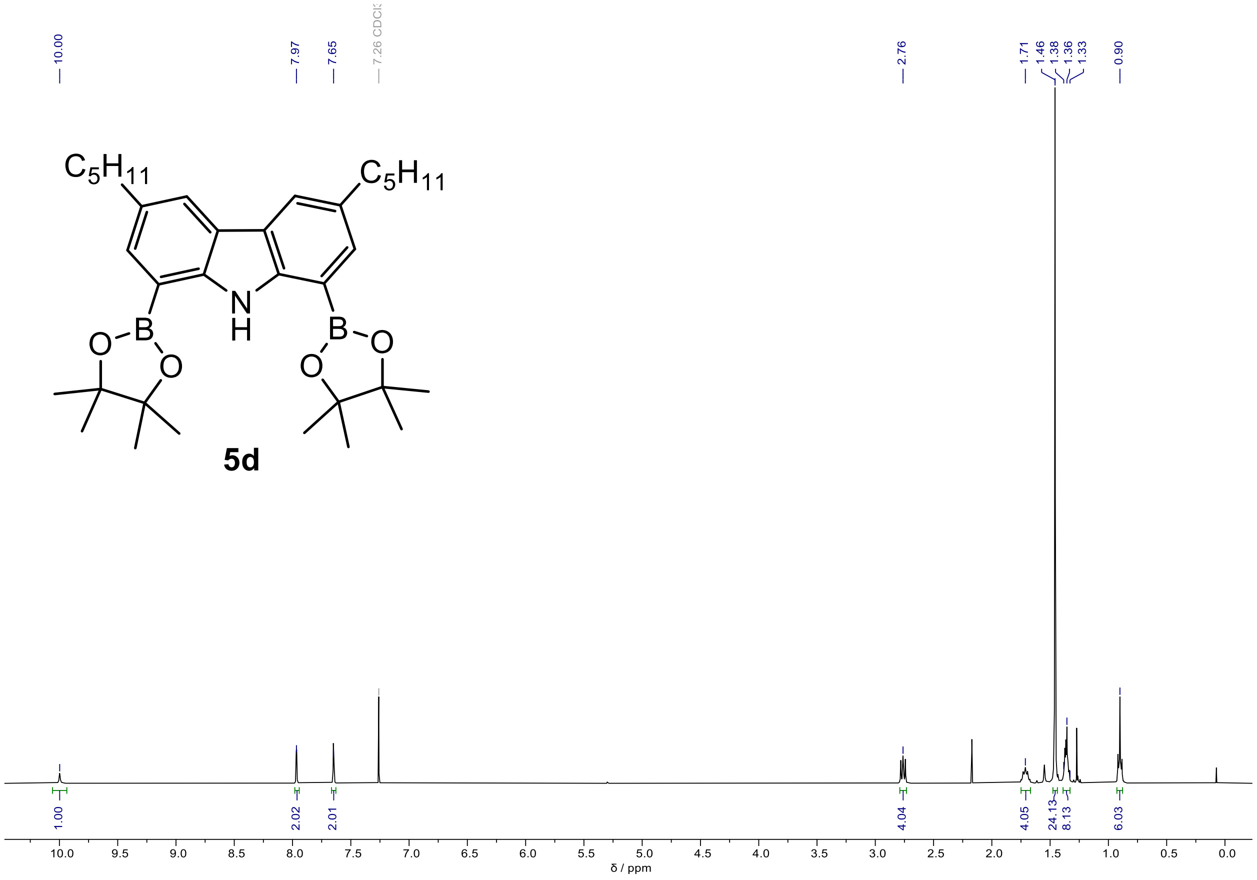

Supplementary Figure 37. ^{1}H NMR of **5d** (400 MHz, 298 K, $CDCl_3$).

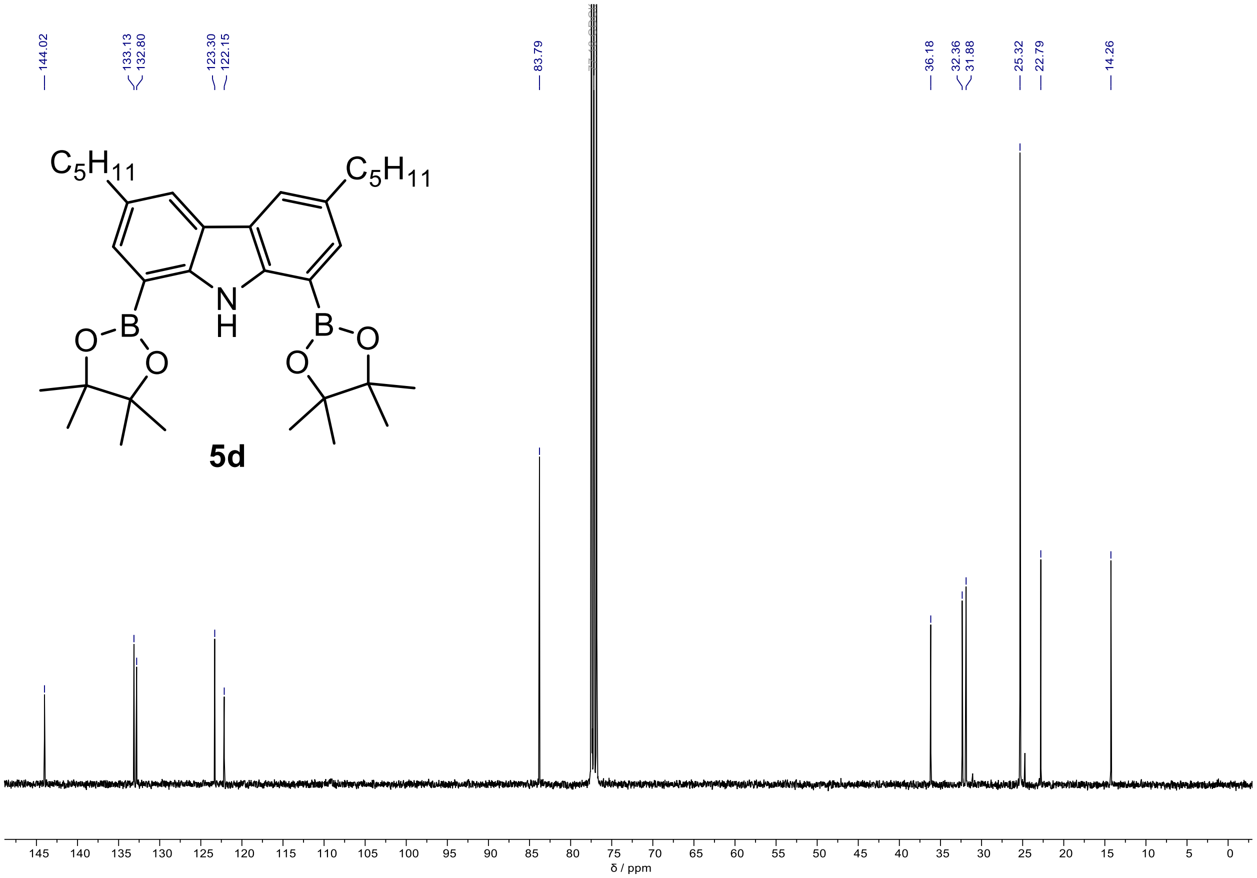

Supplementary Figure 38. ^{13}C NMR of **5d** (101 MHz, 298 K, $CDCl_3$).

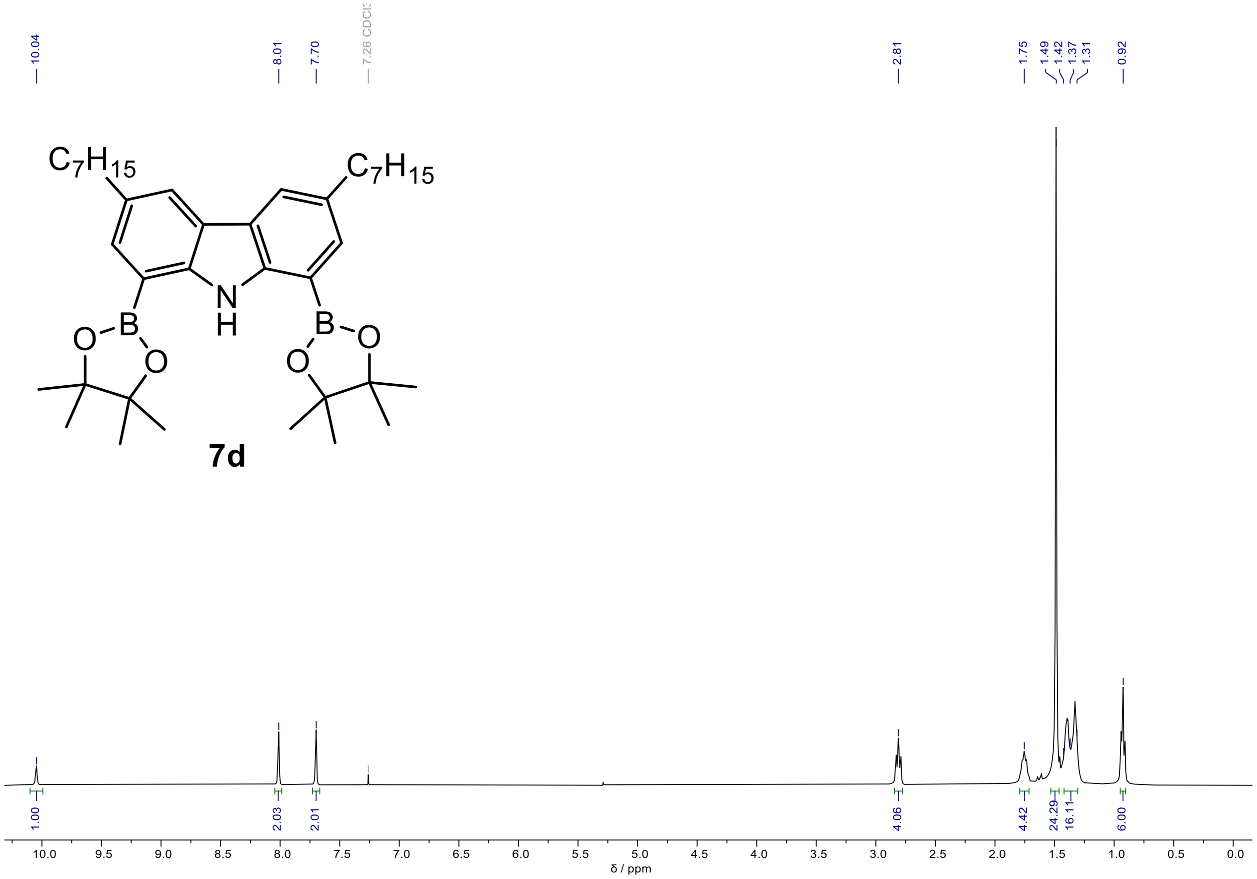

Supplementary Figure 39. ^{1}H NMR of **7d** (400 MHz, 298 K, $CDCl_3$).

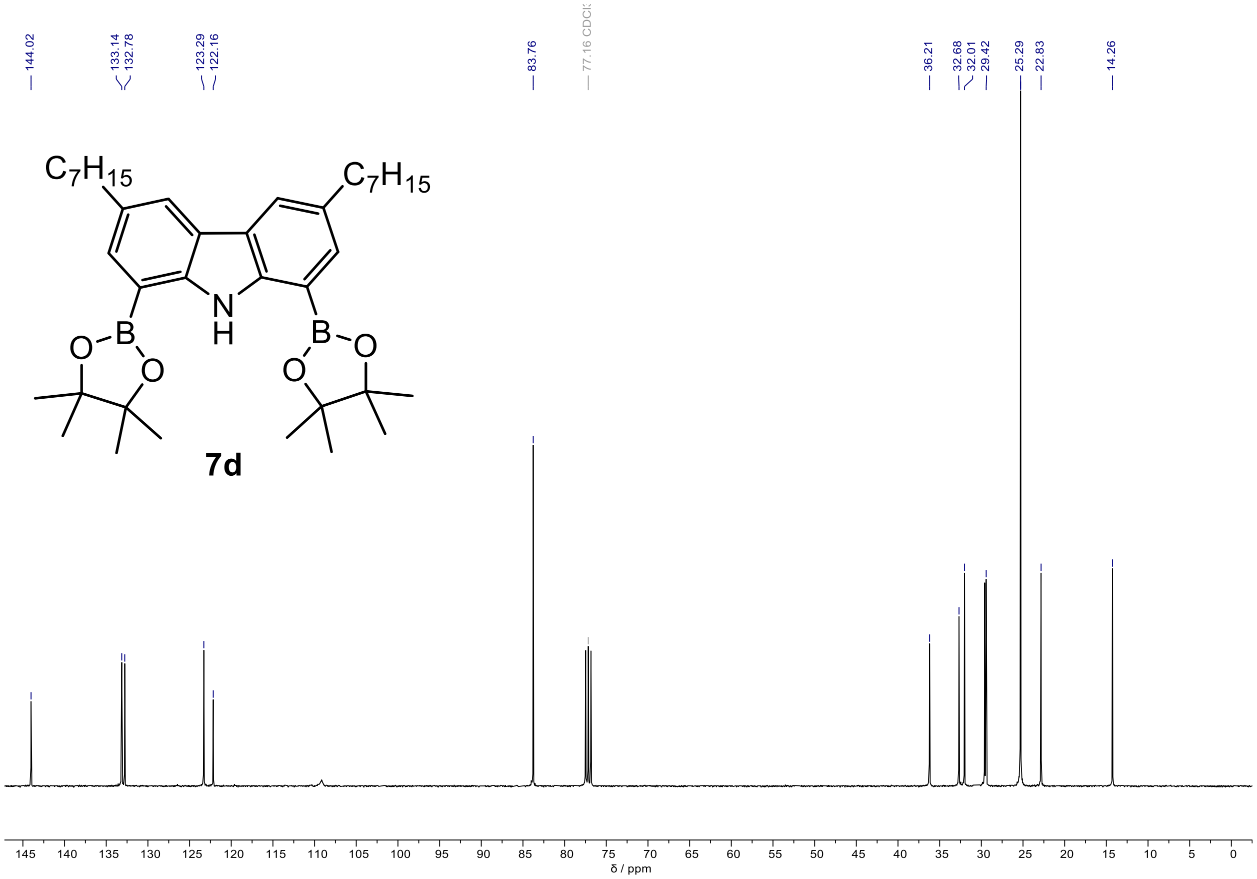

Supplementary Figure 40. ^{13}C NMR of **7d** (101 MHz, 298 K, $CDCl_3$).

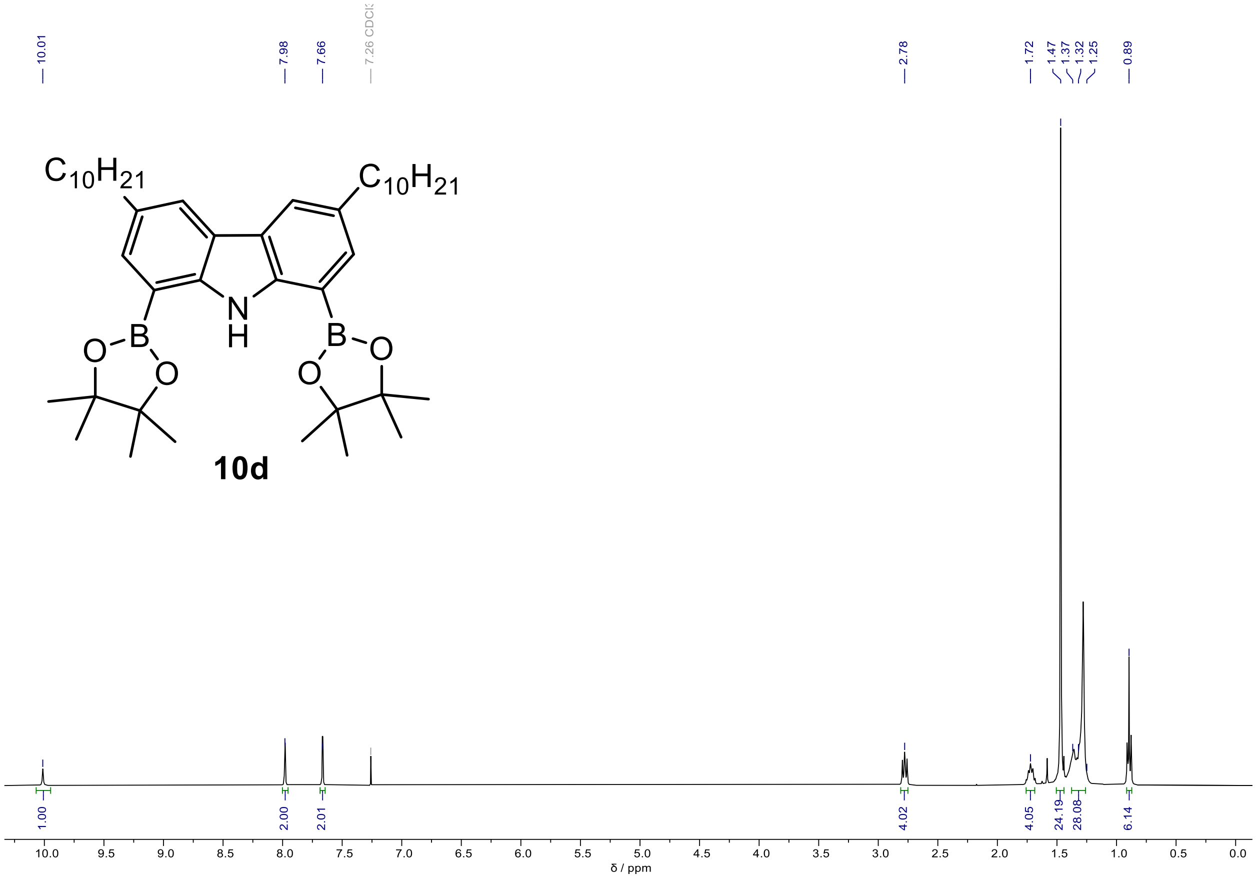

Supplementary Figure 41. ^{1}H NMR of **10d** (400 MHz, 298 K, $CDCl_3$).

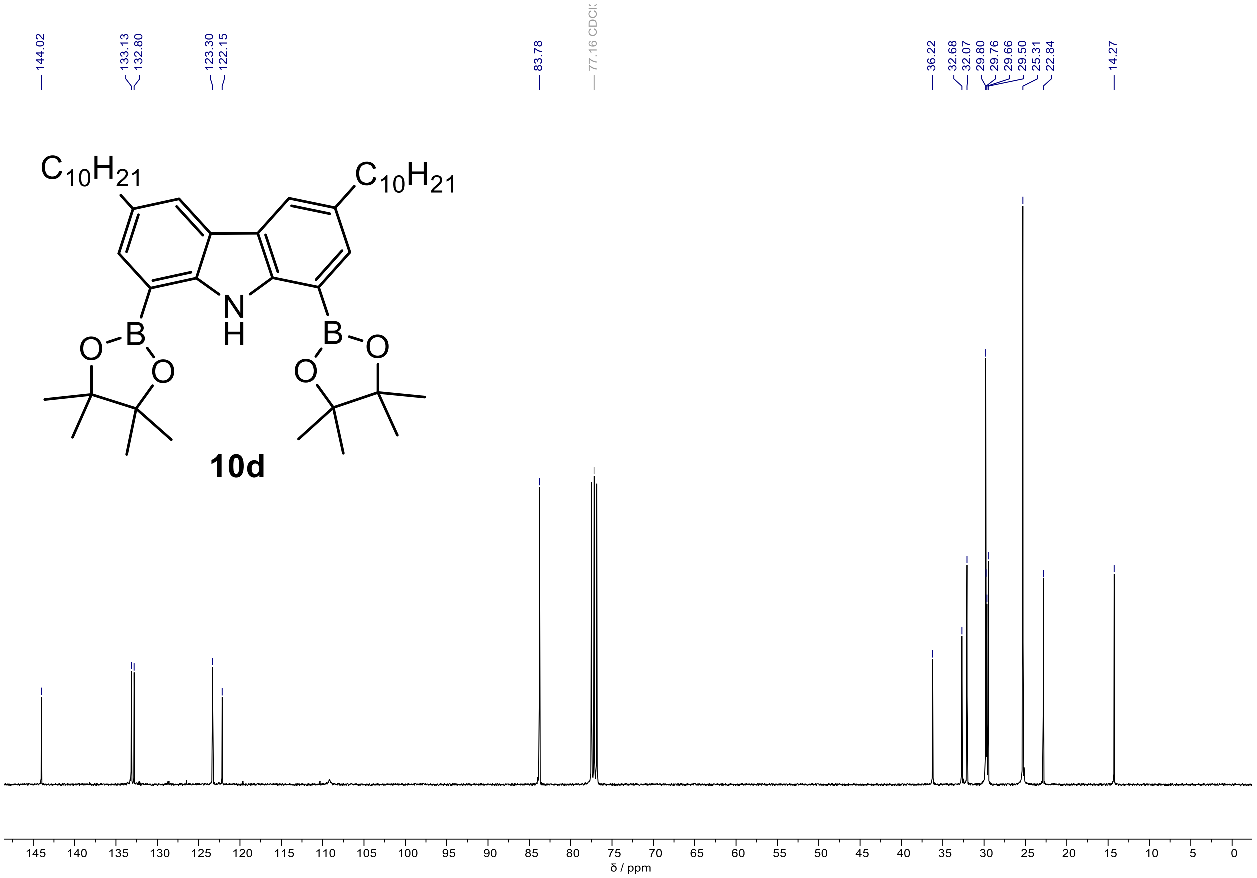

Supplementary Figure 42. ^{13}C NMR of **10d** (101 MHz, 298 K, $CDCl_3$).

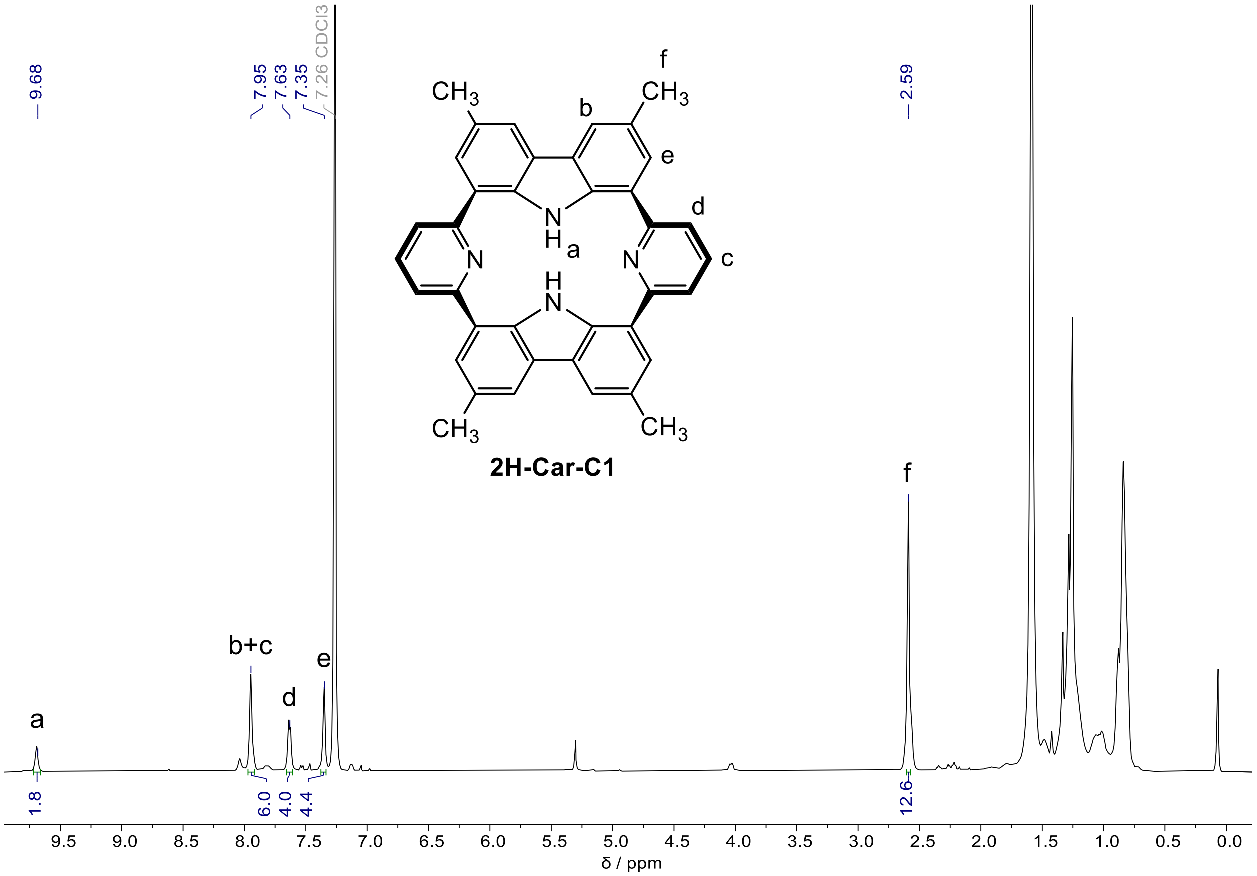

Supplementary Figure 43. ^{1}H NMR of **2H-Car-C1** (500 MHz, 298 K, $CDCl_3$).

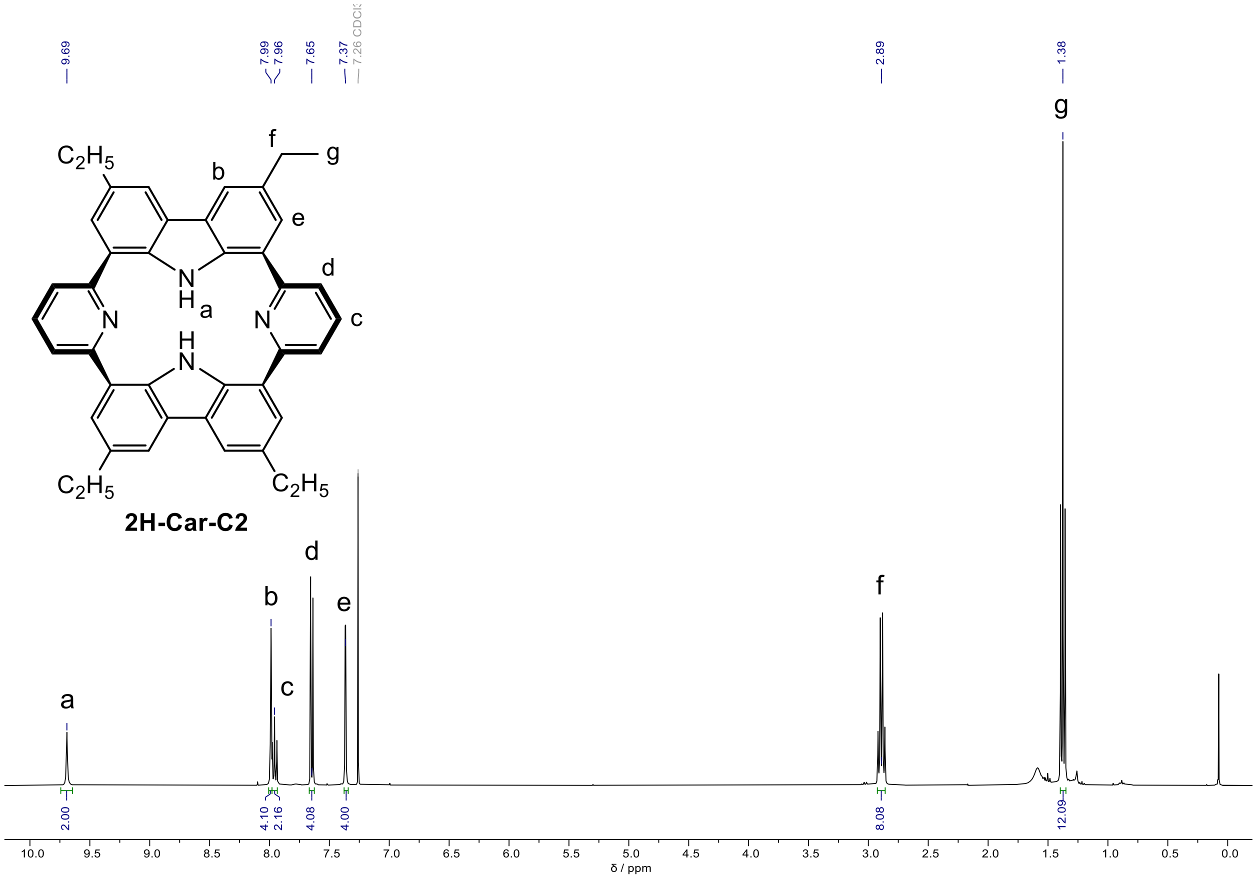

Supplementary Figure 44. ^{1}H NMR of **2H-Car-C2** (400 MHz, 298 K, $CDCl_3$).

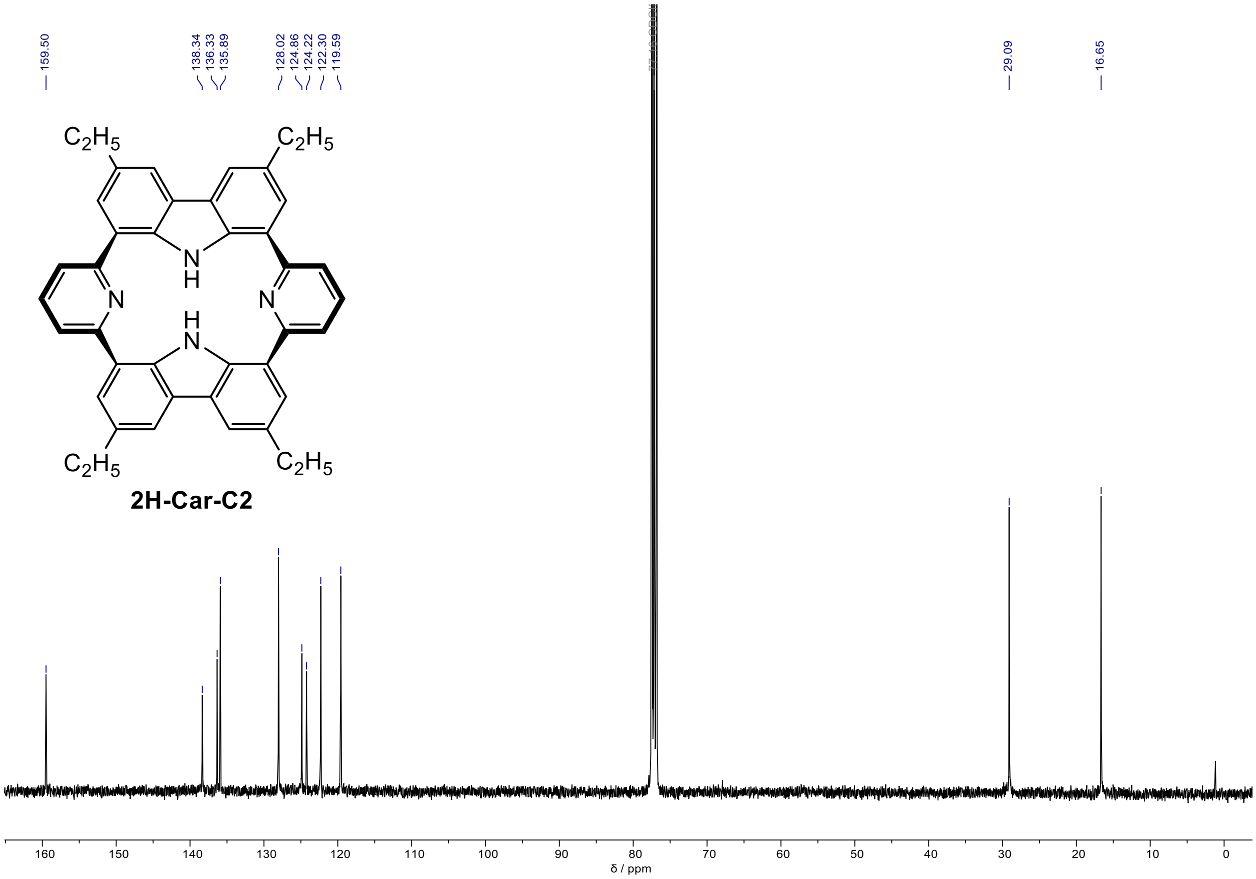

Supplementary Figure 45. ^{13}C NMR of **2H-Car-C2** (101 MHz, 298 K, $CDCl_3$).

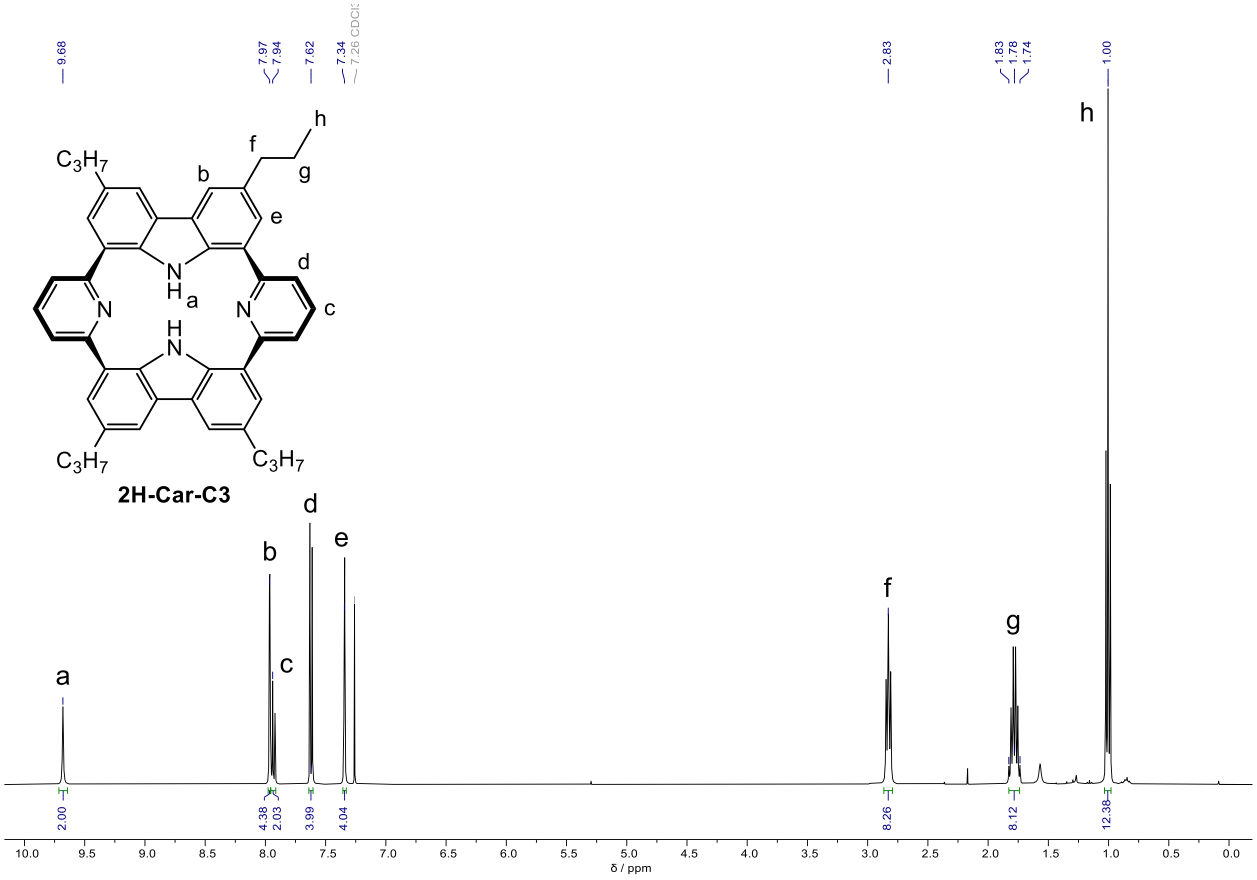

Supplementary Figure 46. ^{1}H NMR of **2H-Car-C3** (400 MHz, 298 K, $CDCl_3$).

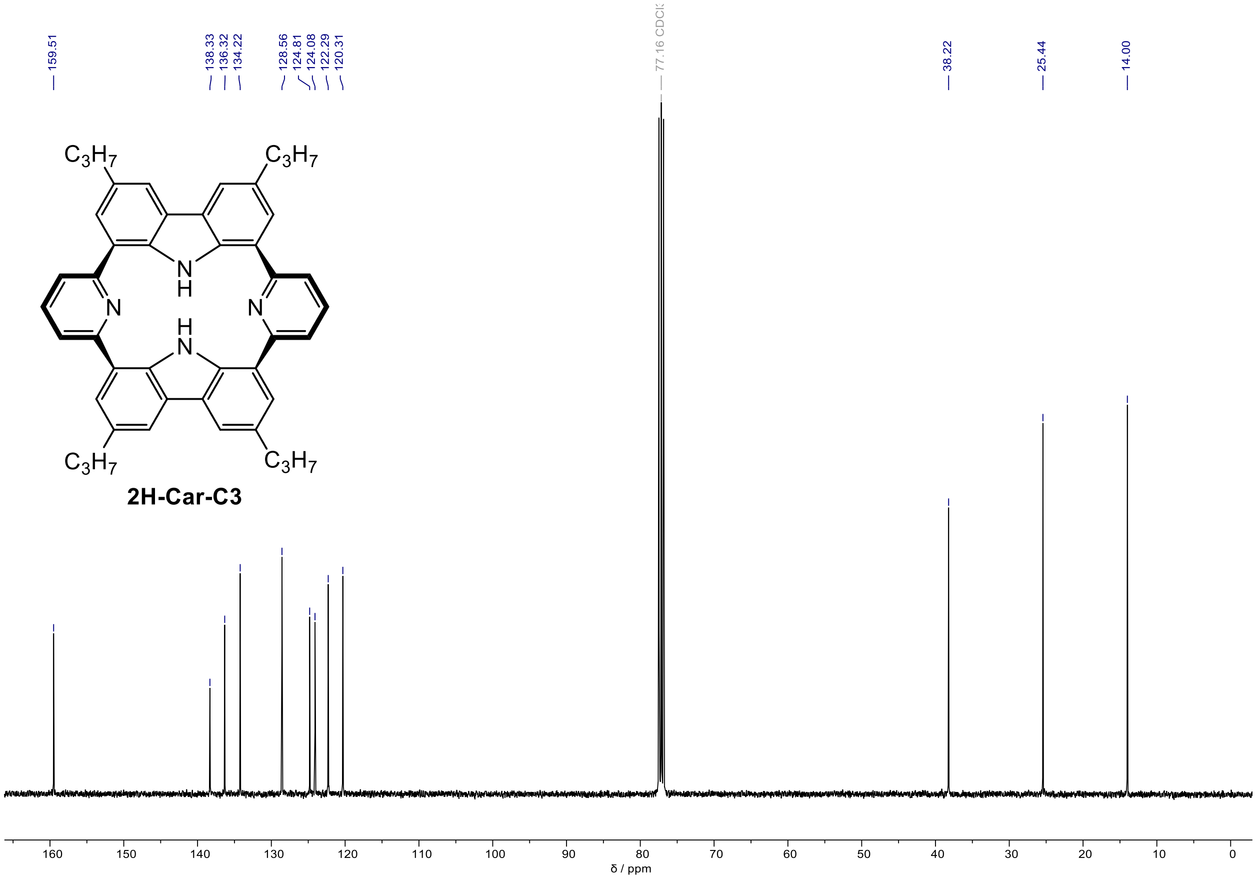

Supplementary Figure 47. ^{13}C NMR of **2H-Car-C3** (101 MHz, 298 K, $CDCl_3$).

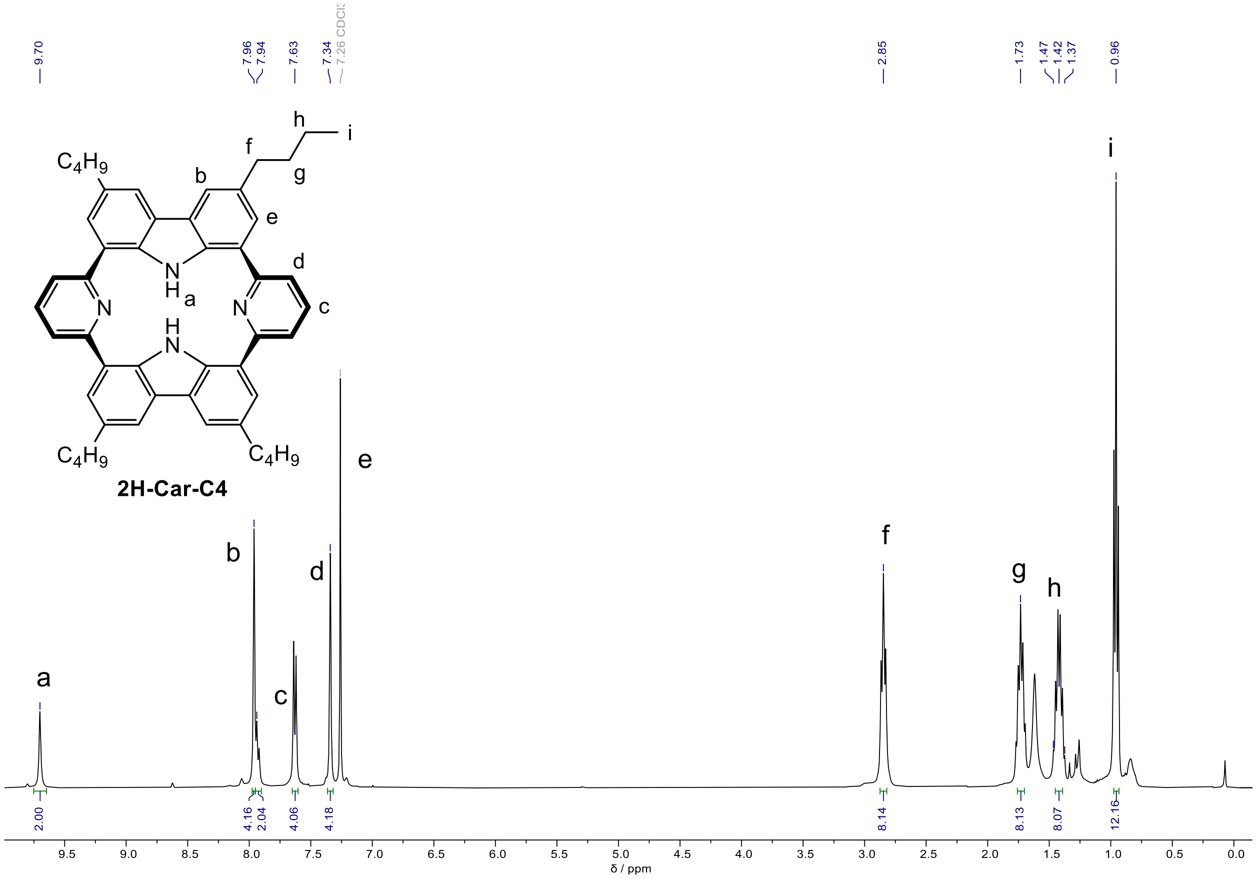

Supplementary Figure 48. ^{1}H NMR of **2H-Car-C4** (400 MHz, 298 K, $CDCl_3$).

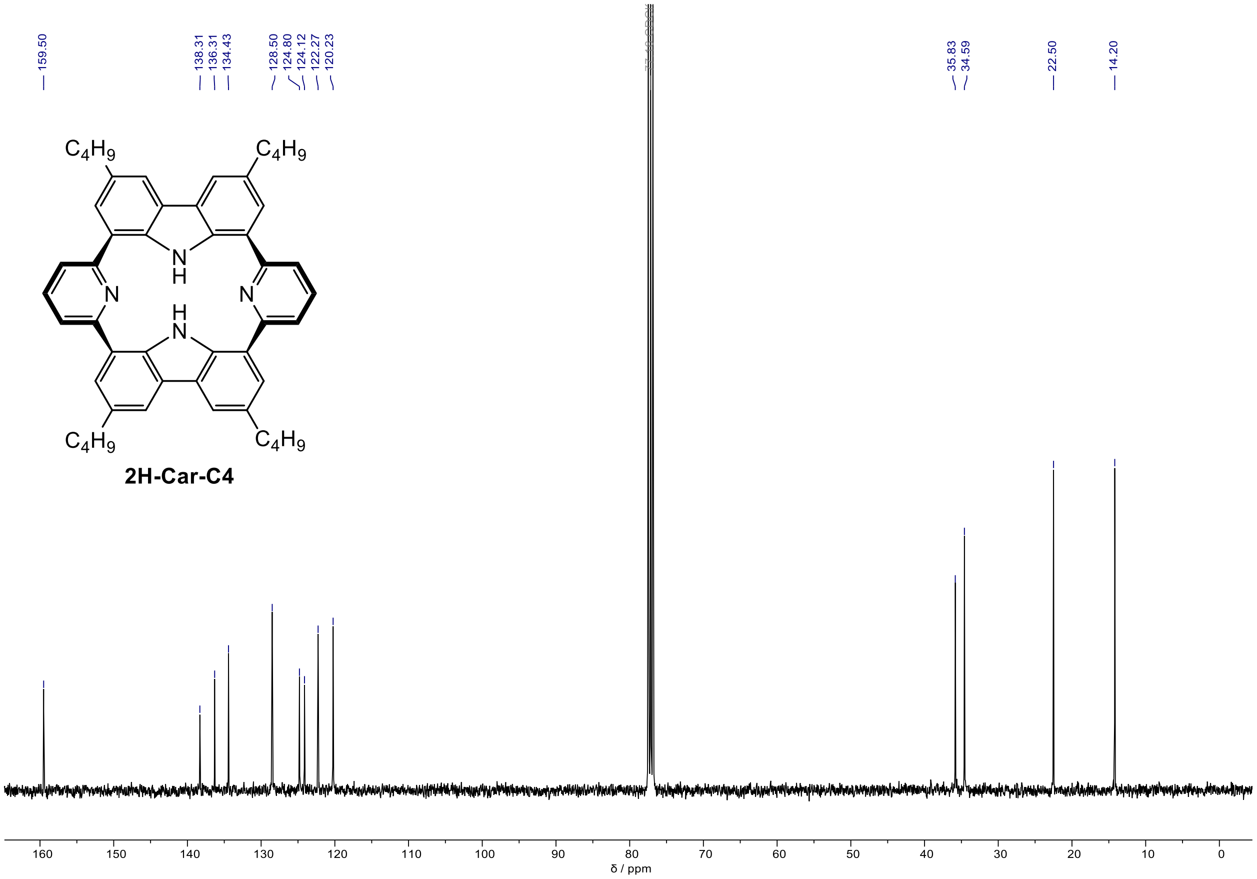

Supplementary Figure 49. ^{13}C NMR of **2H-Car-C4** (101 MHz, 298 K, $CDCl_3$).

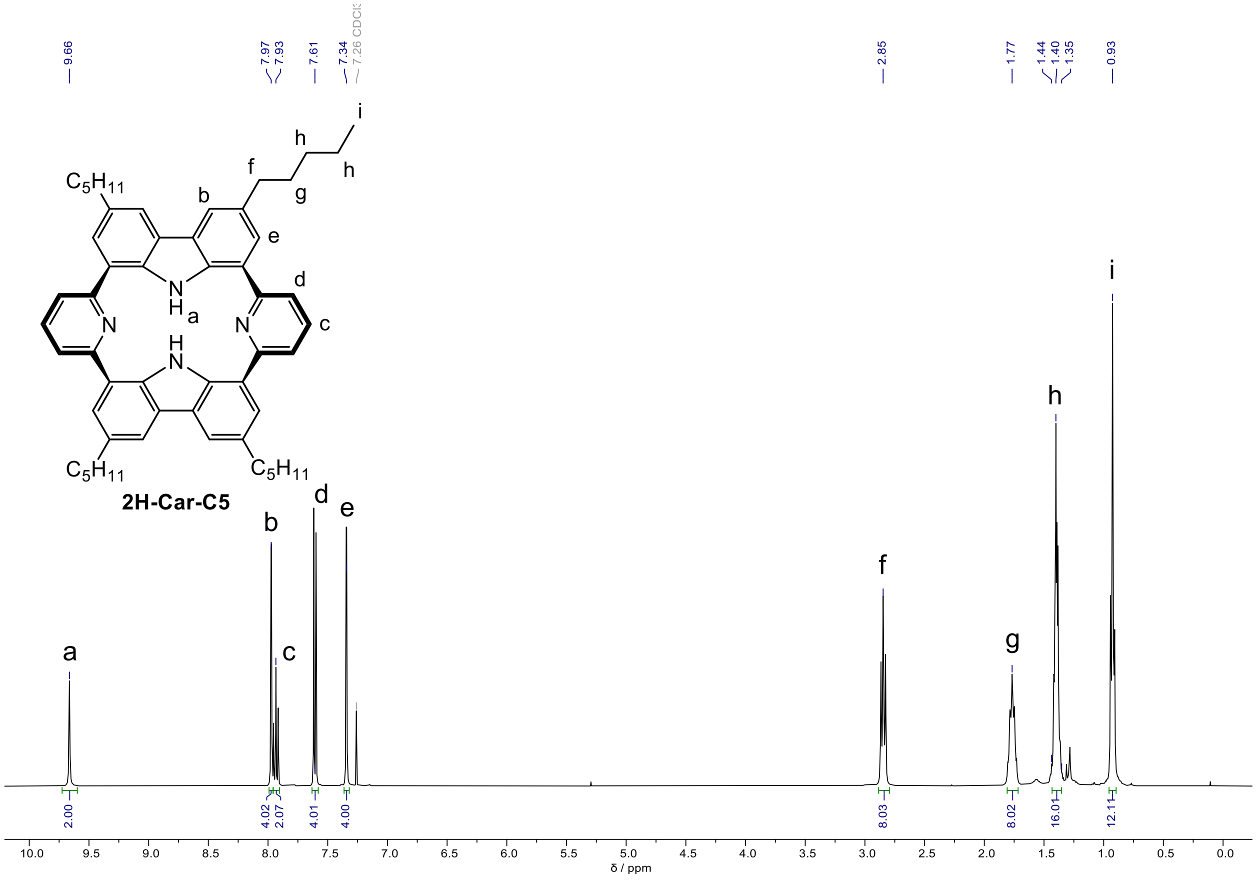

Supplementary Figure 50. ^{1}H NMR of **2H-Car-C5** (400 MHz, 298 K, $CDCl_3$).

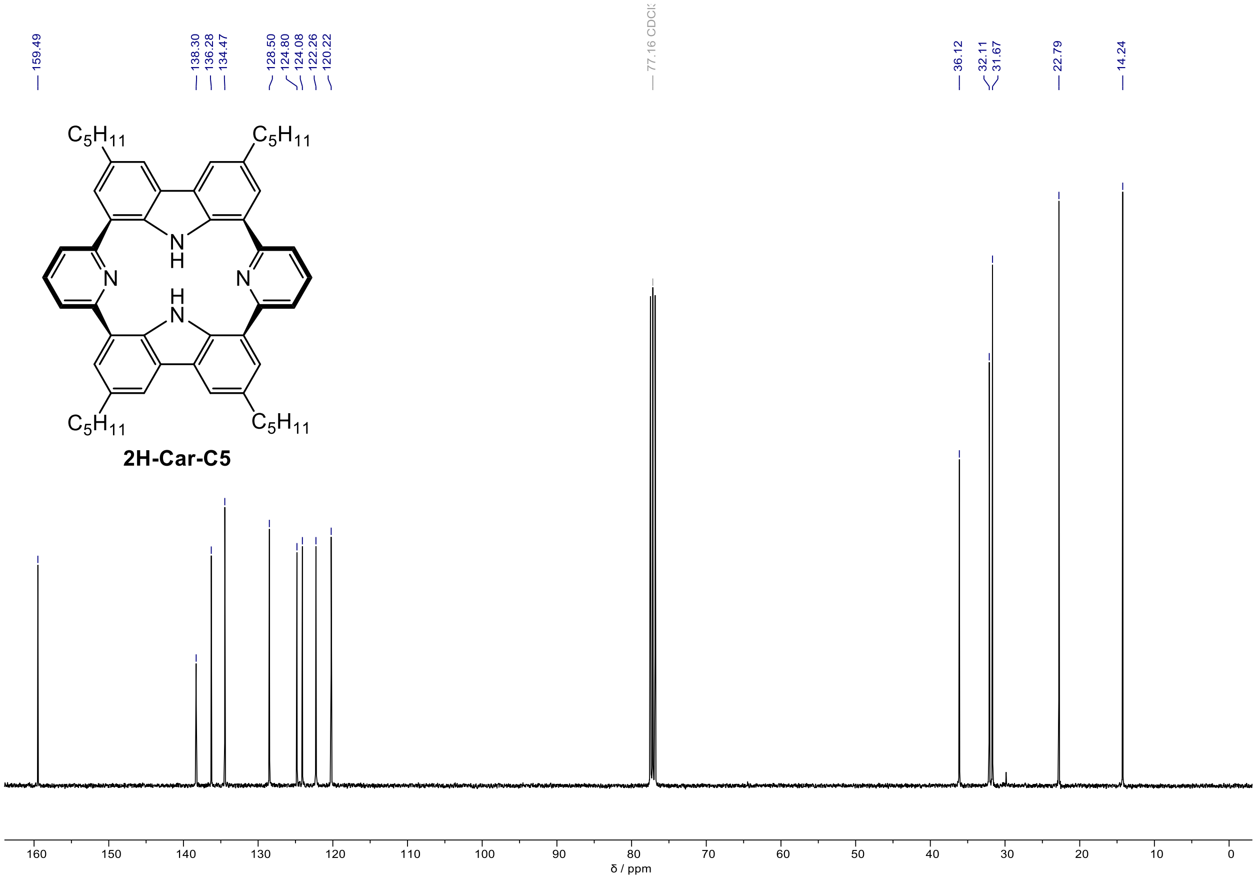

Supplementary Figure 51. ^{13}C NMR of **2H-Car-C5** (101 MHz, 298 K, $CDCl_3$).

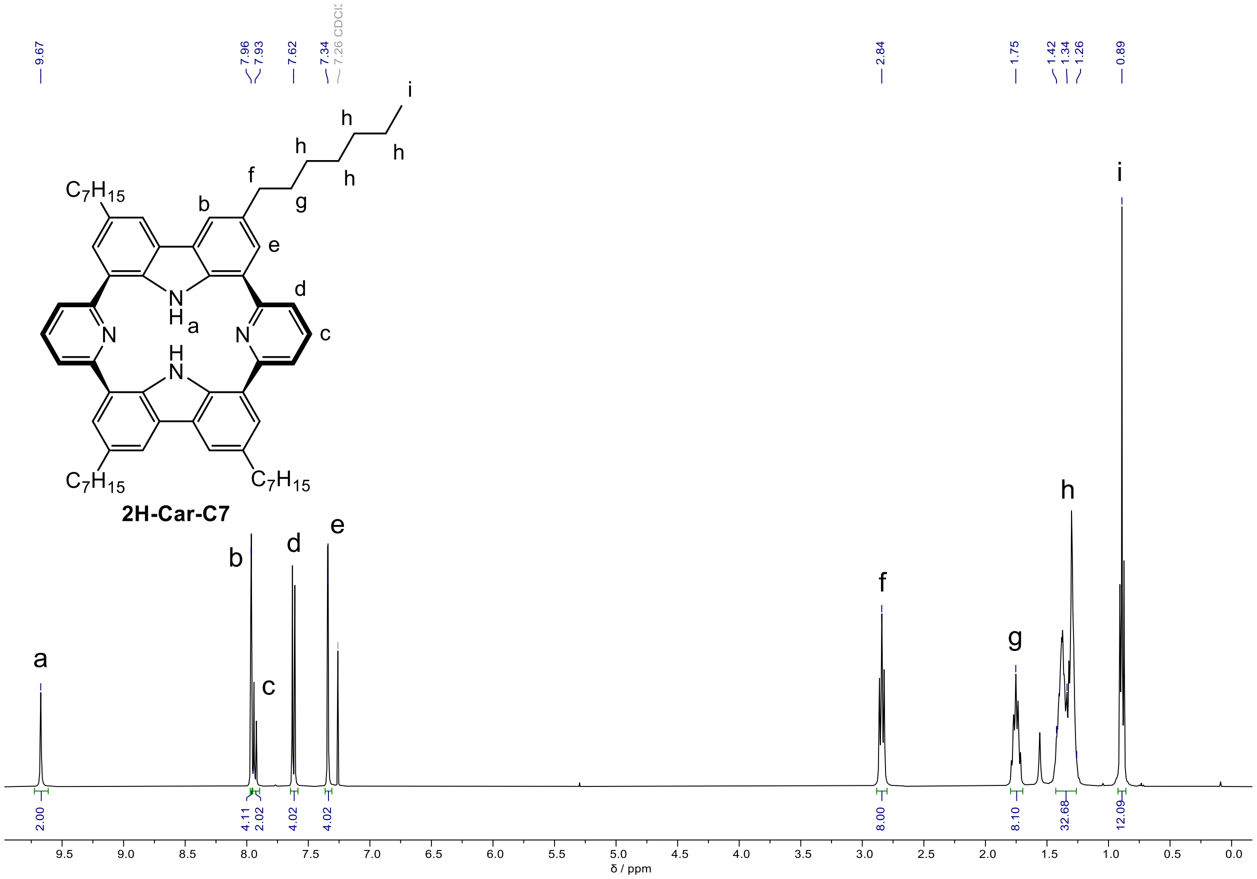

Supplementary Figure 52. ^{1}H NMR of **2H-Car-C7** (400 MHz, 298 K, $CDCl_3$).

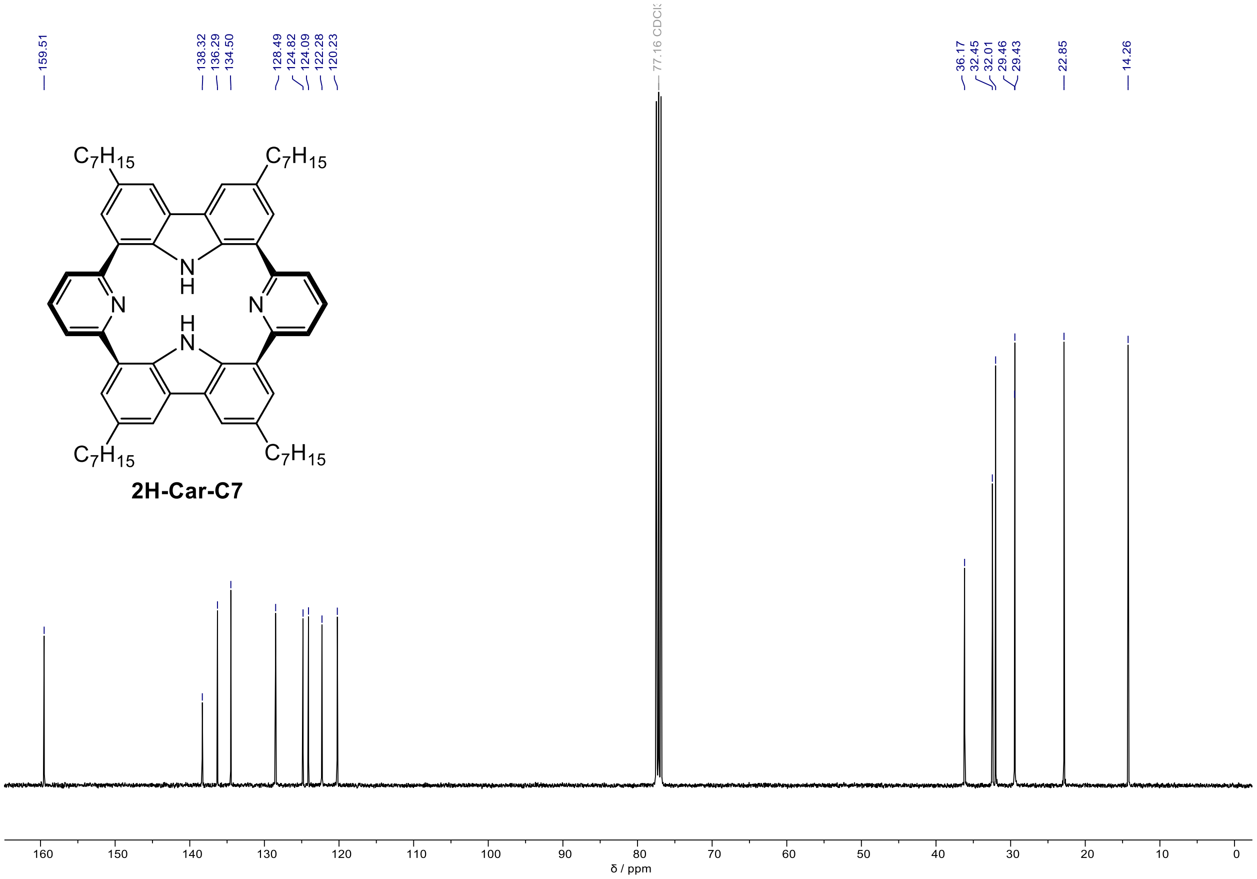

Supplementary Figure 53. ^{13}C NMR of **2H-Car-C7** (101 MHz, 298 K, $CDCl_3$).

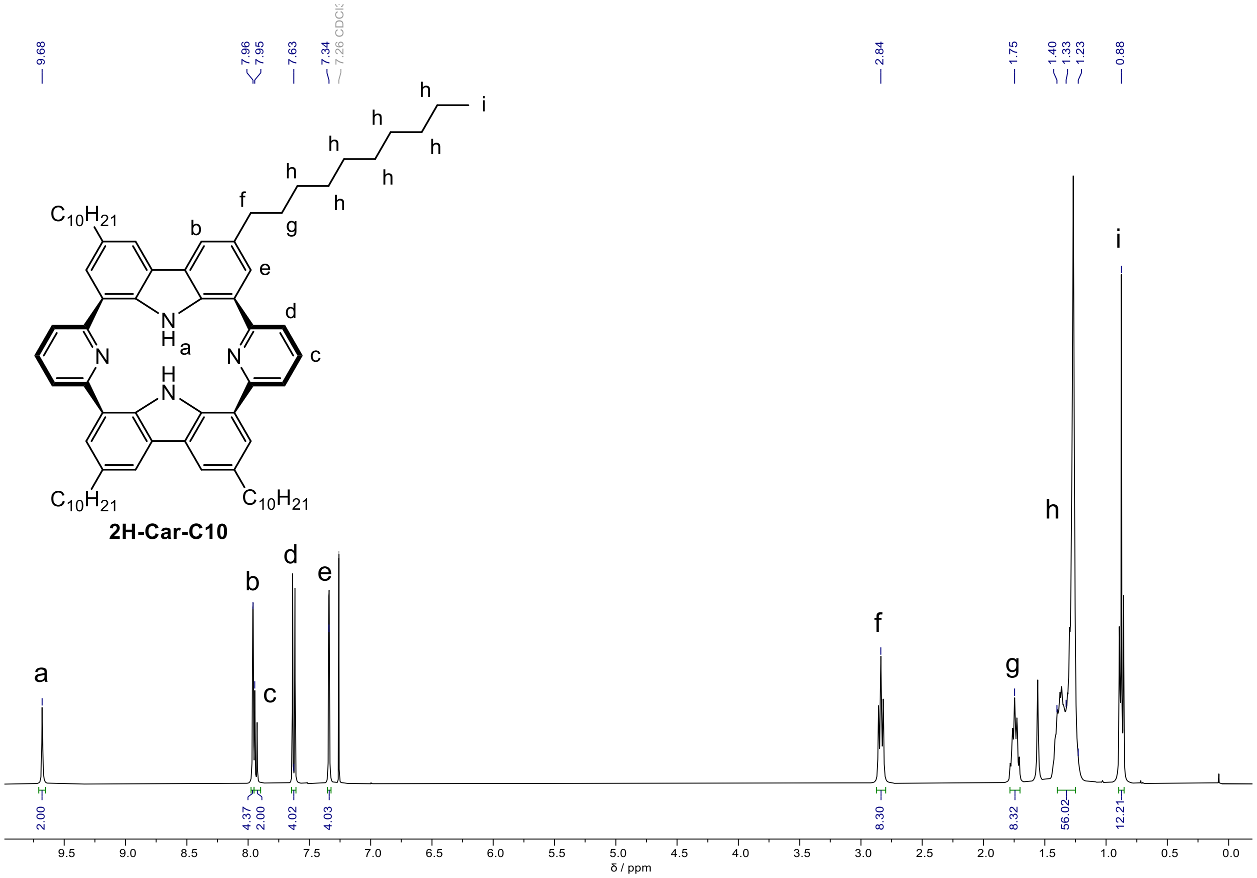

Supplementary Figure 54. ^{1}H NMR of **2H-Car-C10** (400 MHz, 298 K, $CDCl_3$).

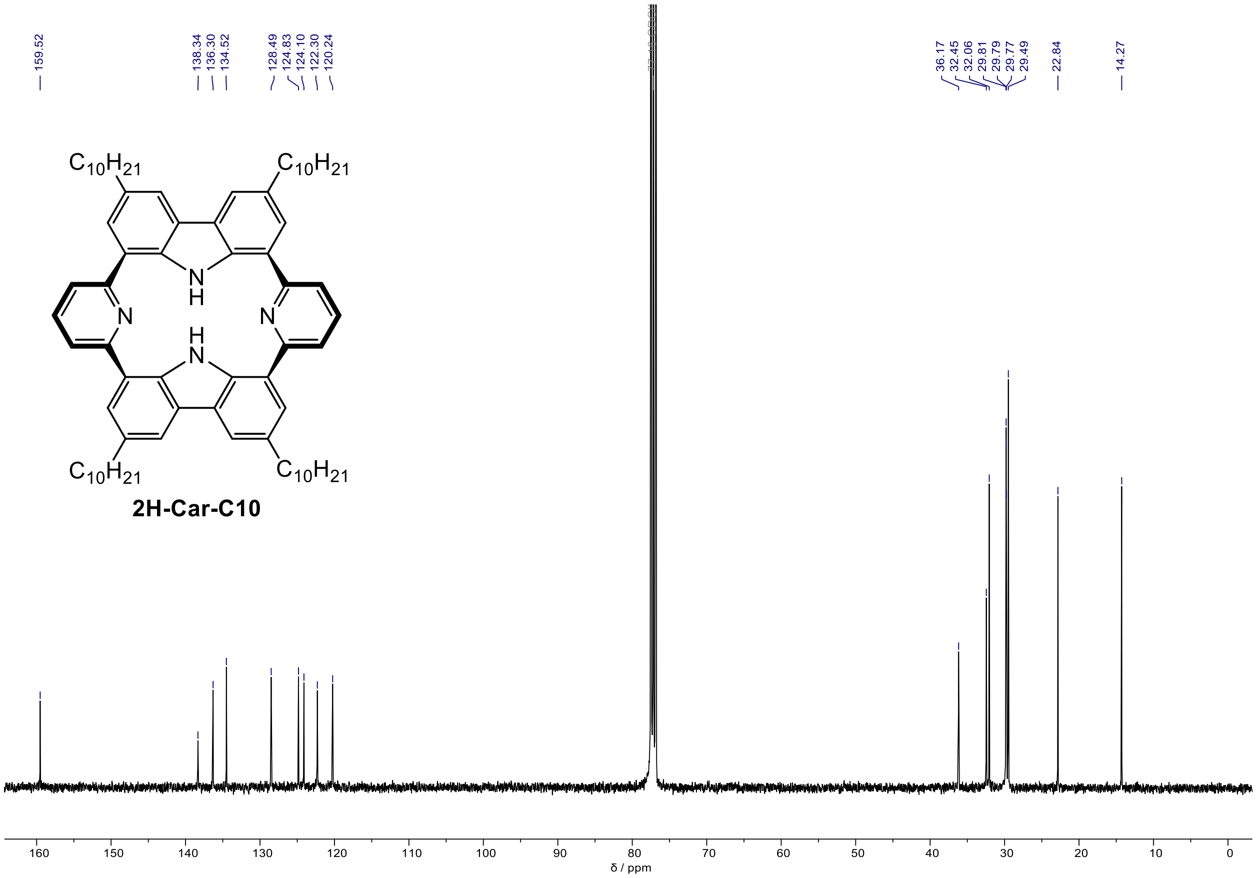

Supplementary Figure 55. ^{13}C NMR of **2H-Car-C10** (101 MHz, 298 K, $CDCl_3$).

Variable Temperature ^{1}H NMR Spectra

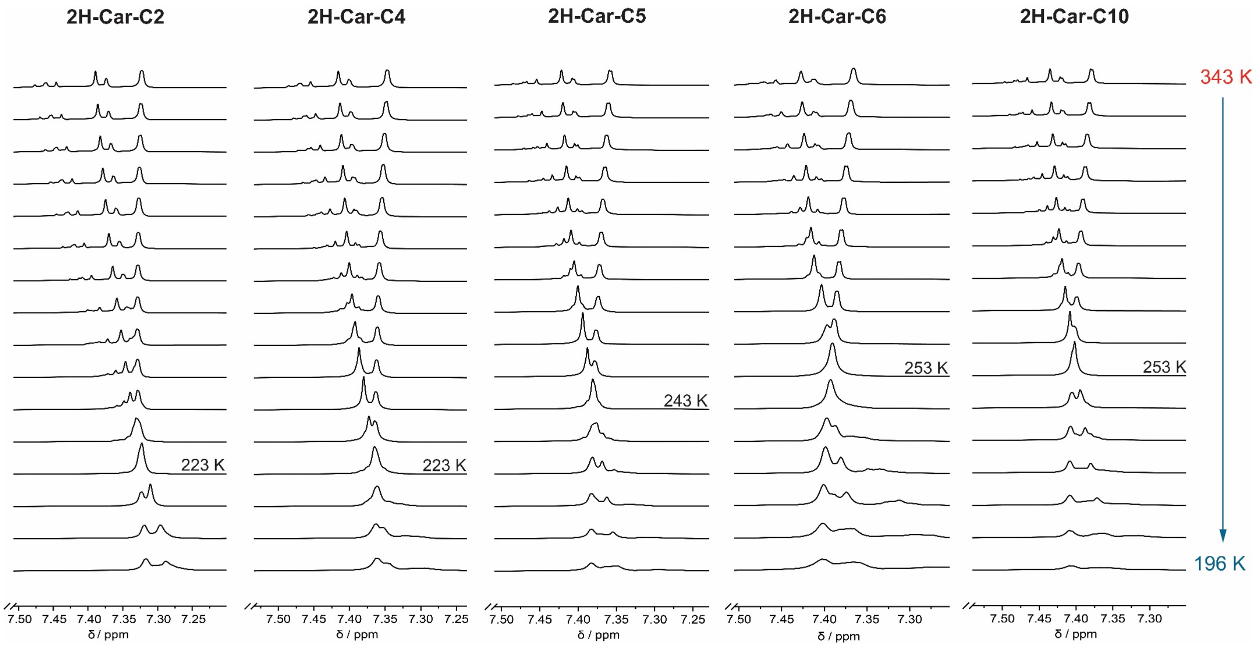

Supplementary Figure 56. Variable temperature ^{1}H NMR spectra of the aromatic region of **2H-Car-C2**, **2H-Car-C4**, **2H-Car-C5**, **2H-Car-C6** and **2H-Car-C10** (500 MHz, 343 → 196 K each 10 K, toluene-d_8). Coalescence of the aromatic signals into a broad singlet is observed as lowering the temperature. **2H-Car-C2** and **2H-Car-C4** coalesce at 223 K, **2H-Car-C5** coalesces at 243 K and **2H-Car-C6** and **2H-Car-C10** coalesce at 253 K. This supports the nature of assemblies from **2H-Car-C2** and **2H-Car-C4** to be similar, whereas **2H-Car-C6** and **2H-Car-C10** have a different nature, with **2H-Car-C5b** being the transitioning point.

Supplementary Table 1. Summary of selected ^{1}H NMR (500 MHz, toluene-d_8, 9.25 mM) chemical shift variation of **2H-Car-C2** with respect to temperature.

2H-Car-C2

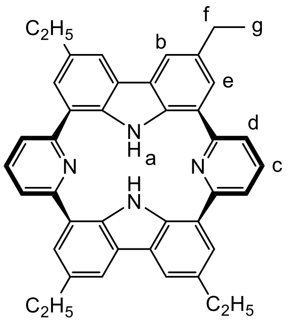

Selected NMR shifts (center of signal) of **2H-Car-C2** toluene-d_8 (δ; Δδ[a]) in ppm

T / K	c	d	e
**343**	7.46; +0.04	7.38; +0.02	7.32; +0.01
**333**	7.45; +0.03	7.38	7.32
**323**	7.45	7.37; +0.01	7.32
**313**	7.44; +0.02	7.37	7.33; 0.00
**303**	7.43; +0.01	7.37	7.33
**293**	7:42; 0.00	7.36; 0.00	7.33
**283**	7.41; −0.01	7.36	7.33
**273**	7.40; −0.02	7.35; −0.01	7.33
**263**	7.39; −0.03	7.34; −0.02	7.33
**253**	7.37; **−0.05**	7.34[c]	7.33[c]
**243**	7.36[c]; **−0.06**	7.33[c]; −0.03	7.33[c]
**233**	7.35[c]; **−0.07**	7.33[c]	7.33[c]
**223**	7.32; **−0.10**	7.32; −0.04	7.32; −0.01
**213**	7.31[b,c]; **−0.11**	7.31[b,c]; **−0.05**	7.32[b,c]
**203**	7.28[b,c]; **−0.14**	7.30[b,c]; **−0.06**	7.32[b,c]
**196**	7.27[b,c]; **−0.15**	7.29[b,c]; **−0.07**	7.32[b,c]

[a] Δδ referenced to 293 K, [b] broad signal, [c] assignment unclear.

Supplementary Table 2. Summary of selected ^{1}H NMR (500 MHz, toluene-d_8, 9.25 mM) chemical shift variation of **2H-Car-C4** with respect to temperature.

2H-Car-C4

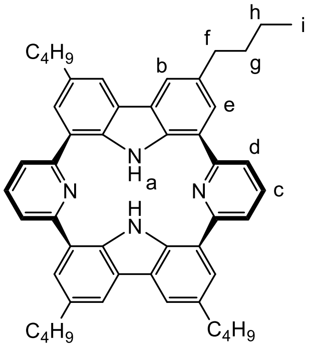

Selected NMR shifts (center of signal) of **2H-Car-C4** toluene-d_8 (δ; Δδ[a]) in ppm

T / K	c	d	e
**343**	7.47; +0.04	7.41; +0.01	7.35; −0.01
**333**	7.46; +0.03	7.40; 0.00	7.35
**323**	7.46	7.40	7.35
**313**	7.45; +0.02	7.40	7.35
**303**	7.44; +0.01	7.40	7.35
**293**	7.43; 0.00	7.40	7.36; 0.00
**283**	7.43	7.39; −0.01	7.36
**273**	7.42; −0.01	7.39	7.36
**263**	7.41[c]; −0.02	7.39[c]	7.36[c]
**253**	7.39[c]; −0.04	7.39[c]	7.36[c]
**243**	7.38[c]; **−0.05**	7.38[c]; −0.02	7.36[c]
**233**	7.37[c]; **−0.06**	7.37[c]; −0.03	7.36[c]
**223**	7.36; **−0.07**	7.36; −0.04	7.36
**213**	7.34[b,c]; **−0.09**	7.36[b,c]	7.36[b,c]
**203**	7.31[b,c]; **−0.12**	7.35[b,c]; **−0.05**	7.36[b,c]
**196**	7.29[b,c]; **−0.14**	7.34[b,c]; **−0.06**	7.35[b,c]; −0.01

[a] Δδ referenced to 293 K, [b] broad signal, [c] assignment unclear.

Supplementary Table 3. Summary of selected ^{1}H NMR (500 MHz, toluene-d_8, 9.25 mM) chemical shift variation of **2H-Car-C5** with respect to temperature.

2H-Car-C5

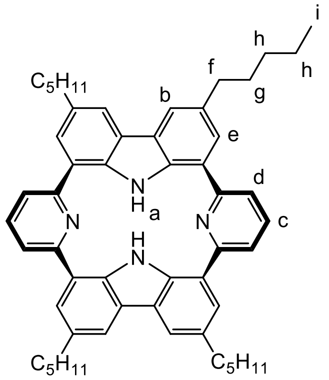

Selected NMR shifts (center of signal) of **2H-Car-C5** toluene-d_8 (δ; Δδ[a]) in ppm

T / K	c	d	e
**343**	7.47; +0.04	7.41; +0.01	7.36; −0.01
**333**	7.46; +0.03	7.41	7.36
**323**	7.46	7.41	7.36
**313**	7.45; +0.02	7.41	7.36
**303**	7.44; +0.01	7.41	7.37; 0.00
**293**	7.43; 0.00	7.40; 0.00	7.37
**283**	7.42; −0.01	7.40	7.37
**273**	7.41[c]; −0.02	7.40[c]	7.37
**263**	7.39[b,c]; −0.04	7.39[b,c]; −0.01	7.38[b]; +0.01
**253**	7.39[b,c]; −0.02	7.39[b,c]	7.38[b]
**243**	7.38[b]; −0.03	7.38[b]; −0.02	7.38[b]
**233**	7.36[b,c]; **−0.05**	7.37[b,c]; −0.03	7.38[b]
**223**	7.34[b,c]; **−0.07**	7.37[b,c]	7.38[b,c]
**213**	7.32[b,c]; **−0.09**	7.36[b,c]; −0.04	7.38[b,c]
**203**	7.30[b,c]; **−0.11**	7.36[b,c]	7.38[b,c]
**196**	7.28[b,c]; **−0.13**	7.36[b,c]	7.38[b,c]

[a] Δδ referenced to 293 K, [b] broad signal, [c] assignment unclear.

Supplementary Table 4. Summary of selected ^1H NMR (500 MHz, toluene-d_8, 9.25 mM) chemical shift variation of **2H-Car-C6** with respect to temperature.

2H-Car-C6

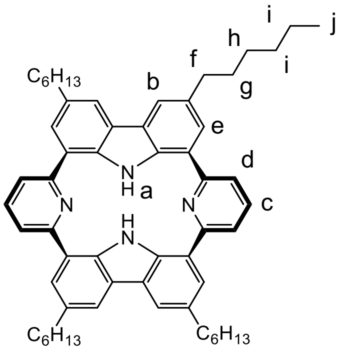

Selected NMR shifts (center of signal) of **2H-Car-C6** in toluene-d_8 (δ; Δδ[a]) in ppm

T / K	c	d	e
**343**	7.47; +0.05	7.42; +0.01	7.37[b]; −0.01
**333**	7.46; +0.04	7.42	7.37
**323**	7.46	7.42	7.37
**313**	7.45; +0.03	7.41; 0.00	7.37
**303**	7.44; +0.02	7.41	7.38; 0.00
**293**	7.42; 0.00	7.41	7.38
**283**	7.41[c]; −0.01	7.41[c]	7.38
**273**	7.40[b,c]; −0.02	7.40[b,c]; −0.01	7.39; +0.01
**263**	7.40[b,c]	7.40[b,c]	7.39[b]
**253**	7.39[b,c]; −0.03	7.39[b,c]; −0.02	7.39[b,c]
**243**	7.39[b,c]	7.39[b,c]	7.39[b,c]
**233**	7.35[b,c]; −0.05	7.39[b,c]	7.40[b,c]; +0.02
**223**	7.33[b,c]; **−0.09**	7.39[b,c]	7.40[b,c]
**213**	7.31[b,c]; **−0.11**	7.38[b,c]; −0.03	7.40[b,c]
**203**	7.29[b,c]; **−0.13**	7.38[b,c]	7.40[b,c]
**196**	7.27[b,c]; **−0.15**	7.36[b,c]; −0.05	7.40[b,c]

[a] Δδ referenced to 293 K, [b] broad signal, [c] assignment unclear.

Supplementary Table 5. Summary of selected ^{1}H NMR (500 MHz, toluene-d_8, 9.25 mM) chemical shift variation of **2H-Car-C10** with respect to temperature.

2H-Car-C10

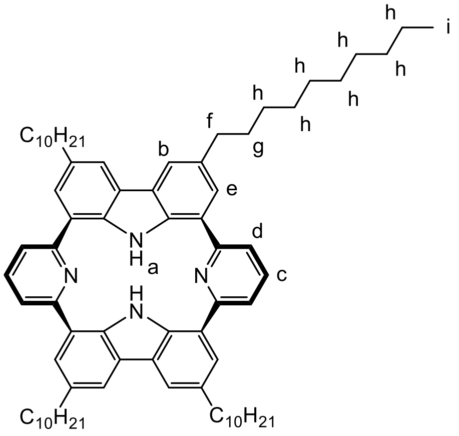

Selected NMR shifts (center of signal) of **2H-Car-C10** toluene-d_8 (δ; Δδ[a]) in ppm

T / K	c	d	e
**343**	7.48; +0.04	7.43; +0.01	7.38; −0.01
**333**	7.47; +0.03	7.43	7.38
**323**	7.47	7.42; 0.00	7.38
**313**	7.46; +0.02	7.42	7.39; 0.00
**303**	7.45; +0.01	7.42	7.39
**293**	7.44; 0.00	7.42	7.39
**283**	7.43; −0.01	7.41; −0.01	7.40; +0.01
**273**	7.41[b,c]; −0.03	7.41[b,c]	7.40[b]
**263**	7.41[b,c]	7.41[b,c]	7.40[b,c]
**253**	7.40; −0.04	7.40; −0.02	7.40
**243**	7.38[b,c]; **−0.06**	7.40[b,c]	7.41[b,c]; +0.02
**233**	7.36[b,c]; **−0.08**	7.39[b,c]; −0.03	7.41[b,c]
**223**	7.35[b,c]; **−0.09**	7.39[b,c]	7.41[b,c]
**213**	7.33[b,c]; **−0.11**	7.38[b,c]; −0.04	7.41[b,c]
**203**	7.31[b,c]; **−0.13**	7.37[b,c]; **−0.05**	7.41[b,c]
**196**	7.30[b,c]; **−0.14**	7.36[b,c]; **−0.06**	7.41[b,c]

[a] Δδ referenced to 293 K, [b] broad signal, [c] assignment unclear.

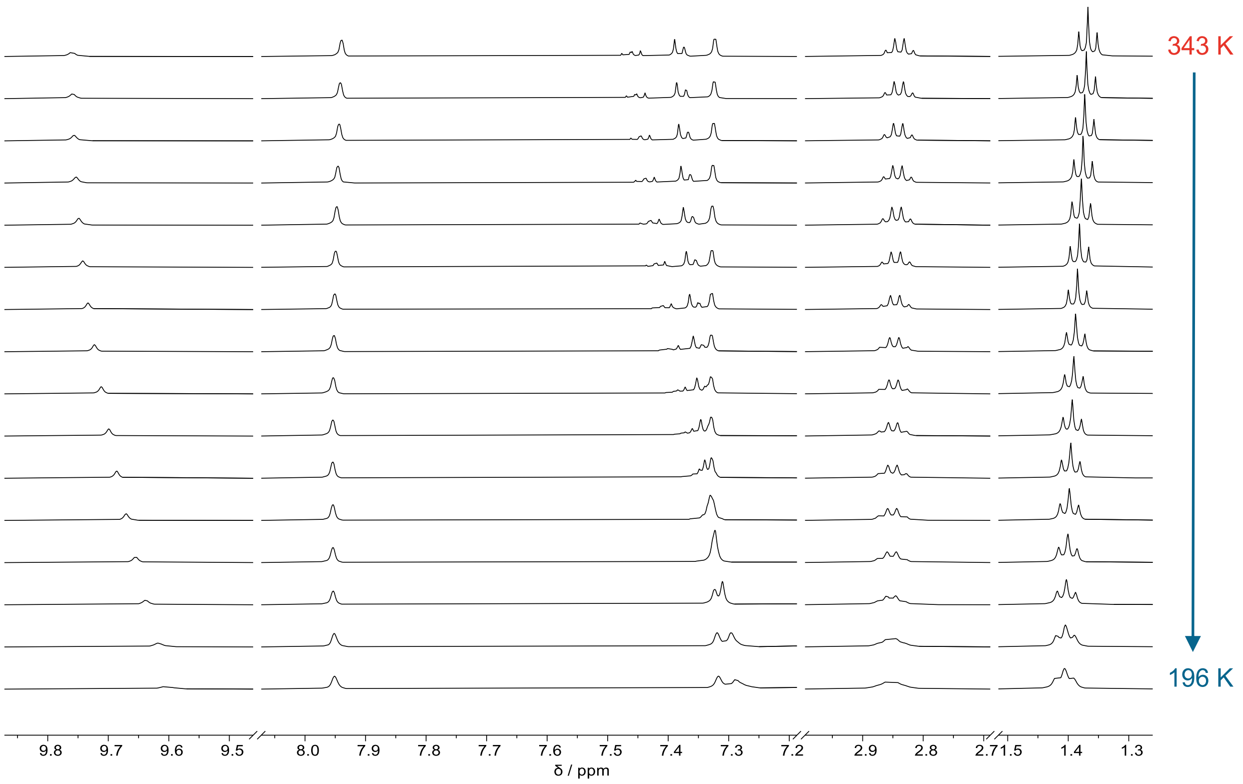

Supplementary Figure 57. Variable temperature ^{1}H NMR spectra of **2H-Car-C2** (500 MHz, 343 → 196 K each 10 K, toluene-d$_8$, 9.25 mM). Peak broadening is observed as the temperature is decreased.

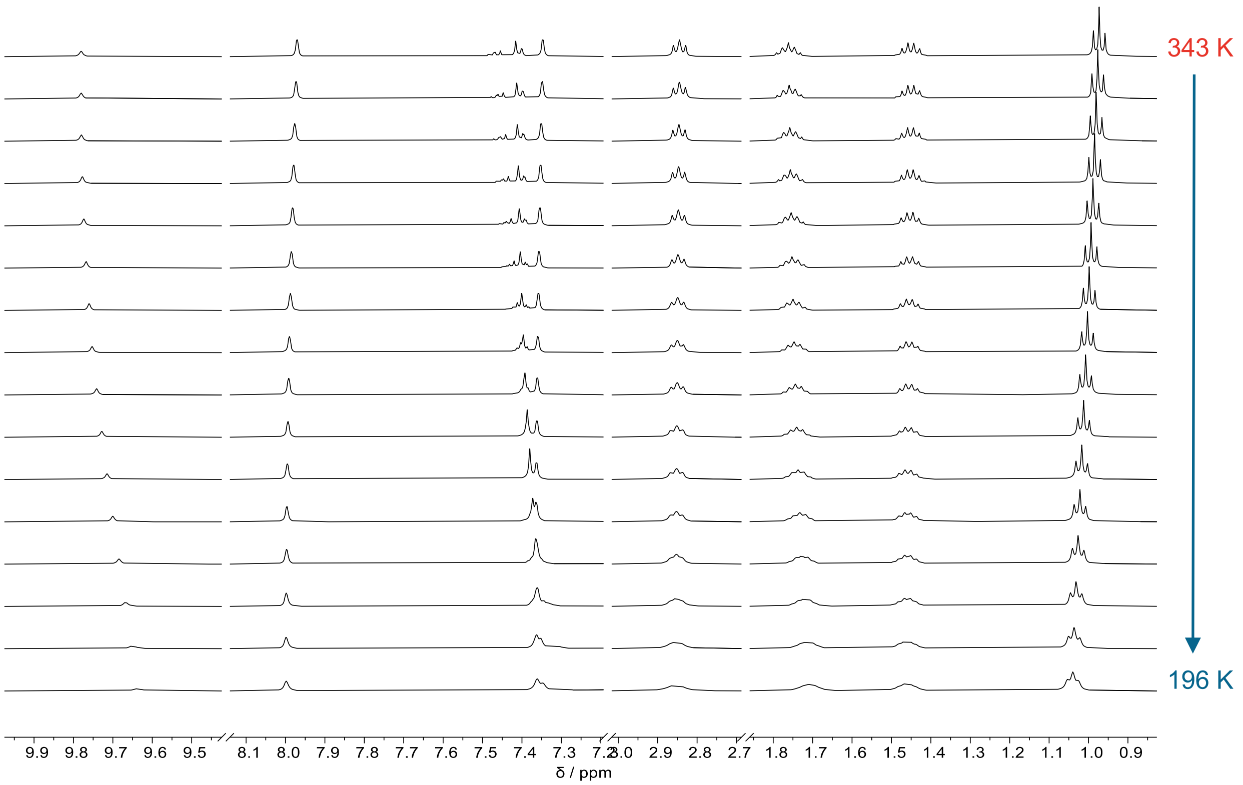

Supplementary Figure 58. Variable temperature ^{1}H NMR spectra of **2H-Car-C4** (500 MHz, 343 → 196 K each 10 K, toluene-d_8, 9.25 mM). Peak broadening is observed as the temperature is decreased.

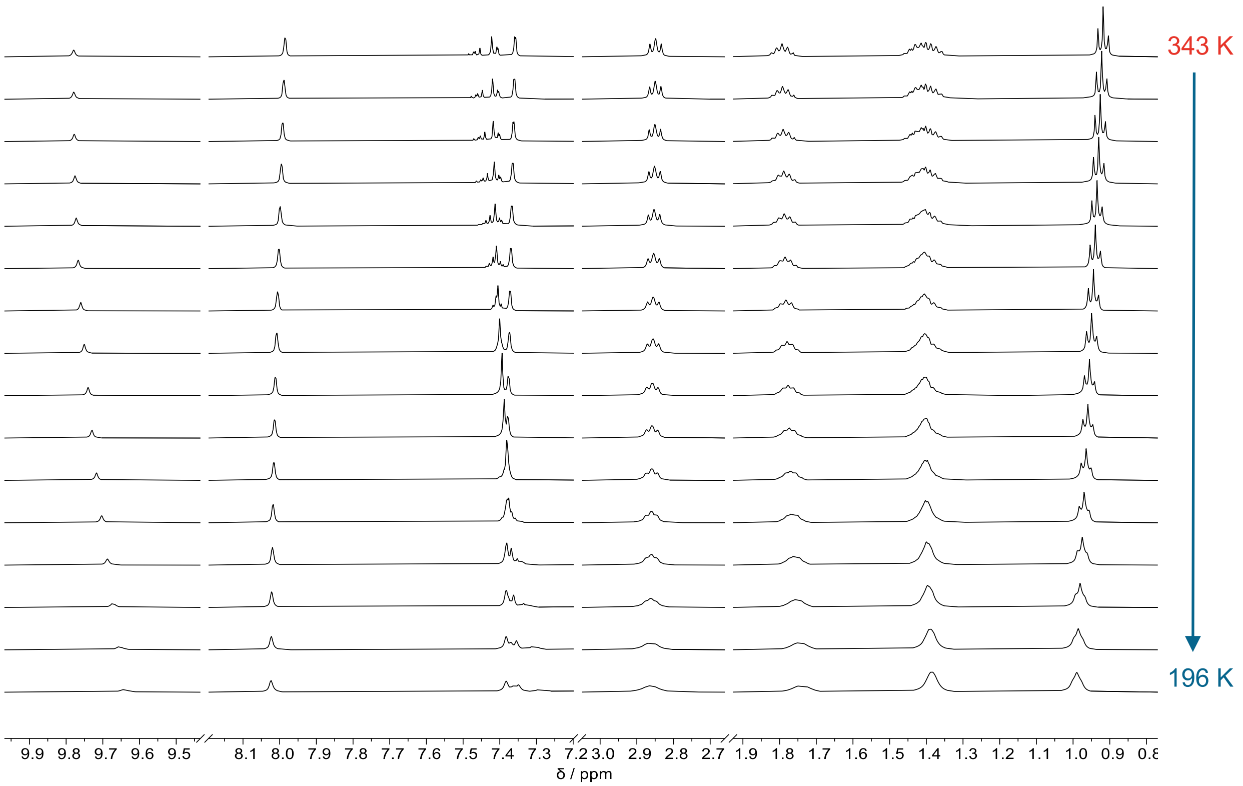

Supplementary Figure 59. Variable temperature ^{1}H NMR spectra of **2H-Car-C5** (500 MHz, 343 → 196 K each 10 K, toluene-d$_8$, 9.25 mM). Peak broadening is observed as the temperature is decreased.

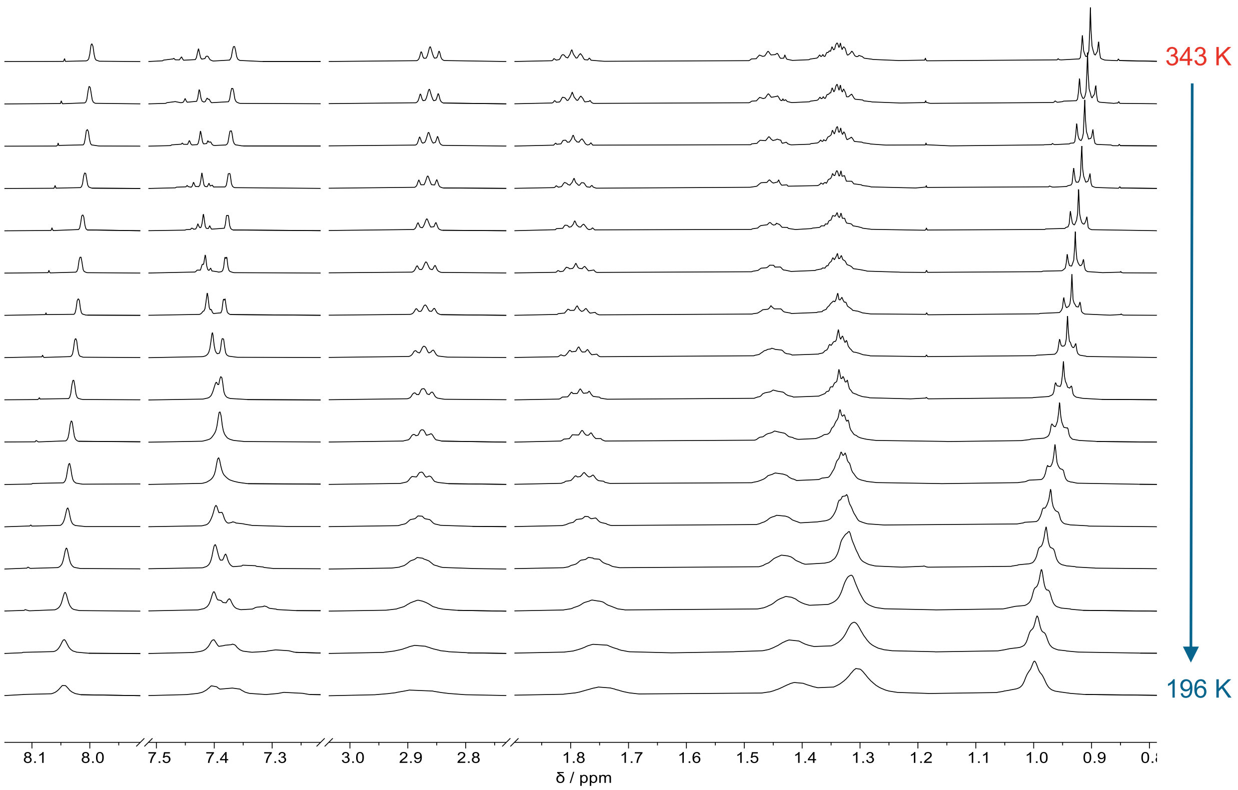

Supplementary Figure 60. Variable temperature ^{1}H NMR spectra of **2H-Car-C6** (500 MHz, 343 → 196 K each 10 K, toluene-d_8, 9.25 mM). Peak broadening is observed as the temperature is decreased.

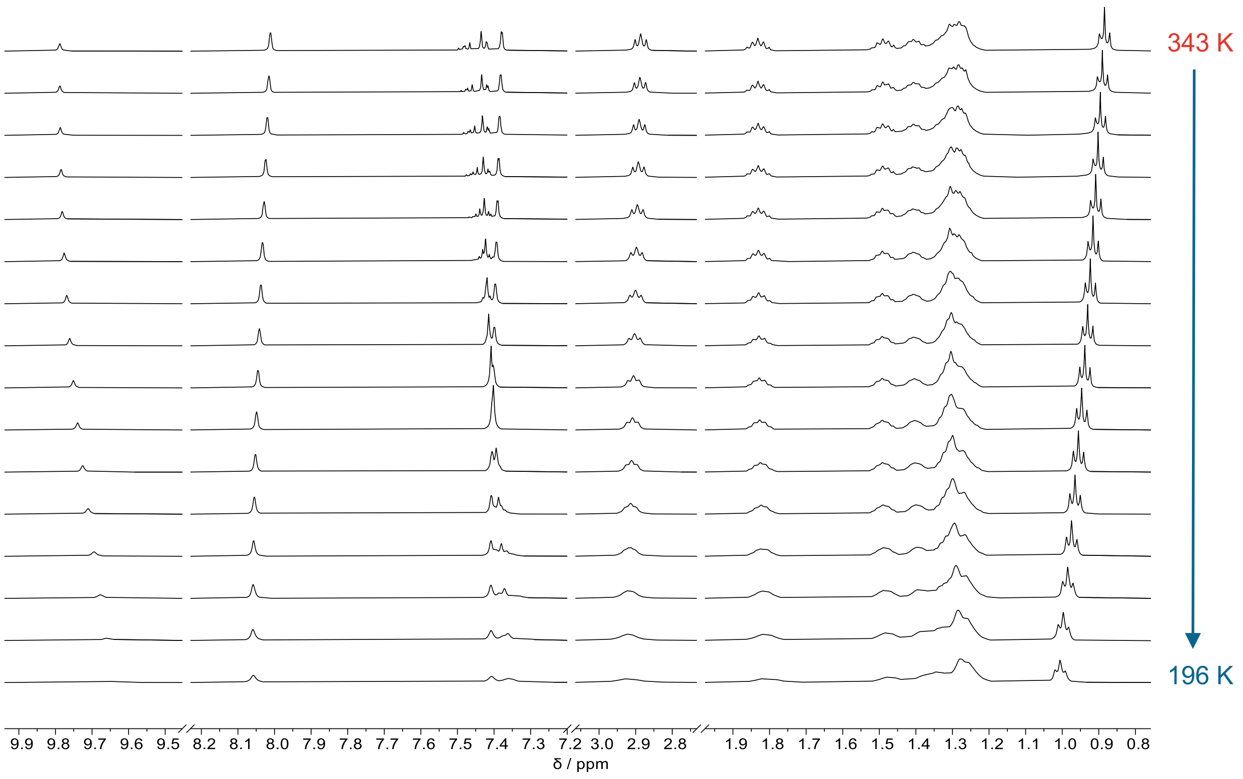

Supplementary Figure 61. Variable temperature ^{1}H NMR spectra of **2H-Car-C10** (500 MHz, 343 → 196 K each 10 K, toluene-d_8, 9.25 mM). Peak broadening is observed as the temperature is decreased.

Spectroscopic Data

Supplementary Table 6. Summary of UV-vis absorption maxima with corresponding logarithmic extinction coefficients, excitation wavelengths, fluorescence emission maxima and associated quantum yields. All measurements were made in toluene at 298 K.

	$\lambda_{abs,max}$ / nm	log ε^{a}	λ_{ex} / nm	$\lambda_{em,max}$ / nm	Φ_f^{b} / %
**2H-Car-C1**	315, 376	4.44, 4.21	315	390	48.7
**2H-Car-C2**	313, 375	4.61, 4.32	313	389	46.7
**2H-Car-C3**	313, 375	4.67, 4.38	313	389	49.0
**2H-Car-C4**	312, 375	4.63, 4.28	312	390	49.1
**2H-Car-C5**	313, 375	4.62, 4.28	313	390	50.3
**2H-Car-C6**	314, 376	4.46, 4.17	314	390	40.5
**2H-Car-C7**	314, 376	4.61, 4.30	314	390	50.2
**2H-Car-C8**	314, 376	4.65, 4.32	314	390	47.3
**2H-Car-C10**	314, 375	4.61, 4.31	314	390	55.9

[a] ε given in $M^{-1}cm^{-1}$, [b] integrating sphere.

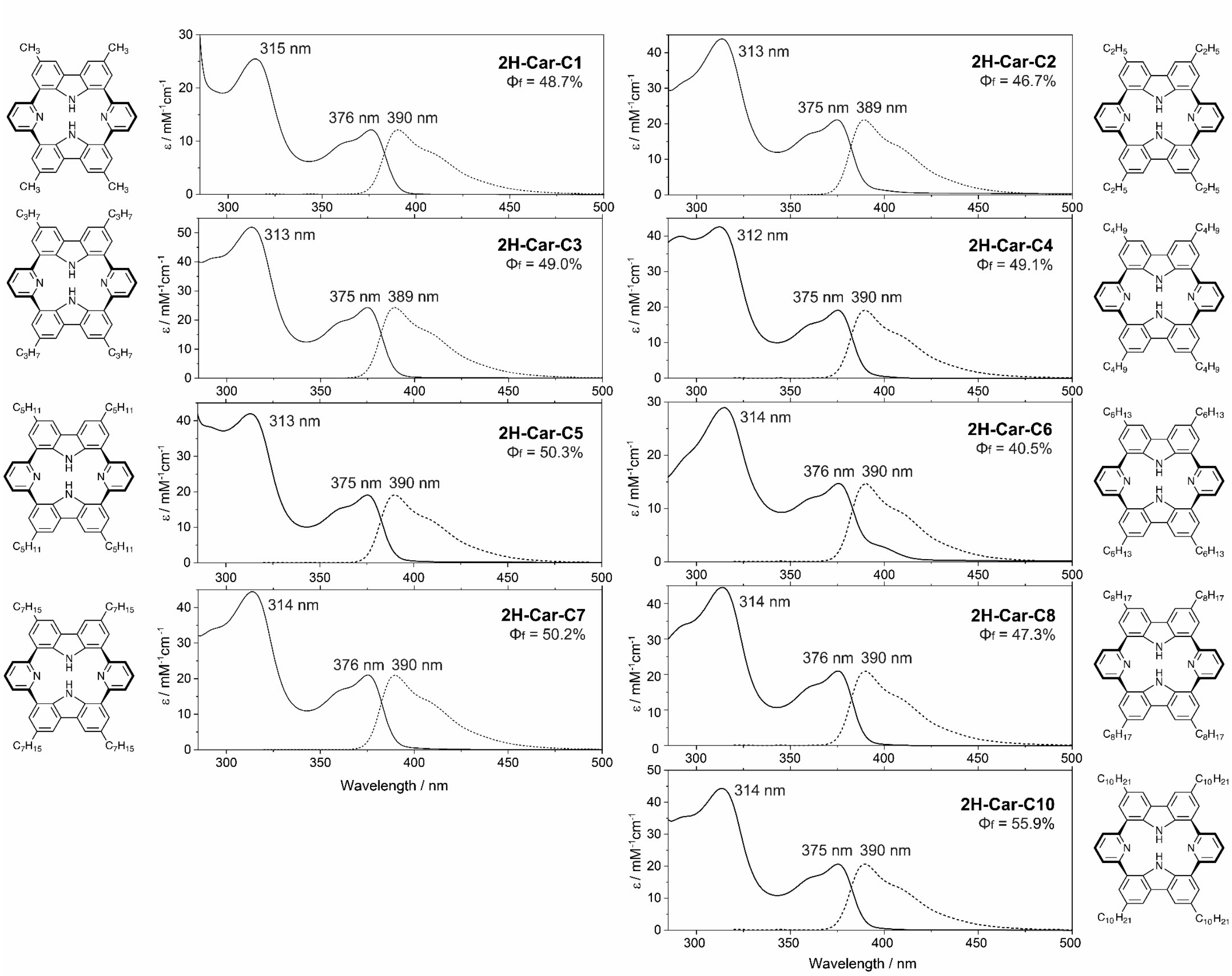

Supplementary Figure 62. Steady-state UV-vis absorption (continuous lines) and fluorescence emission (dashed lines) spectra of indicated carpyridines. All measurements were made in toluene at 298 K.

Microscopy

Additional TEM images

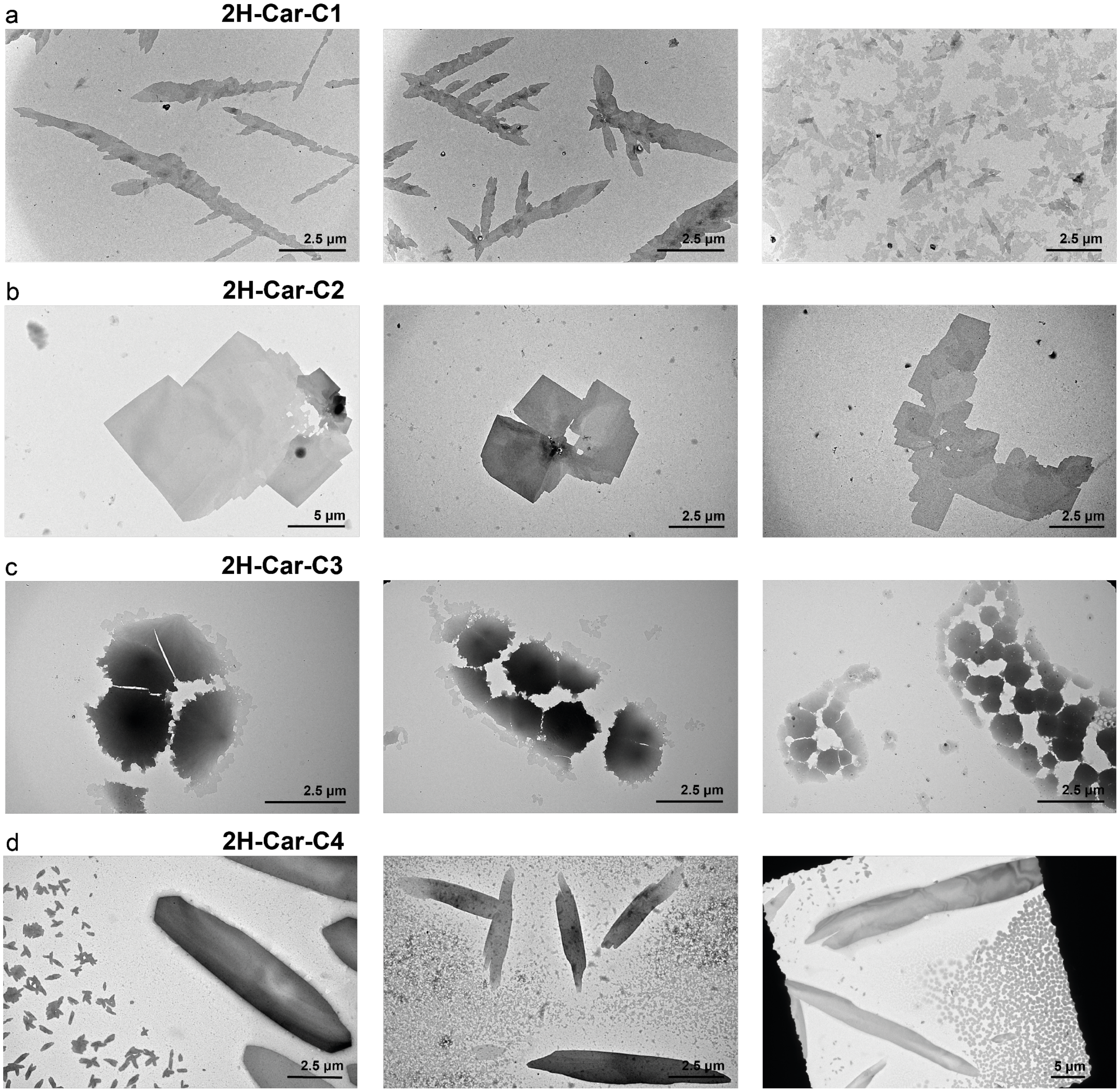

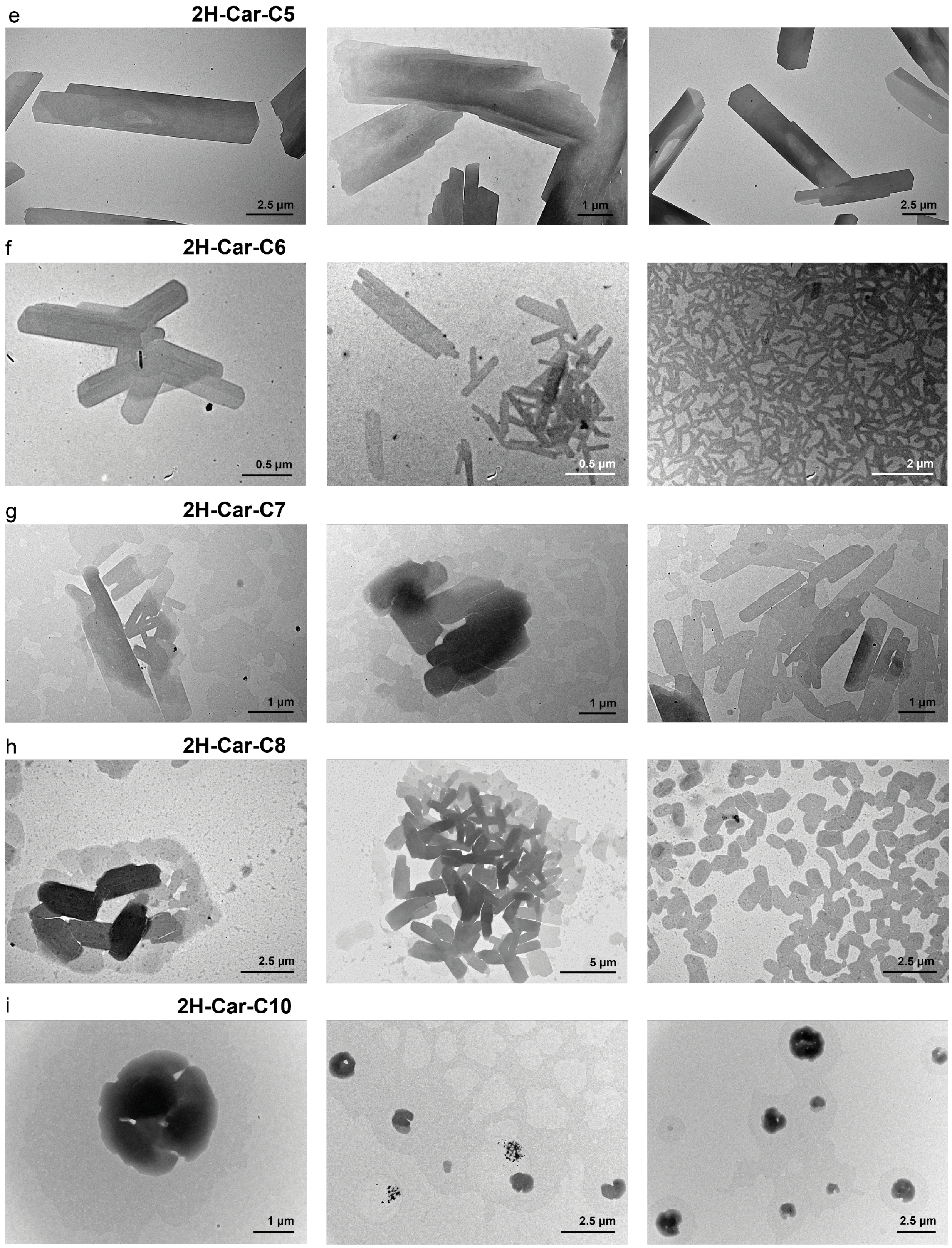

Supplementary Figure 63. TEM images of **a**, **2H-Car-C1**; **b**, **2H-Car-C2**; **c**, **2H-Car-C3**; **d**, **2H-Car-C4**; **e**, **2H-Car-C5**; **f**, **2H-Car-C6**; **g**, **2H-Car-C7**; **h**, **2H-Car-C8** and **i**, **2H-Car-C10** from a 1 mM solution in toluene.

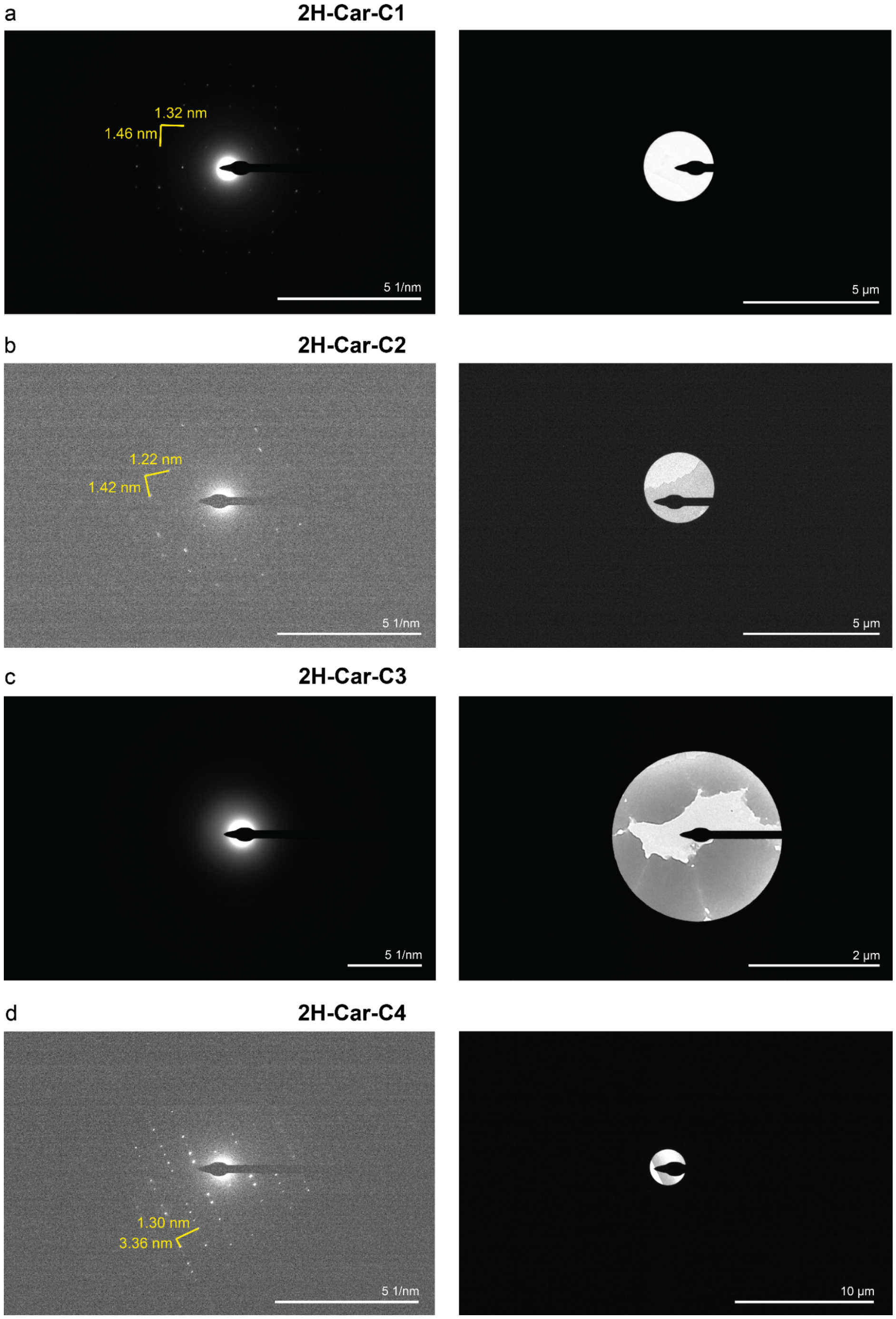

Supplementary Figure 64. Selected area electron diffraction (SAED) of **a**, **2H-Car-C1**; **b**, **2H-Car-C2**; **c**, **2H-Car-C3** and **d**, **2H-Car-C4** from respective 1 mM toluene solutions and the corresponding TEM images of the material from which the SAED patterns were obtained. We consistently observe individual diffraction spots for **2H-Car-C1**, **2H-Car-C2** and **2H-Car-C4**, showing that these supramolecular assemblies are highly ordered and crystalline. Furthermore, we can assign the distances obtained from measuring between two spots in both directions to the length of the axes from the single cell of these assemblies.

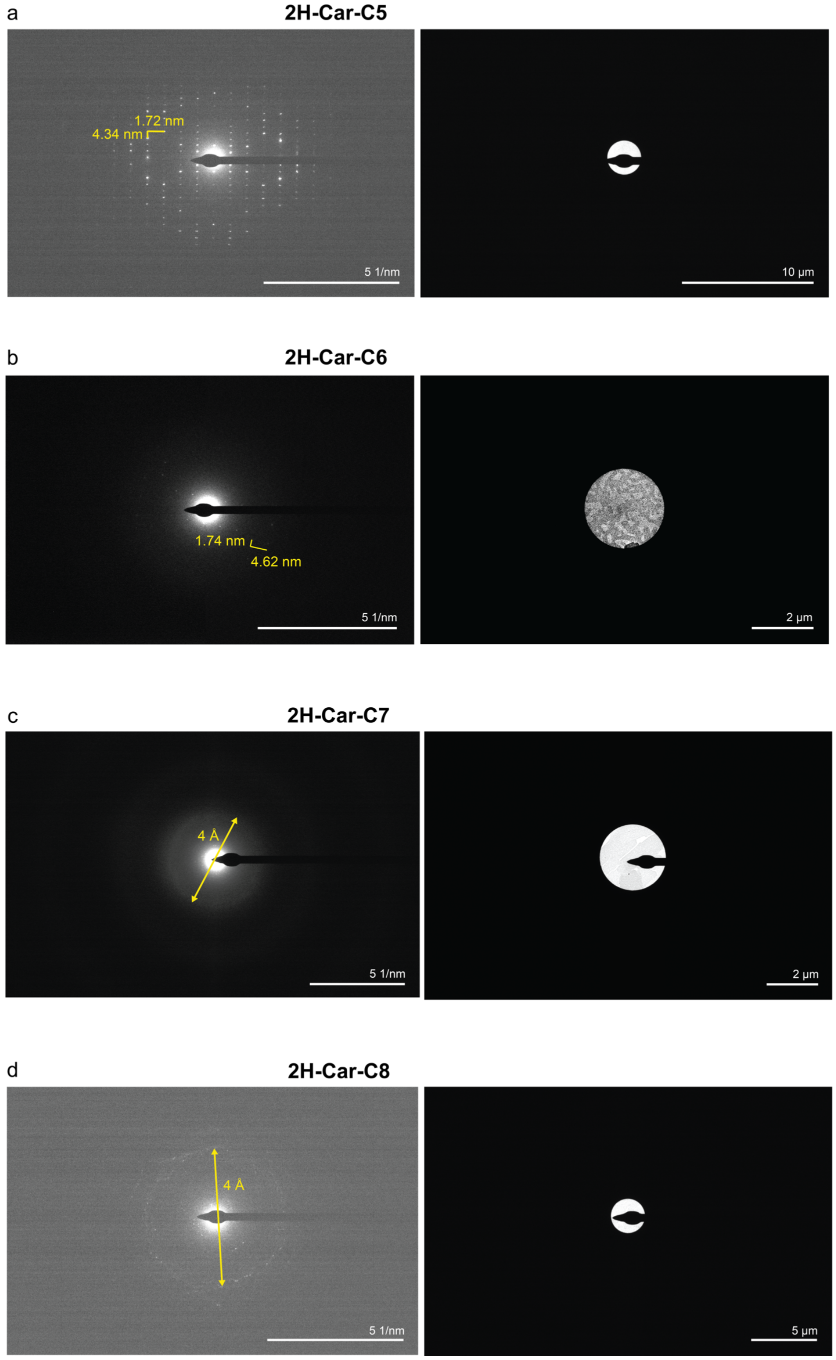

Supplementary Figure 65. Selected area electron diffraction (SAED) of **a**, **2H-Car-C5**; **b**, **2H-Car-C6**; **c**, **2H-Car-C7** and **d**, **2H-Car-C8** from respective 1 mM toluene solutions and the corresponding TEM images of the material from which the SAED patterns were obtained. We consistently observe individual diffraction spots for **2H-Car-C5**, showing that these supramolecular assemblies are highly ordered and crystalline. Furthermore, we can assign the distances obtained from measuring between two spots in both directions to the length of the axes from the single cell of these assemblies. As for **2H-Car-C6**, **2H-Car-C7** and **2H-Car-C8**, we consistently observe a diffuse halo, showing the soft matter character of these assemblies. In addition, in the case of **2H-Car-C6**, some very faint diffractions spots were also observed.

Selected AFM images

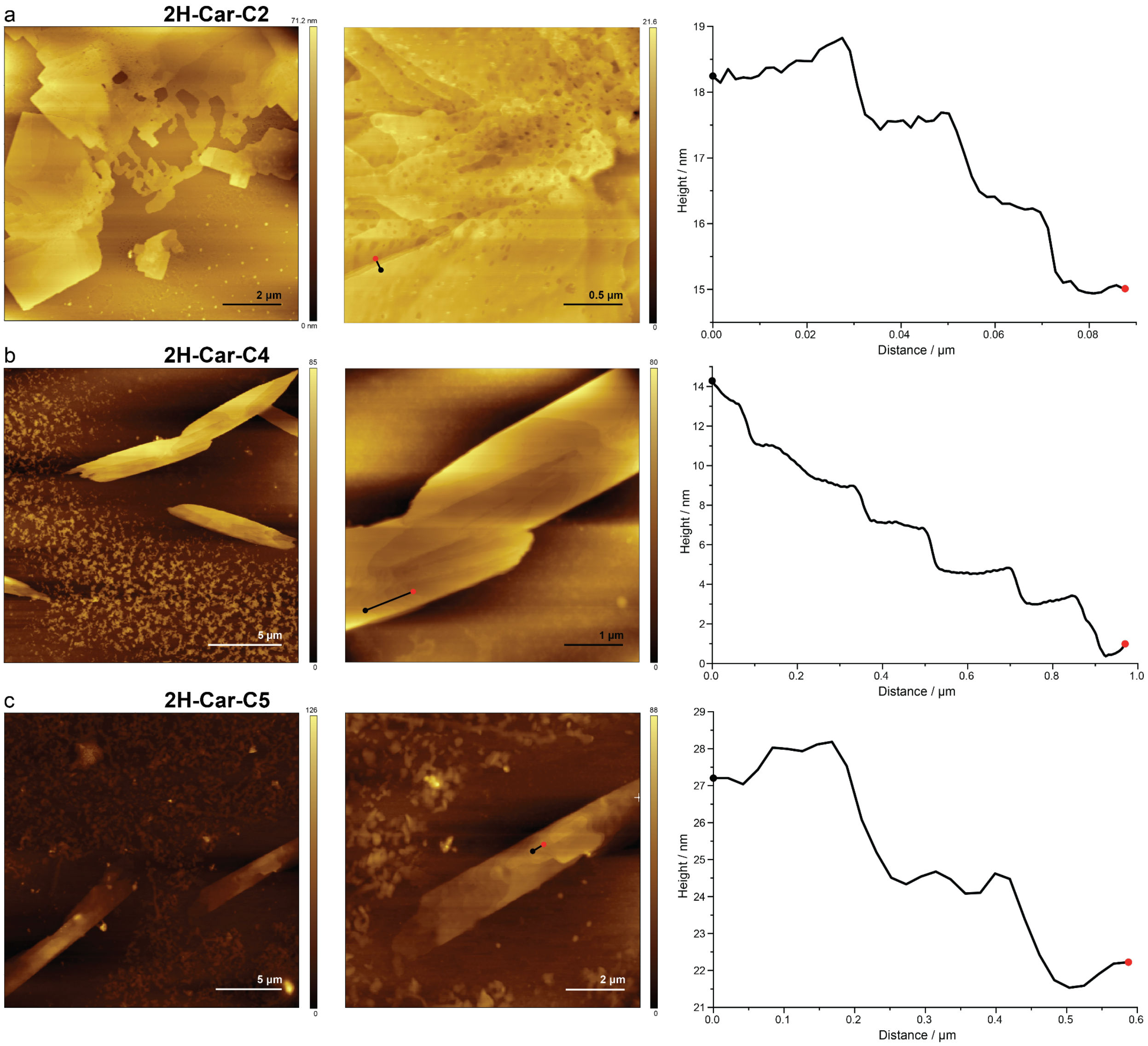

Supplementary Figure 66. Selected AFM images of **a**, **2H-Car-C2**; **b**, **2H-Car-C4** and **c**, **2H-Car-C5** from a 1 mM solution in toluene. Height profiles for the indicated areas are shown on the right.

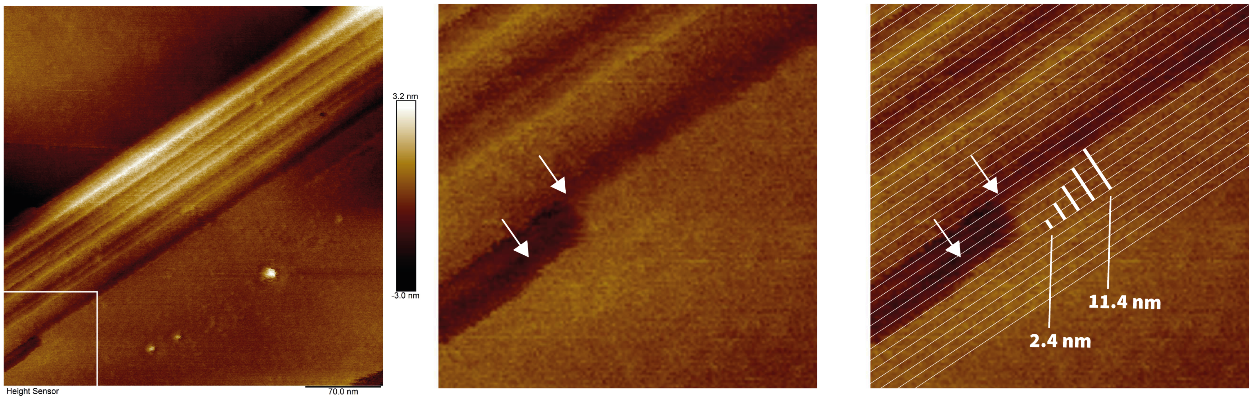

Supplementary Figure 67. Additional AFM images from **2H-Car-C6** in which individual columnar assemblies and their propagation direction can be distinguished in regions with defects, and by the step thickness of the edges.

Differential Scanning Calorimetry

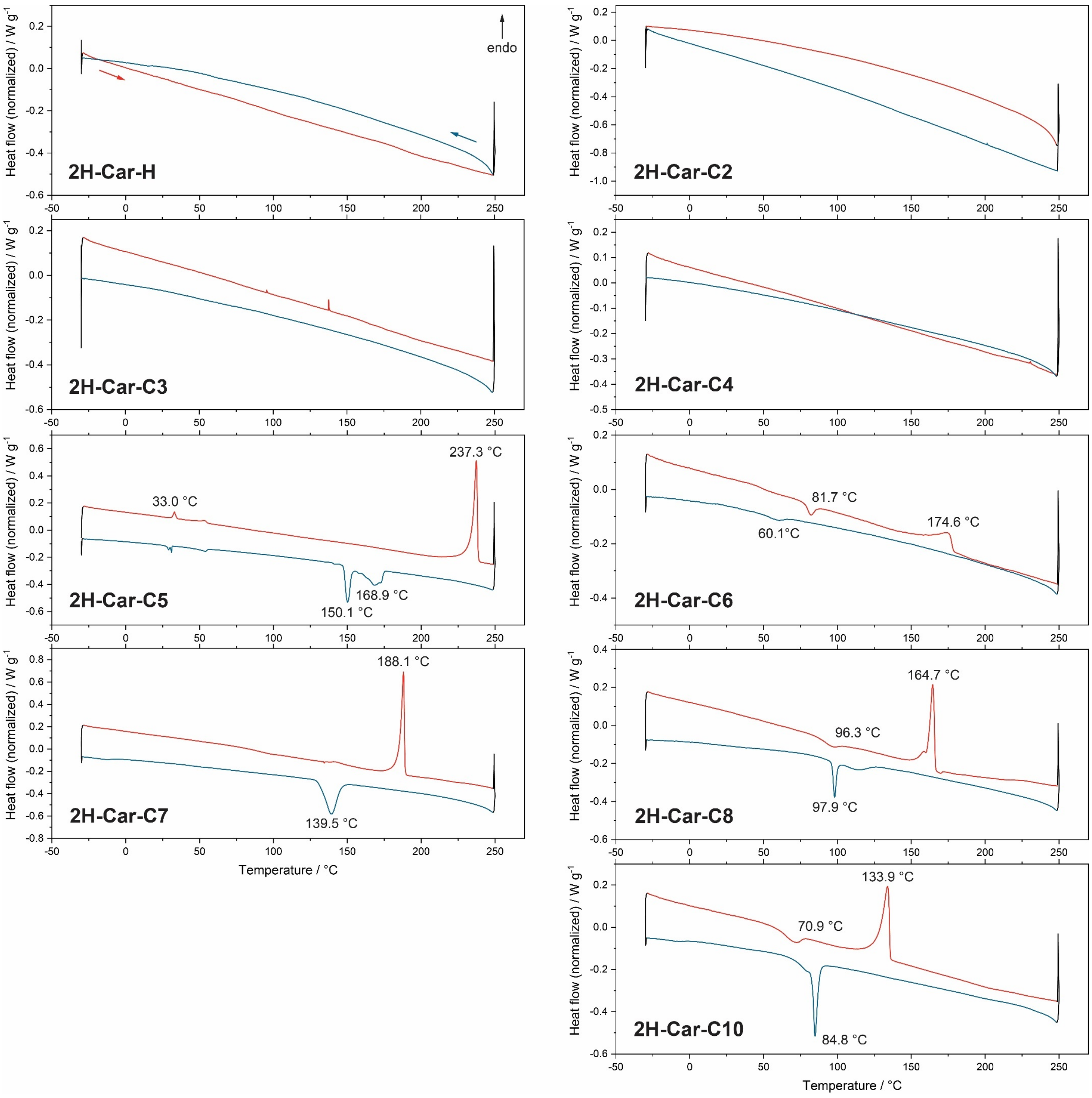

Supplementary Figure 68. DSC curves of the synthesized derivatives. Heating up (red arrow) shows that up to **2H-Car-C4** they behave like normal organic compounds that do not melt in the range of temperatures studied, and from **2H-Car-C5** to **2H-Car-C10**, they are melting within the range of temperatures, further showing that the longer the chain, the sooner the isotropic melt is observed. Cooling down (blue arrow) shows that in most cases (**2H-Car-C5** to **2H-Car-C10**) a crystallization process is observed.

X-ray Diffraction

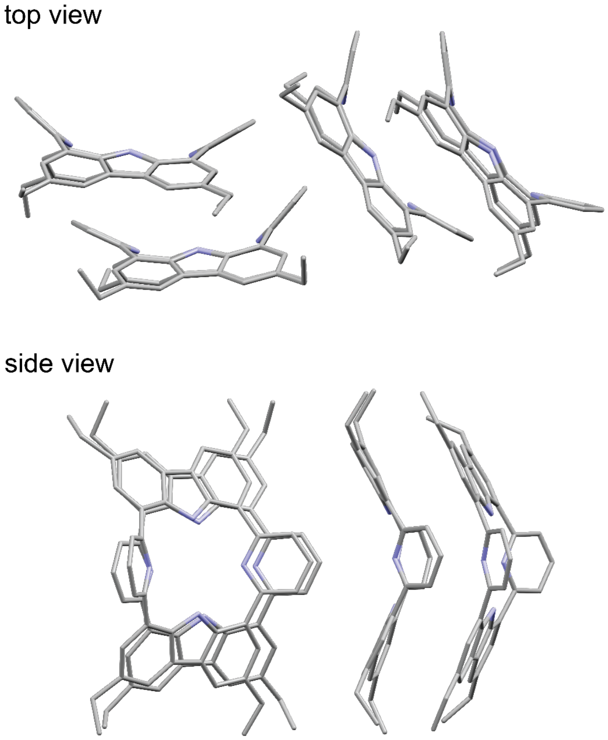

Supplementary Figure 69. Top and side view from the X-ray structure of **2H-Car-C2**. 4 carpyridine units are shown.

top view

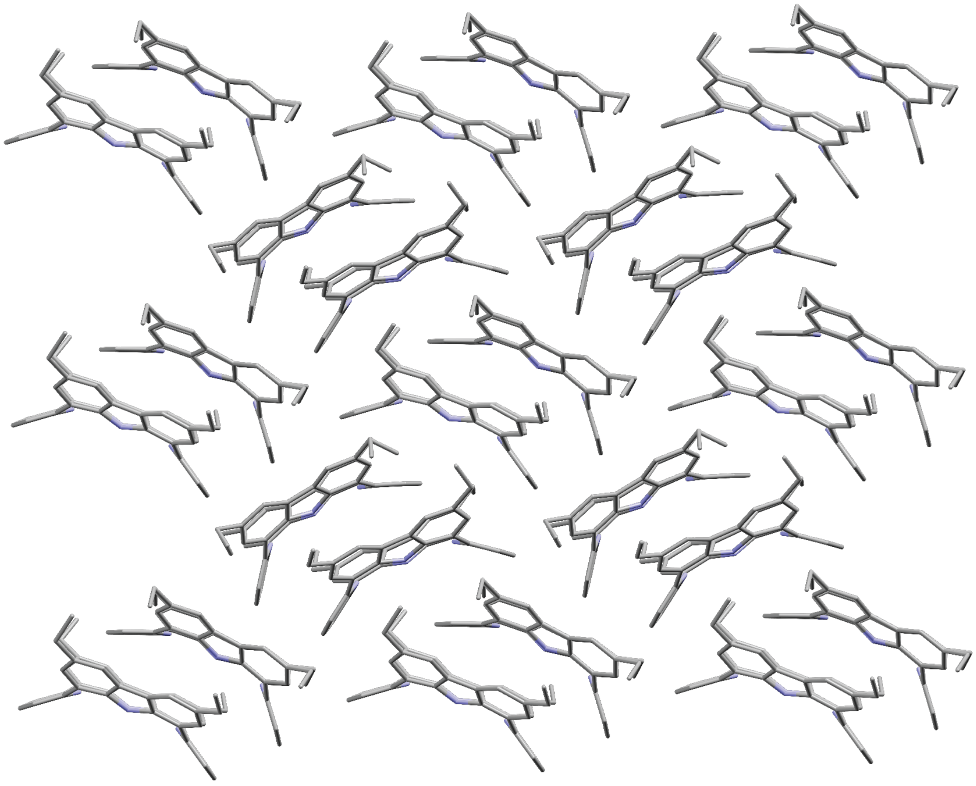

side view

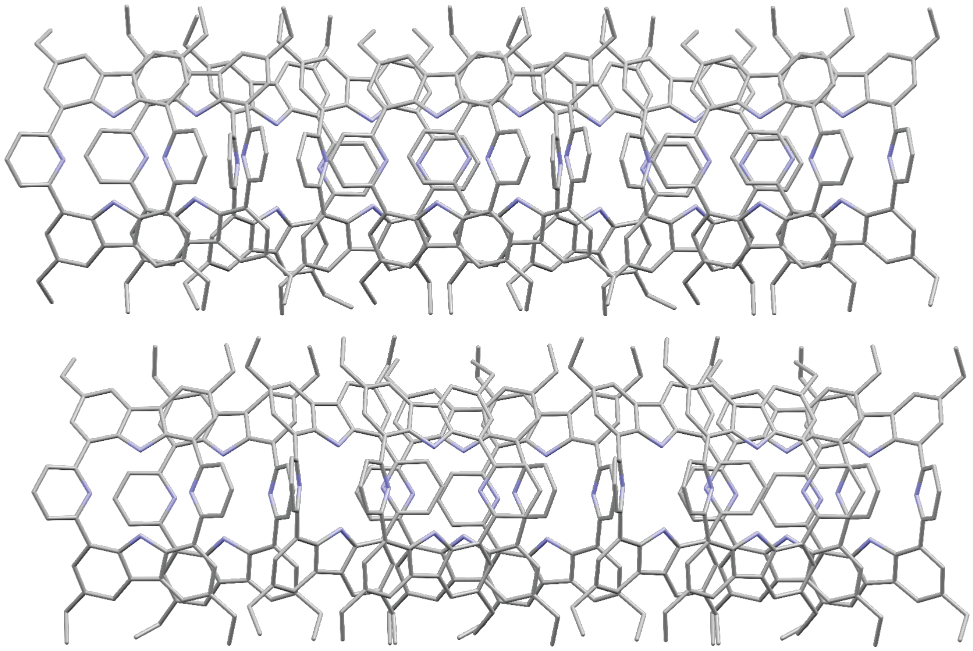

Supplementary Figure 70. Top and side view showing the packing from the X-ray structure of **2H-Car-C2**, as well as the layer segregation.

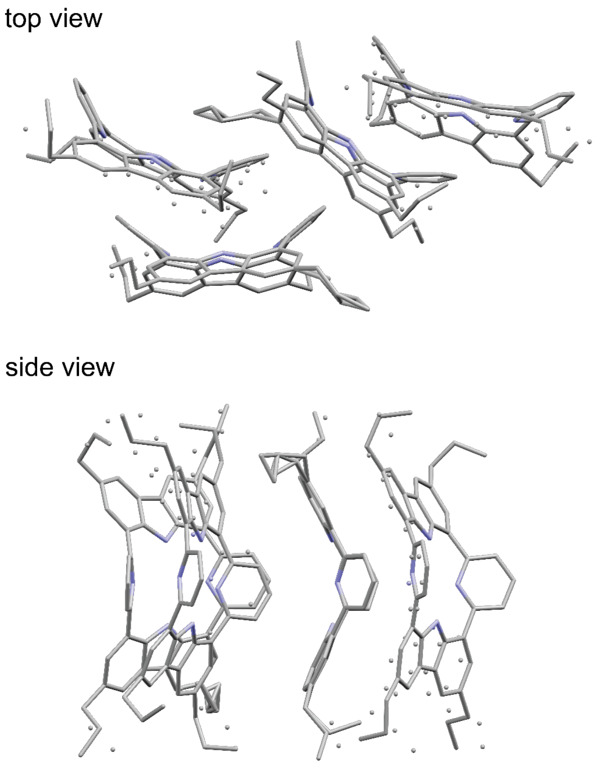

Supplementary Figure 71. Top and side view from the X-ray structure of **2H-Car-C3**. 4 carpyridine units are shown.

top view

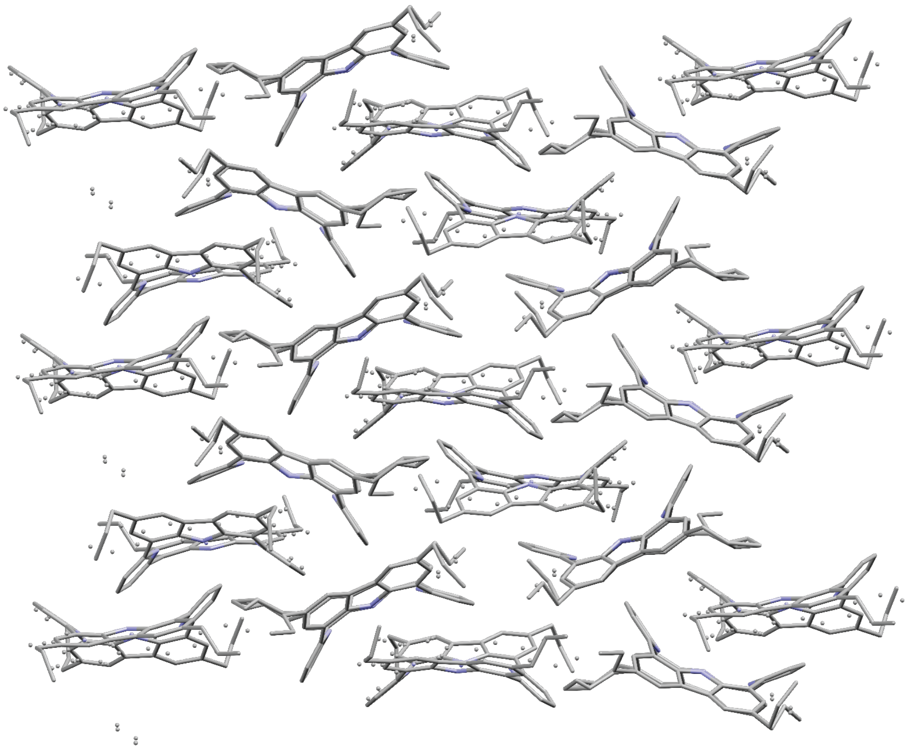

side view

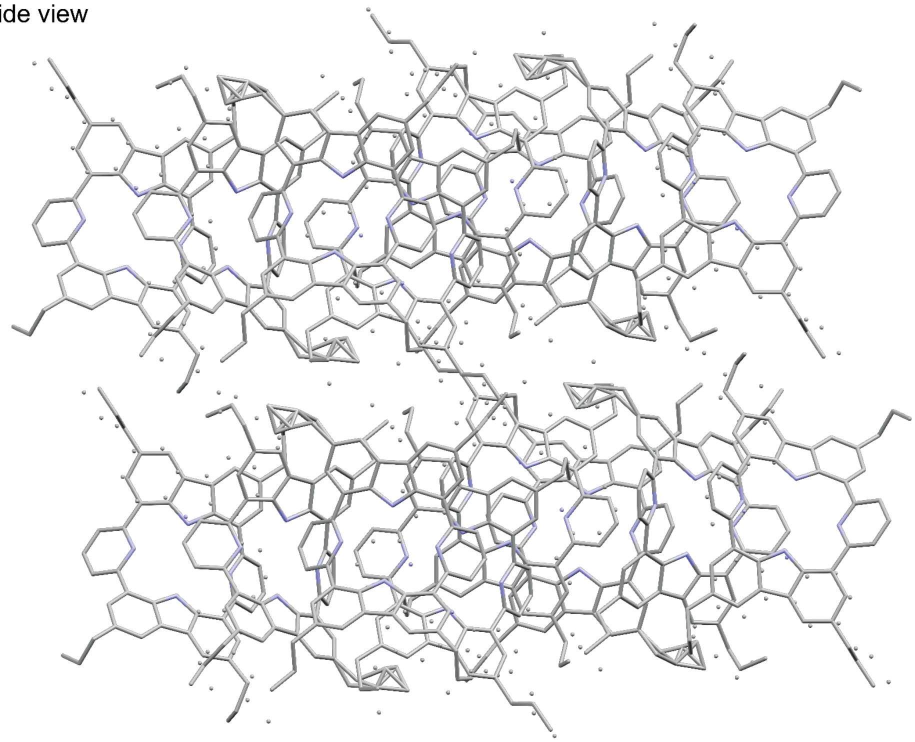

Supplementary Figure 72. Top and side view showing the packing from the X-ray structure of **2H-Car-C3**, as well as the layer segregation.

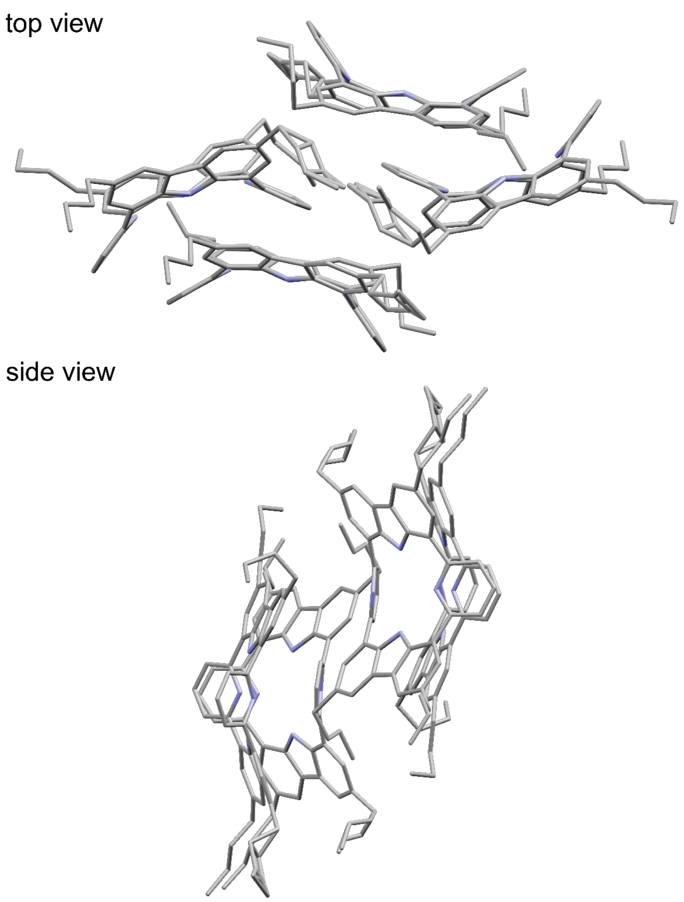

Supplementary Figure 73. Top and side view from the X-ray structure of **2H-Car-C4**. 4 carpyridine units are shown.

top view

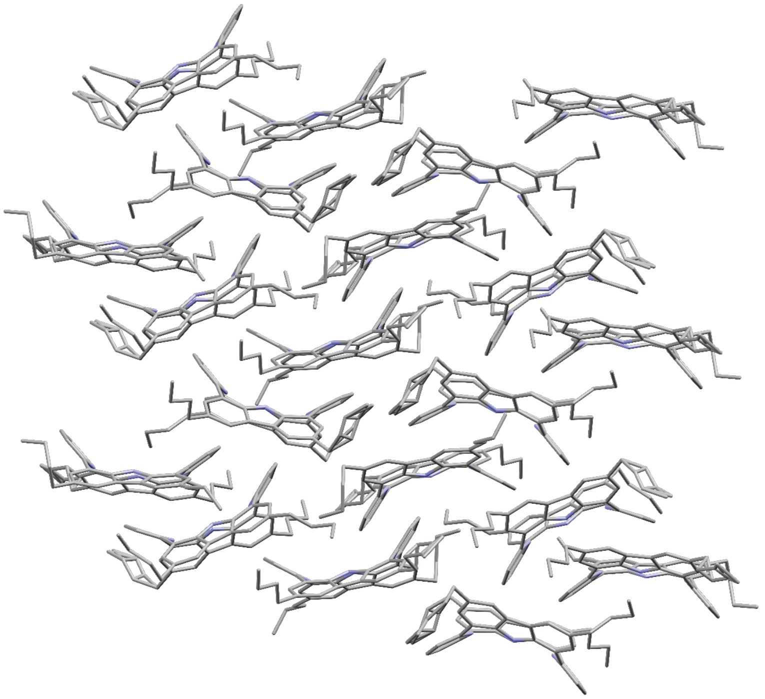

side view

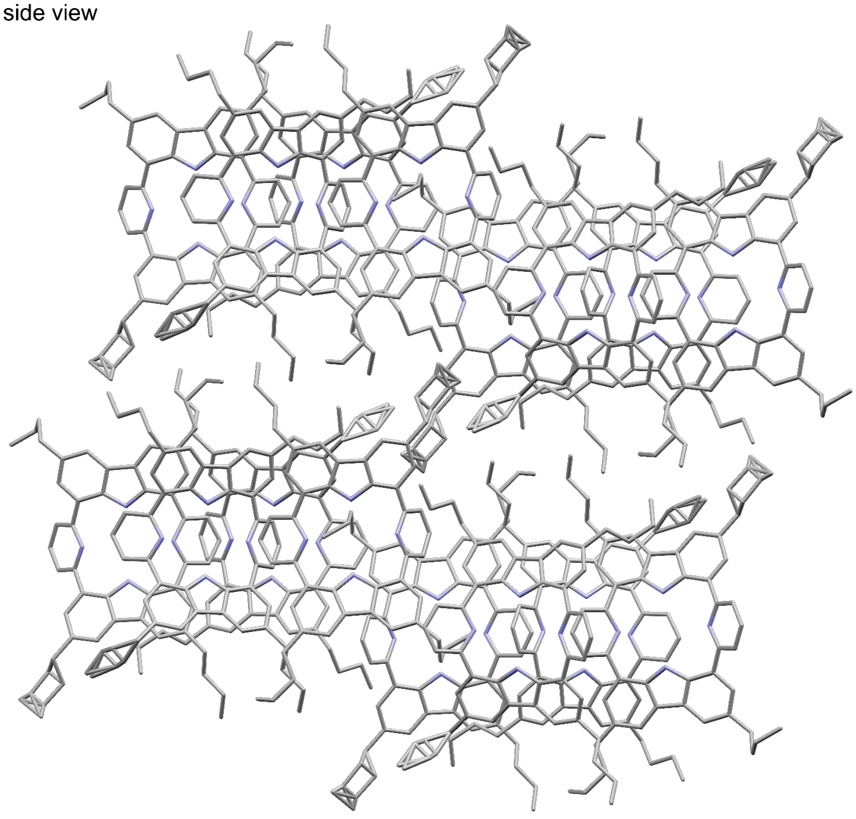

Supplementary Figure 74. Top and side view showing the packing from the X-ray structure of **2H-Car-C4**, as well as the layer segregation.

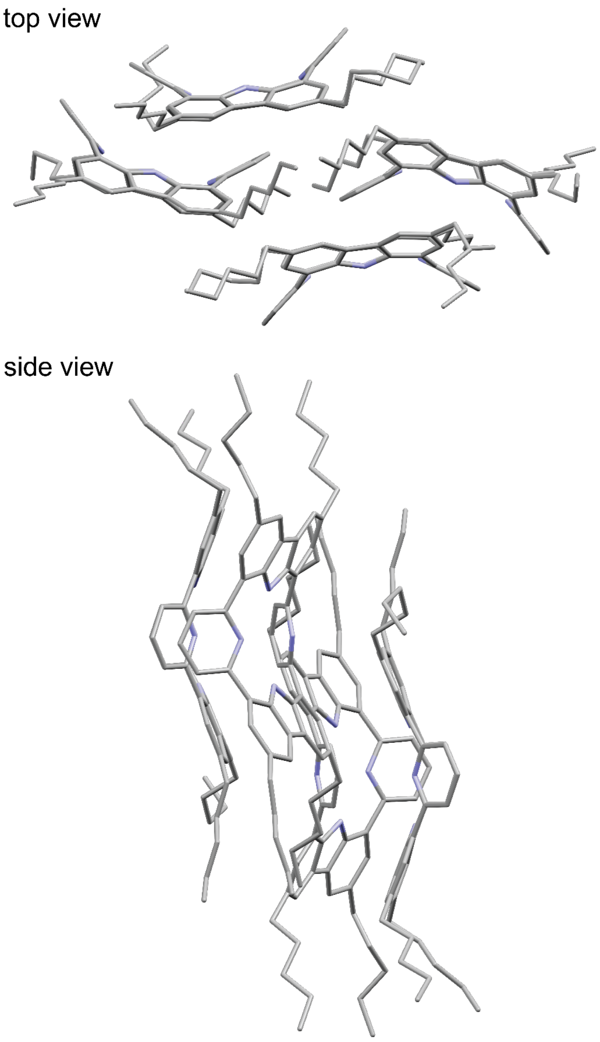

Supplementary Figure 75. Top and side view from the X-ray structure of **2H-Car-C5**. 4 carpyridine units are shown.

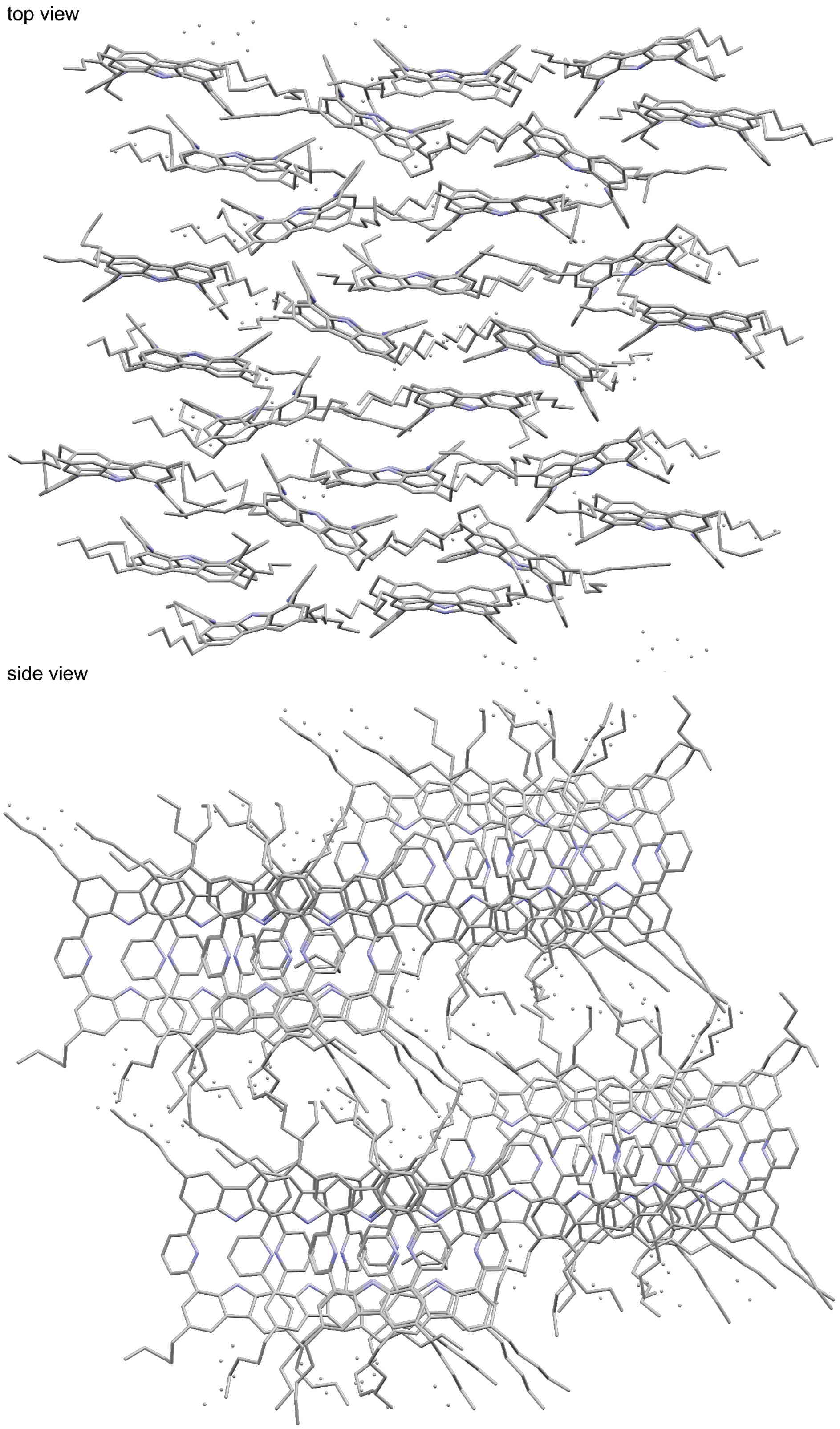

Supplementary Figure 76. Top and side view showing the packing from the X-ray structure of **2H-Car-C5**, as well as the layer segregation.

Methods

Definition of terms.

Function minimized: $\Sigma w(F_o^2 - F_c^2)^2$

where $w = [\sigma^2(F_o^2) + (aP)^2 + bP]^{-1}$ and $P = (F_o^2 + 2F_c^2)/3$

$F_o^2 = S(C - RB) / Lp$

and $\sigma^2(F_o^2) = S^2(C + R^2B) / Lp^2$

S = Scan rate

C = Total integrated peak count

R = Ratio of scan time to background counting time

B = Total background count

Lp = Lorentz-polarization factor

R-factors:

$R_{int} = \Sigma|<F_o^2> - F_o^2| / \Sigma F_o^2$ summed only over reflections for which more than one symmetry equivalent was measured.

$R(F) = \Sigma||F_o| - |F_c|| / \Sigma|F_o|$ summed over all observed reflections.

$wR(F^2) = [\Sigma w(F_o^2 - F_c^2)^2 / \Sigma w(F_o^2)^2]^{1/2}$ summed over all reflections.

Standard deviation of an observation of unit weight (goodness of fit):

$[\Sigma w(F_o^2 - F_c^2)^2 / (N_o - N_v)]^{1/2}$

where N_o = number of observations; N_v = number of variables

Crystallographic data of **2H-Car-C2** were collected at 160.0(1) K on a Rigaku-Oxford Diffraction XtaLAB Synergy-S dual source diffractometer. This is a kappa-axis four-circle goniometer with a Rigaku HyPix-6000HE HPC (Hybrid Photon Counting) detector and Cu and Mo PhotonJet microfocus X-ray sources. Suitable crystals were covered with oil (Infineum V8512, formerly known as Paratone N), placed on a nylon loop that is mounted on a CrystalCap Magnetic™ pin (Hampton Research) and immediately transferred to the diffractometer. The program suite *CrysAlis*^{Pro} was used for data collection, numerical and multi-scan absorption correction as well as data reduction[7]. The structure was solved with the charge flipping algorithm using *olex2.solve* within *Olex2* GUI[8] and was refined by full-matrix least-squares methods on F^2 with *SHELXL-2018*[9] using the *Olex2* GUI. The graphical output was produced with the help of the program *Mercury*[10]. The asymmetric unit contains four independent molecules. Three methyl groups were found to be disordered and were refined in a ratio of 50.2:49.8, 53.7:46.3 and 69.1:30.9 respectively.

Supplementary Table 7. Crystal data and structure refinement for **2H-Car-C2**.

CCDC No.	2254105
Empirical formula	$C_{42}H_{36}N_4$
Formula weight	596.75
Crystal system	Monoclinic
Space group	Pc
a / Å	12.9197(2)
b / Å	14.99166(14)
c / Å	36.9595(6)
α / °	90
β / °	121.434(2)
γ / °	90
Volume / $Å^3$	6108.01(19)
Z	8
Density (calculated) / mg m^{-3}	1.298
Temperature / K	160.00(10)
Wavelength / Å	1.54184
Absorption coefficient / mm^{-1}	0.588
F(000)	2528
Crystal size / mm^3	0.34 × 0.2 × 0.033
Crystal description	red plate
Theta range for data collection / °	2.948 to 79.875
Index ranges	$-15 \le h \le 16$, $-19 \le k \le 15$, $-46 \le l \le 47$
Reflections collected	112355
Independent reflections	24052 [R_{int} = 0.0402]
Reflections observed	22760
Criterion for observation	$I > 2 \sigma (I)$
Completeness to theta	100.0 % to 67.684°
Absorption correction	Semi-empirical from equivalents
Max. and min. transmission	1.00000 and 0.57612
Data / restraints / parameters	24052 / 17 / 1703
Goodness-of-fit on F^2	1.056
Final R indexes [$I > 2 \sigma (I)$]	R_1 = 0.0567, wR_2 = 0.1584
R indexes (all data)	R_1 = 0.0598, wR_2 = 0.1642
Absolute structure parameter	−0.09(13)
Extinction coefficient	0.00063(10)
Largest diff. peak and hole / e $Å^{-3}$	0.249 and −0.244

Crystallographic data of **2H-Car-C3** were collected at 160(1) K on a Rigaku OD Supernova-Atlas diffractometer using the copper X-ray radiation (l = 1.54184 Å) from a dual wavelength X-ray source and an Oxford Instruments Cryojet XL cooler. The selected suitable single crystal was mounted using polybutene oil on a flexible loop fixed on a goniometer head and immediately transferred to the diffractometer. Pre-experiment, data collection, data reduction and analytical absorption correction[11] were performed with the program suite *CrysAlisPro*[12]. Using *Olex2*[8], the structure was solved with the SHELXT[13] small molecule structure solution program and refined with the *SHELXL2018/3* program package[9] by full-matrix least-squares minimization on F^2. *PLATON*[14] was used to check the result of the X-ray analysis.
There are two independent molecules in the asymmetric unit. Only two terminal propyl ligands are not disordered. One of the two independent molecules is severely disordered (68%) over two sets of positions. The NH hydrogen atoms were located in Fourier difference maps but they were included in the model in riding positions.

Supplementary Table 8. Crystal data and structure refinement for **2H-Car-C3**.

CCDC No.	2254084
Empirical formula	$C_{46}H_{44}N_4$
Formula weight	652.85
Crystal system	Monoclinic
Space group	$P2_1/c$
a / Å	16.58570(10)
b / Å	13.11690(10)
c / Å	33.3647(2)
α / °	90
β / °	92.4230(10)
γ / °	90
Volume / $Å^3$	7252.10(8)
Z	8
Density (calculated) / mg m^{-3}	1.196
Temperature / K	160(1)
Absorption coefficient / mm^{-1}	0.536
F(000)	2784.0
Crystal size / mm^3	0.5 × 0.22 × 0.13
Radiation	Cu Kα (λ = 1.54184)
2Θ range for data collection / °	5.302 to 136.502
Index ranges	$-10 \le h \le 19$, $-15 \le k \le 15$, $-40 \le l \le 40$
Reflections collected	69446
Independent reflections	13260 [R_{int} = 0.0355, R_{sigma} = 0.0244]
Data / restraints / parameters	13260 / 2828 / 1285
Goodness-of-fit on F^2	1.034
Final R indexes [I ≥ 2 σ (I)]	R_1 = 0.0625, wR_2 = 0.1703
Final R indexes [all data]	R_1 = 0.0678, wR_2 = 0.1752
Largest diff. peak and hole / e $Å^{-3}$	0.46 and −0.34

Crystallographic data of **2H-Car-C4** were collected at 160(1) K on a Rigaku OD Synergy/Hypix diffractometer using the copper X-ray radiation (l = 1.54184 Å) from a dual wavelength X-ray source and an Oxford Instruments Cryojet XL cooler. The selected suitable single crystal was mounted using polybutene oil on a flexible loop fixed on a goniometer head and immediately transferred to the diffractometer. Pre-experiment, data collection, data reduction and analytical absorption correction[11] were performed with the program suite *CrysAlisPro*[12]. Using *Olex2*[8], the structure was solved with the SHELXT[13] small molecule structure solution program and refined with the *SHELXL2018/3* program package[9] by full-matrix least-squares minimization on F^2. *PLATON*[14] was used to check the result of the X-ray analysis. For more details about the data collection and refinement parameters, see the CIF file.
There are two independent molecules in the asymmetric unit. The NH hydrogen atoms were located in Fourier difference maps and freely refined.

Supplementary Table 9. Crystal data and structure refinement for **2H-Car-C4**.

CCDC No.	2254085
Empirical formula	$C_{50}H_{52}N_4$
Formula weight	708.95
Crystal system	Monoclinic
Space group	$P2_1/c$
a / Å	17.99579(6)
b / Å	13.00938(4)
c / Å	34.02888(11)
α / °	90
β / °	94.7762(3)
γ / °	90
Volume / $Å^3$	7938.98(4)
Z	8
Density (calculated) / mg m^{-3}	1.186
Temperature / K	160(1)
Absorption coefficient / mm^{-1}	0.526
F(000)	3040.0
Crystal size / mm^3	0.26 × 0.13 × 0.06
Radiation	Cu Kα (λ = 1.54184)
2Θ range for data collection / °	4.928 to 149.01
Index ranges	−15≤h≤22, −16≤k≤16, −42≤l≤42
Reflections collected	138264
Independent reflections	16239 [R_{int} = 0.0226, R_{sigma} = 0.0128]
Data / restraints / parameters	16239 / 182 / 1055
Goodness-of-fit on F^2	1.046
Final R indexes [I ≥ 2 σ (I)]	R_1 = 0.0454, wR_2 = 0.1250
Final R indexes [all data]	R_1 = 0.0495, wR_2 = 0.1307
Largest diff. peak and hole / e $Å^{-3}$	0.84 and −0.29

Crystallographic data of **2H-Car-C5** were collected at 160(1) K on a Rigaku OD Synergy/Pilatus diffractometer using the copper X-ray radiation (l = 1.54184 Å) from a dual wavelength X-ray source and an Oxford Instruments Cryojet XL cooler. The selected suitable single crystal was mounted using polybutene oil on a flexible loop fixed on a goniometer head and immediately transferred to the diffractometer. Pre-experiment, data collection, data reduction and analytical absorption correction[11] were performed with the program suite *CrysAlisPro*[12]. Using *Olex2*[8], the structure was solved with the SHELXT[13] small molecule structure solution program and refined with the *SHELXL2018/3* program package[9] by full-matrix least-squares minimization on F^2. *PLATON*[14] was used to check the result of the X-ray analysis. For more details about the data collection and refinement parameters, see the CIF file.

There are six independent molecules in the asymmetric unit. The NH hydrogen atoms were located in Fourier difference maps and freely refined. Many terminal C_5H_{12} groups are totally or partially disordered over two sets of positions.

Supplementary Table 10. Crystal data and structure refinement for **2H-Car-C5**.

CCDC No.	2254086
Empirical formula	$C_{54}H_{60}N_4$
Formula weight	765.06
Crystal system	Monoclinic
Space group	$P2_1/c$
a / Å	19.48349(10)
b / Å	37.7787(2)
c / Å	35.88039(19)
α / °	90
β / °	90.8205(5)
γ / °	90
Volume / $Å^3$	26407.4(3)
Z	24
Density (calculated) / mg m^{-3}	1.155
Temperature / K	160(1)
Absorption coefficient / mm^{-1}	0.508
F(000)	9888.0
Crystal size / mm^3	0.3 × 0.2 × 0.04
Radiation	Cu Kα (λ = 1.54184)
2Θ range for data collection / °	4.678 to 154.758
Index ranges	−24≤h≤23, −44≤k≤47, −42≤l≤45
Reflections collected	329548
Independent reflections	55903 [R_{int} = 0.0323, R_{sigma} = 0.0229]
Data / restraints / parameters	55903 / 2100 / 3557
Goodness-of-fit on F^2	1.049
Final R indexes [I ≥ 2 σ (I)]	R_1 = 0.0736, wR_2 = 0.2122
Final R indexes [all data]	R_1 = 0.0838, wR_2 = 0.2237
Largest diff. peak and hole / e $Å^{-3}$	0.88 and −0.66

Micro Electron Diffraction

Comparison of structural parameters obtained through solid state analyses

	Unit cell parameter	SAED	micro-ED	X-ray
**2H-Car-C2**	**a / Å**	12.871	-	12.9197(2)
	**b / Å**	14.981	-	14.99166(14)
	**c / Å**	-	-	36.9595(6)
	**α / °**	-	-	90
	**β / °**	-	-	121.434(2)
	**γ / °**	-	-	90
**2H-Car-C4**	**a / Å**	-	18.40(3)	17.99508(5)
	**b / Å**	12.9765	13.104(18)	13.00885(3)
	**c / Å**	33.6545	34.22(5)	34.02757(10)
	**α / °**	-	90	90
	**β / °**	-	92.587(3)	94.7763(3)
	**γ / °**	-	90	90

Supplementary Table 11. Comparison of unit cell parameters for **2H-Car-C2** and **2H-Car-C4** through SAED, micro-ED and X-ray analysis.

Comparison of structural parameters obtained through solid state analyses

	Unit cell parameter	SAED	micro-ED	X-ray[a]
**2H-Car-C5**	**a / Å**	-	19.67(13)	19.48534(10)
	**b / Å**	17.1965	17.33(11)	37.7819(2)
	**c / Å**	43.3605	44.59(29)	35.87616(17)
	**α / °**	-	90	90
	**β / °**	-	90.26(2)	90.8175(4)
	**γ / °**	-	90	90
**2H-Car-C6**	**a / Å**	17.4075	17.65	20.1809(4)
	**b / Å**	-	20.96	17.2408(2)
	**c / Å**	46.209	45.32	42.4043(7)
	**α / °**	-	91.83	90
	**β / °**	-	92.13	101.9163(18)
	**γ / °**	-	92.97	90

Supplementary Table 12. Comparison of unit cell parameters for **2H-Car-C5** and **2H-Car-C6** through SAED, micro-ED and X-ray analysis. [a] different polymorphs are observed with SAED and micro-ED to the X-ray structure.

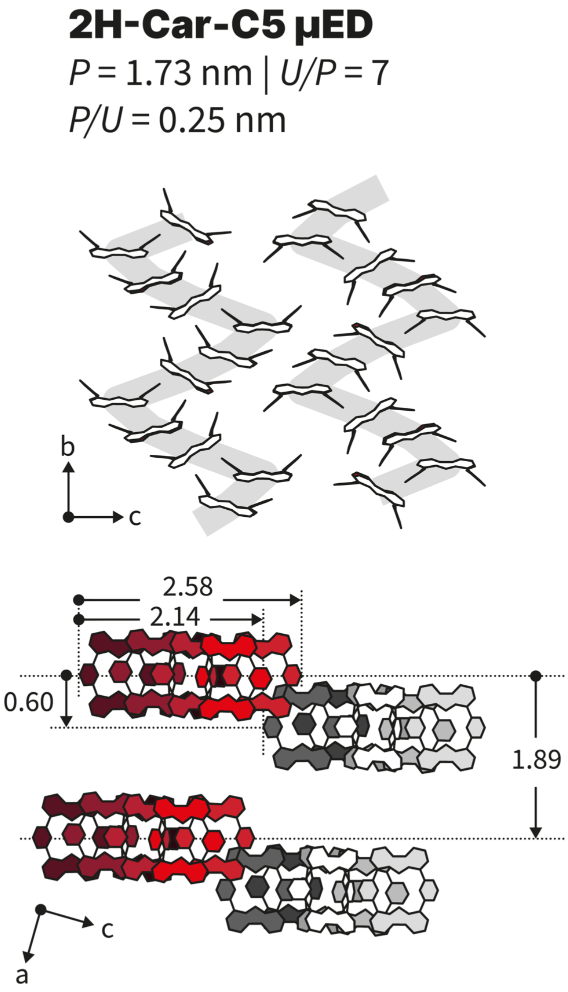

Supplementary Figure 77. Cartoon representation of **2H-Car-C5** assembly structure derived from micro-ED measurements showing a top view with antiparallel slipped columns and a side view of carpyridine layers. Sidechains are omitted for clarity. Values are given in nm.

Supplementary References

1. Woods, J. et al. Shape-assisted self-assembly. *Nat. Commun*. **13**, 3681 (2022).
2. Rowland, C. A., Yap, G. P. A. & Bloch, E. D. Novel syntheses of carbazole-3,6-dicarboxylate ligands and their utilization for porous coordination cages. *Dalton Trans*. **49**, 16340–16347 (2020).
3. Gross, D. E., Mikkilineni, V., Lynch, V. M. & Sessler, J. L. Bis-amidopyrrolyl Receptors Based on Anthracene and Carbazole. *Supramol. Chem.* **22**, 135 (2010).
4. Wang, X.-Y et al. Postfunctionalization of BN-Embedded Polycyclic Aromatic Compounds for Fine-Tuning of Their Molecular Properties. *Chem. Eur. J.*, **21**, 8867–8873 (2015).
5. Wang, P.-Y. et al. Synthesis and biological evaluation of pyridinium-functionalized carbazole derivatives as promising antibacterial agents. *Bioorg. Med. Chem. Lett.* **27**, 4294–4297 (2017).
6. Dawei, C., Yu, J., Zeng, H., Li, C.-J. Dearomatization−rearomatization strategy for synthesizing carbazoles with 2,2′-biphenols and ammonia by dual C(Ar)−OH bond cleavages. *J. Agric. Food Chem.* **68**, 13200–13205 (2020).
7. CrysAlisPro Software system; Rigaku Oxford Diffraction, vers. 1.171.42; Rigaku Corporation (2022).
8. Dolomanov, O. V., Bourhis, L. J., Gildea, R. J., Howard, J. A. K. & Puschmann, H. *OLEX2*: a complete structure solution, refinement and analysis program. *J. Appl. Cryst*. **42**, 339–341 (2009).
9. Sheldrick, G. M. Crystal structure refinement with SHELXL. *Acta Crystallogr. C: Struct. Chem*. **71**, 3–8 (2015).
10. Macrae, C. F., Sovago, L., Cottrell, S. J., Galek, P. T. A., McCabe, P., Pidcock, E., Platings, M., Shields, G. P., Stevens, J. S., Towler, M. & Wood, P. A. Mercury 4.0: from visualization to analysis, design and prediction. *J. Appl. Cryst*. **53**, 226–235 (2020).
11. Clark, R. C. & Reid, J. S. The analytical calculation of absorption in multifaceted crystals. *Acta Cryst. A* **51**, 887–897 (1995).
12. *CrysAlisPro* (version 1.171.42.57a), Rigaku Oxford Diffraction Ltd, Yarnton, Oxfordshire, England (2022).
13. Sheldrick, G. M. SHELXT – Integrated space-group and crystal-structure determination. Acta Crystallogr. A: Found. Adv. 71, 3–8 (2015).
14. Spek, A. L. Structure validation in chemical crystallography. *Acta Cryst. D* **65**, 148–155 (2009).
